# Mesenchymal state of intimal cells may explain higher propensity to ascending aortic aneurysm in bicuspid aortic valves

**DOI:** 10.1038/srep35712

**Published:** 2016-10-25

**Authors:** Shohreh Maleki, Sanela Kjellqvist, Valentina Paloschi, Joelle Magné, Rui Miguel Mamede Branca, Lei Du, Kjell Hultenby, Johan Petrini, Jonas Fuxe, MIBAVA Leducq Consortium, Harry C. Dietz, Bart Loeys, Lut Van Laer, Andrew S. McCallion, Luc Mertens, Seema Mital, Salah A. Mohamed, Gregor Andelfinger, Janne Lehtiö, Anders Franco-Cereceda, Per Eriksson, Hanna M. Björck

**Affiliations:** 1 Cardiovascular Medicine Unit, Center for Molecular Medicine, Department of Medicine, Karolinska Institutet, Karolinska University hospital Solna, Stockholm, Sweden; 2 Science for Life Laboratory, Department of Biochemistry and Biophysics, Stockholm University, Stockholm, Sweden; 3 Department of Oncology-Pathology, Science for Life Laboratory, Karolinska Institutet, Stockholm, Sweden; 4 Department of Laboratory Medicine, Karolinska Institutet, Stockholm, Sweden; 5 Clinical Physiology, Department of Molecular Medicine and Surgery, Karolinska Institutet, Stockholm, Sweden; 6 Department of Microbiology, Tumor and Cell biology, Karolinska Institutet, Stockholm, Sweden; 7 Cardiothoracic Surgery Unit, Department of Molecular Medicine and Surgery, Karolinska Institutet, Stockholm, Sweden; 8 McKusick-Nathans Institute of Genetic Medicine, Johns Hopkins University School of Medicine, Baltimore, USA; 9 Department of Medical Genetics, University of Antwerp and Antwerp University Hospital, Antwerp, Belgium; 10 Cardiology, The Hospital for Sick Children, University of Toronto, Toronto, ON, Canada; 11 Hospital for Sick Children, University of Toronto, Toronto, ON, Canada; 12 Department of Cardio and Thoracic Vascular Surgery, Universitaetsklinikum Schleswig-Holstein, Campus Luebeck, Germany; 13 Sainte Justine University Hospital Research Center, Université de Montréal, Montréal, QC,, Canada

## Abstract

Individuals with a bicuspid aortic valve (BAV) are at significantly higher risk of developing aortic complications than individuals with tricuspid aortic valves (TAV) and defective signaling during the embryonic development and/or life time exposure to abnormal hemodynamic have been proposed as underlying factors. However, an explanation for the molecular mechanisms of aortopathy in BAV has not yet been provided. We combined proteomics, RNA analyses, immunohistochemistry, and electron microscopy to identify molecular differences in samples of non-dilated ascending aortas from BAV (N = 62) and TAV (N = 54) patients. Proteomic analysis was also performed for dilated aortas (N = 6 BAV and N = 5 TAV) to gain further insight into the aortopathy of BAV. Our results collectively showed the molecular signature of an endothelial/epithelial-mesenchymal (EndMT/EMT) transition-like process, associated with instability of intimal cell junctions and activation of RHOA pathway in the intima and media layers of ascending aorta in BAV patients. We propose that an improper regulation of EndMT/EMT during the spatiotemporally related embryogenesis of semilunar valves and ascending aorta in BAV individuals may result in aortic immaturity and instability prior to dilation. Exasperation of EndMT/EMT state in post embryonic life and/or exposure to non-physiological hemodynamic could lead to the aneurysm of ascending aorta in BAV individuals.

Bicuspid aortic valve (BAV) is the most common congenital heart malformation present in 1–2% of the human population. Individuals with a BAV are at significantly higher risk of developing serious aortic complications than individuals with tricuspid aortic valves (TAVs). The coincidence of valve malformation and ascending aortic aneurysm formation has been proposed to be genetically determined^1^ and the consequence of a common defect arising at an early stage of cardiac embryogenesis^2^. An increased hemodynamic burden imposed by BAV morphology itself has also been discussed as an alternative causal factor for fragility of ascending aorta^3^.

The development of semilunar valves (aortic and pulmonic valves) and that of the primordial aorta from the outflow tract (OFT) are related processes, involving complex, yet not fully characterized, molecular interactions. The first manifestation of embryonic valve development is the formation of endocardial cushions in the OFT by endothelial/endocardial-mesenchymal transition (EndMT). During this process, a subset of endocardial cells delaminates and transform into mesenchymal cells and invades the cardiac jelly. Septation of the OFT into the ascending aorta and the pulmonary trunk is spatiotemporally coupled to the maturation of cushions into elongated semilunar valves, and is achieved by interaction between OFT cushion, second heart field (SHF) progenitors and migratory cardiac neural crest cells (NCCs)^2^,^4^.

Similar to epithelial-mesenchymal transition (EMT), EndMT can also be re-activated in adult life under certain pathological conditions, such as wound healing, cancer, and fibrotic disease^5^. The initiation of EMT and EndMT is driven by well-characterized transcription factors, such as SNAIL1/2, ZEB1/2 and TWIST, which transform epithelial and endothelial cells (ECs), respectively, into a mesenchymal state by inducing transcriptional reprogramming. EndMT/EMT is divided into three subtypes representing distinct biological processes, resulting in production of different cell types—i.e., mesenchymal cells in the case of type I, fibroblasts in the type II, and invasive tumor cells in the type III. The hallmark of all types is the disassembly of cell-cell junctions—the change in polarity and motile behavior of the cells by reorganization of the cytoskeleton^6^.

Our previous transcriptomic and protein data have shown that the development of aneurysm in BAV and TAV patients takes place via different molecular processes. In BAV, aneurysm formation involve substantially fewer differentially expressed genes and proteins, denoting a more gradual and stepwise molecular change in aortic walls^7^,^8^. In order to gain further insight into the aortopathy of BAV, we performed an unbiased proteomic comparison between non-dilated, as well as dilated, BAV and TAV patients. Interestingly, the proteomics data, followed by KEGG/Hallmark/Ingenuity pathway analysis, revealed a molecular signature in the BAV non-dilated aorta resembling the activation of EMT or EndMT during cancer metastasis and cardiac cushion development, respectively. We propose that the aggravation of this process contributes to the development of aneurysm in BAV. Using the available knowledge, we further compared the distribution of some key players of EMT/EndMT in non-dilated aortic walls of BAV and TAV patients.

## Results

### Proteomic analysis of non-dilated ascending aortas showed increased EndMT activity in BAV patients

The novel liquid chromatography-mass spectrometry-based proteomic approach, HiRIEF LC-MS/MS^9^, which is an unbiased discovery method with deep proteome coverage, allowed the detection and quantification of 2894 proteins in the intima-media of non-dilated and dilated ascending aortas. Two-dimensional PCA analysis together with supervised OPLS analysis of the proteomic data showed that TAV and BAV samples formed two different clusters (Fig. S1, Supplementary Table 3) emphasizing that aortic tissues in TAV and BAV had different protein expression profiles. To identify the proteins responsible for the clustering of non-dilated samples, jack-knife confidence levels derived from cross-validation of the OPLS analysis were calculated. Significance was calculated as ABS (loading) – ABS (jack-knife confidence interval); positive values indicated significant proteins. This resulted in identification of 276 differentially expressed proteins between BAV-ND and TAV-ND (Supplementary Table 4a).

KEGG and Hallmark analysis of the 276 proteins revealed endocytosis, apoptosis, EMT and pathways related to focal adhesion and ECM. Activated biochemical pathways included glycolysis, hypoxia, oxidative phosphorylation, TCA cycle and fatty and amino acid metabolism, i.e. metabolic processes induced during cell transformation (Supplementary Table 5a,b). Moreover, Ingenuity pathway analysis of the 276 differentially expressed proteins (Supplementary Table 6), combined with literature search of the 30 most up- and downregulated proteins in BAV-ND (Supplementary Tables 7 and 8 for up- and downregulated proteins, respectively) revealed enrichment of pathways and proteins involved in protein degradation and trafficking, cell junction dynamics, apoptosis, cell cycle and cancer related biological processes. The highest scoring pathway in the Ingenuity analysis was acute phase response, which is activated in inflammation and tissue remodeling. Differential expression of proteins related to this pathway in combination with LXR/RXR has recently been described in colon adenocarcinoma^10^. The literature search reconfirmed the results mentioned above.

We further performed proteomic analysis on dilated BAV and TAV aorta. Totally, 805 proteins were significantly differentially expressed between BAV-D and TAV-D (Supplementary Table 4b). Emerging pathways in KEGG and Hallmark analysis included regulation of cytoskeleton, autophagy related mTORC1, and cell junctions related pathways (tight and apical junction) (Supplementary Table 5c,d). Differential activation of leukocyte transendothelial migration could be due to inflammation or endothelial trafficking, and dysfunction resulted by disintegration of EC junctions. The top pathway in Ingenuity analysis of the 805 differentially expressed proteins was related to cytoskeletal reorganization, followed by several RHO-associated processes, endocytosis and cell trafficking, cell junction associated pathways, and biological pathways known to be activated in cancer i.e. PI3K/AKT, IL8, aryl hydrocarbon receptor, CXCR4, Cdc42, and PAK (Supplementary Table 6). Of particular interest was inclusion of pathways activated during the delamination, EMT, and migration of NCCs during cardiac development—namely, signaling by Rho family GTPases, ephrin receptor signaling, and integrin signaling^11^.

**Figure 1:**
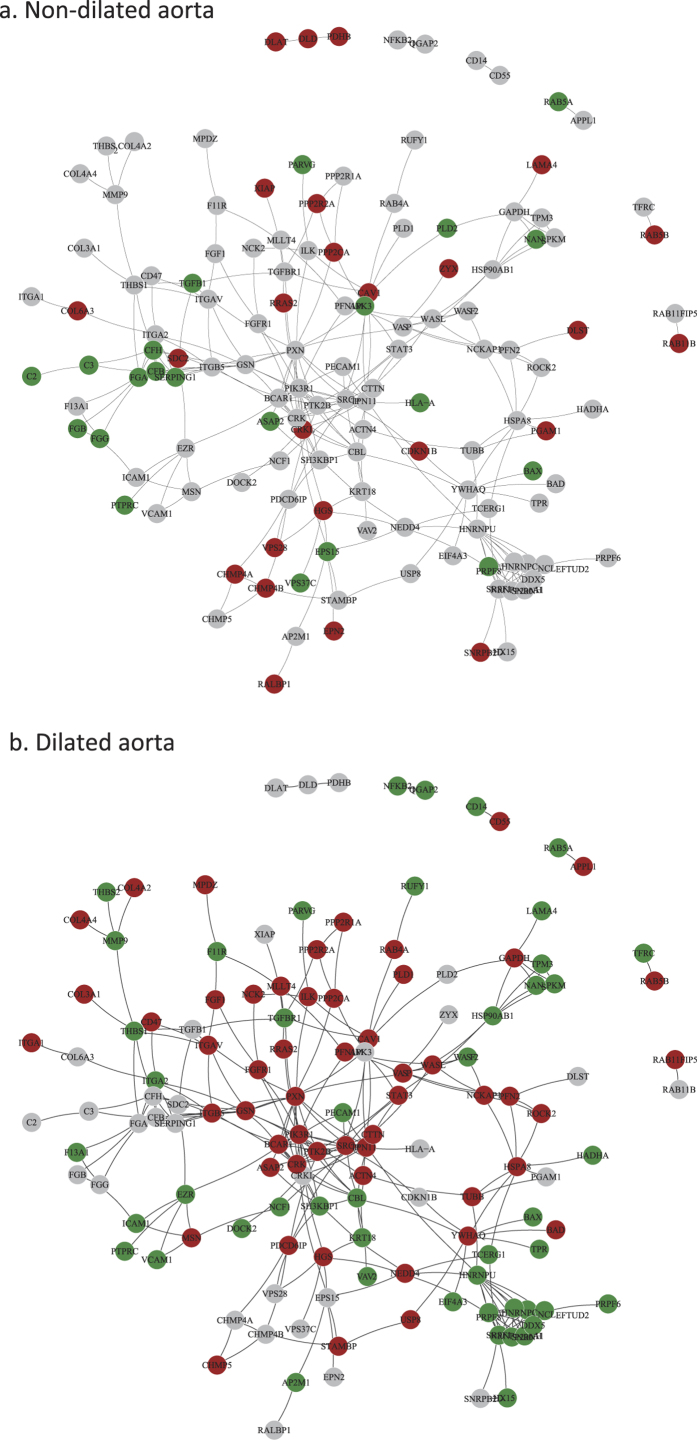
Protein–protein interactions (PPIs) network of proteins involved in enriched KEGG pathways shown in Table S5a,c. Interactions are retrieved from Human Protein Reference Database (HPRD)^46^. Proteins are colored according to the direction of expression change in non-dilated samples and dilated samples respectively. Red indicates up-regulated in BAV compared to TAV; Green indicates down-regulated in BAV and grey indicates unchanged.

We further constructed networks of interacting proteins from the KEGG pathway (Fig. 1) and Hallmark analysis (Fig. S2) for non-dilated and dilated samples. Major pathways are separately shown in higher magnification in Fig. S3. As can be seen in Figs 1 and S2, the EMT-signature was exasperated in the dilated aorta of BAV patients (red indicates up-regulated genes).

As induction of angiogenesis in aneurysm has been reported^12^ and partial EndMT^13^ has been described in angiogenesis and would be likely in the vessel wall, we investigated the mRNA expression for VEGF family ligands, receptors, and co-receptors (Supplementary Table 9, age-corrected data from Folkersen *et al*.^7^). Interestingly, several family members showed either significance (*VEGFR1*, *VEGFC*) or tendency (*VEGFA*, *VEGFR2*) for downregulation in BAV-ND. Notably, most key players in angiogenesis (*VEGFA*, *VEGFC*, *VEGFR1*, *NRP1*, *NRP2*) were significantly downregulated in dilated aorta of BAV patients, ruling out the induction of angiogenesis in BAV ascending aortas. NRP1 and NRP2 are important factors in migration of NCC^11^ and were significantly downregulated at protein level in BAV-D aorta.

Considering the mechanistic overlap between EMT in cancer and EndMT/EMT in cardiac cushion/NCC during the embryonic development of OFT, and excluding the activation of angiogenesis, these results strongly suggests an ongoing EMT/EndMT-like mechanism in the ascending aorta of BAV.

Although the intima layer of the aortic wall is primarily exposed to the abnormal hemodynamic caused by BAV morphology, the state and integrity of that in human BAV has been largely unexplored. Additionally, endothelial specific mutations in certain genes gave rise to a BAV in animal models^14^,^15^, and in the case of endothelial specific *GATA5* deletion where one fourth of the progeny carried a BAV, impaired EC migration and/or differentiation was proposed to be a contributing factor^14^. Thus, with particular focus on intima we studied and compared the EndMT in BAV-ND and TAV-ND individuals.

### Endocytosis is activated in BAV-ND patients

**Figure 2:**
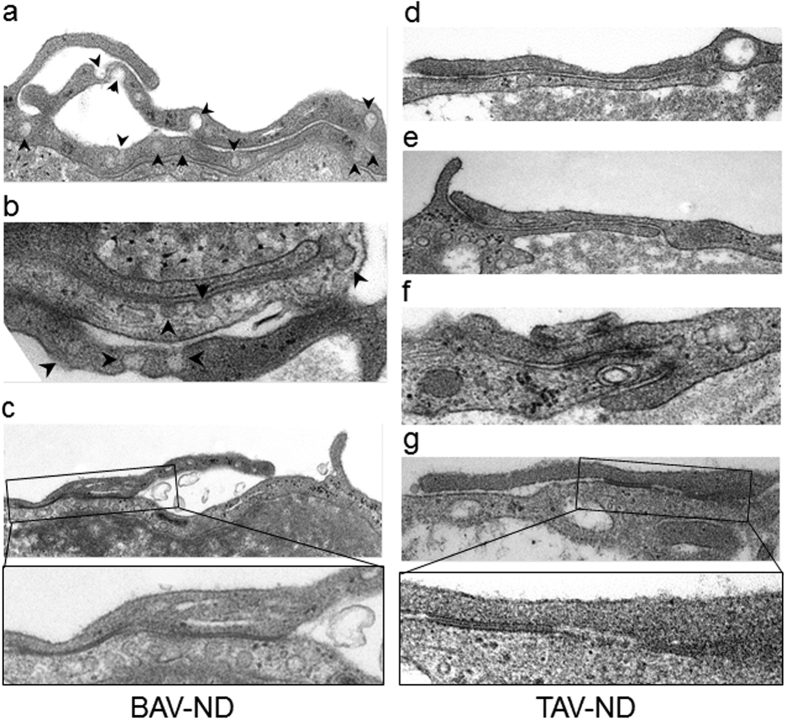
Increased endocytosis and degrading material in BAV-ND EC junctions. Representative EM images of BAV-ND (**a–c**) and TAV-ND (**d–g**) aorta. Arrowheads point to pinocytotic vesicles, N = 3 patients/group.

Using electron microscopy, we studied the ultrastructural differences between BAV-ND and TAV-ND intima. A distinctively wider gap between EC junctions, with high accumulation of endocytic vesicles adjacent to the degrading material, was observed in BAV-ND (Fig. 2a–g). Cells in patients with dilated aorta were in general more degenerated, and extensive degradation of junction material forming large gaps and EC phagocytizing adjacent cells was observed (Fig. S4), implying that junction fragility is a feature of BAV before as well as after dilation.

**Figure 3:**
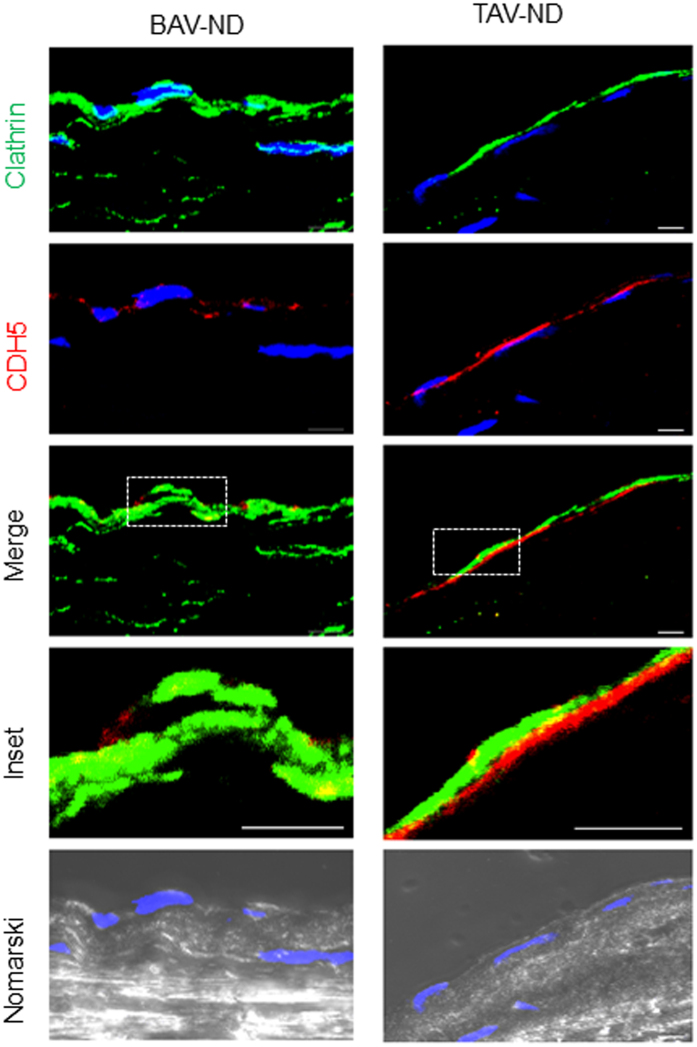
Increased endocytosis in the intima of BAV-ND, with dense accumulation of clathrin-containing material and lower expression of CDH5. Clathrin (green) and CDH5 (red). Nuclei are stained with DAPI (blue). Differential interface contrast microscopy or Nomarski (gray). Scale bar = 5 μm, N = 4 patients/group.

Clathrin-dependent endocytosis is a major pathway for the recycling of junctional complexes during cancer progression and EndMT^16^. To investigate and compare endocytic activity, confocal microscopy using anti-clathrin antibody was performed. We observed a more dense accumulation of clathrin-containing material and lower expression of CDH5, with a few co-localizing spots, in the intima of BAV-ND relative to TAV-ND (Fig. 3).

### EC junctions are unstable in BAV-ND patients

**Figure 4:**
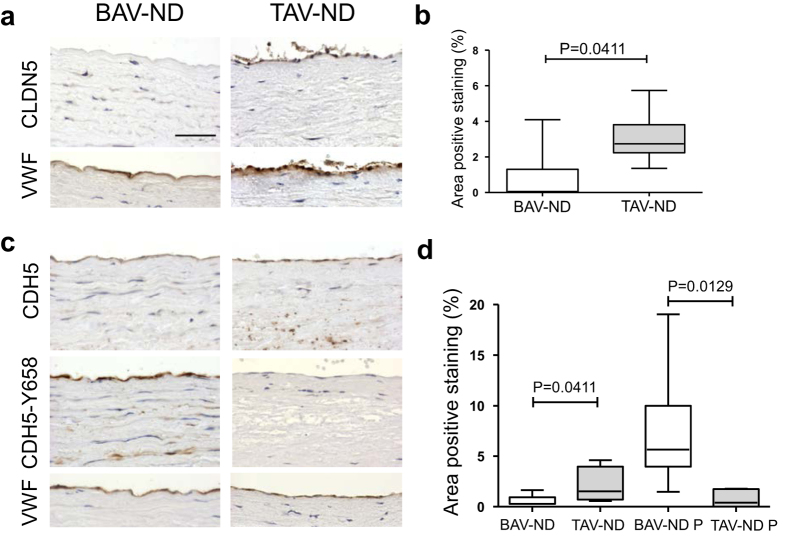
EC junctions are unstable in BAV-ND patients. Representative images of CLDN5 (**a,b**), CDH5, CDH5-Y658 (**c,d**) staining. VWF staining shows the location of endothelium. Scale bar = 50 μm, N = 7–9 patients/group. Results are expressed as mean ± SD (Mann-Whitney U-test).

A central event in initiation of EndMT is the dissociation of cell-cell junctions^6^. The staining of tight junction protein Claudin 5 (CLDN5) was noticeably reduced in the endothelium of BAV-ND compared to TAV-ND (Fig. 4a,b). Furthermore, phosphorylation is one of the major signals for the turnover of adhesion junction protein VE-cadherin (CDH5). Among tyrosine residues, Y658 phosphorylation has been intensively studied and phosphorylation at this site was shown to unmask the signal for CDH5 internalization^17^,^18^, causing the destabilization of the junctions^19^. In addition, phosphorylation at this site regulated the transition of EC to a mesenchymal state^20^. Staining BAV-ND and TAV-ND patients with anti-CDH5 and anti-Phos-CDH5-Y658 showed significantly lower CDH5 and higher Phos-CDH5-Y658 protein expression (Fig. 4c,d) in the BAV-ND intima. This observation implied that the lower expression of CDH5 in BAV-ND could be due to the CDH5 phosphorylation at Y658 enhancing its turnover.

Tyrosine phosphorylation at residue Y658 has also been associated with angiogenic stimulation^21^. The neo-vascularization areas in the aortic wall of the same sections were used as an internal control for anti-CDH5 and anti-CDH5-Phospho-Y658 antibodies. No difference was observed between BAV-ND and TAV-ND in these regions, indicating that the intensity of EC staining with anti-CDH5-Phospho-Y658 in BAV was not due to artifacts (Fig. S5).

In parallel to increased tyrosine phosphorylation, down-regulation of PTPRB phosphatase reduces permeability, and agents such as VEGF that induce endothelial permeability cause dissociation of PTPRB and CDH5^22^. We compared the distribution of PTPRB in relation to cadherin phosphorylation in consecutive sections of BAV-ND and TAV-ND. As expected, staining with CDH5-phospho Y658 increased in parallel to decreased PTPRB staining (Fig. S6). Supplementary Table 9 shows that *CDH5* gene expression was also significantly lower in dilated aorta of BAV compared to TAV, while the mesenchymal marker *N-cadherin* (*CDH2*) was higher in dilated BAV aorta. The expression of another adhesion molecule *PECAM1* was also lower in BAV dilated aorta. Notably, both CHD2 and PECAM1 protein expression showed the same pattern as gene expression in BAV-D.

**Figure 5:**
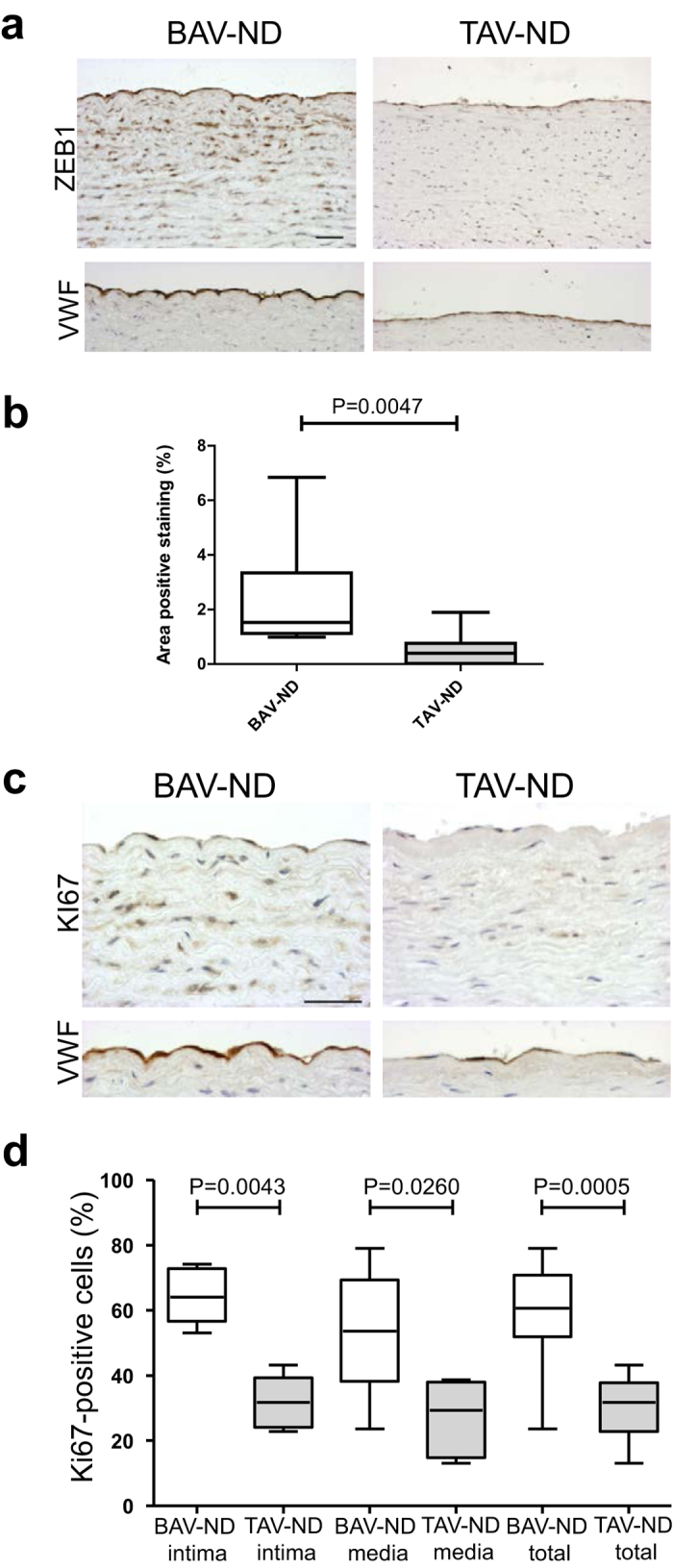
Higher expression of EMT/EndMT inducing transcription factor ZEB1 and proliferation marker KI67 in BAV-ND. Staining of ZEB1 (**a,b**); and KI67 (**c,d**). VWF staining shows the location of endothelium. Scale bars = 50 μm, N = 9 patients/group. Results are expressed as mean ± SD (Mann-Whitney U-test).

ZEB1 is the master transcription factors of EMT/EndMT, and functions primarily by targeting and repressing cadherin^23^,^24^. ZEB proteins play a role in the control of cell proliferation and survival via regulation of cell cycle-related genes. As shown in the Supplementary Materials, the level of ZEB1 activation was significantly higher in BAV-ND relative to TAV-ND, and the intima-media layer in BAV-ND patients was significantly more proliferative than TAV-ND, as judged by the number of KI67-positive nuclei (Figs 5 and S7). The higher proliferation of the aortic walls in the BAV-ND patients supports the previous finding that smooth muscle cells (SMC) may be more “immature” in BAV-ND compared to TAV-ND^2^.

Altogether, lower expression as well as higher degradation of junction material is indicative of intimal instability in BAV-ND.

### Enhanced activation of RHOA in aortic wall of BAV-ND patients

Activation of RhoA GTPase family is a crucial step in the progression of EndMT^6^ via alteration of cellular functions related to adhesion, permeability, migration, and mechanical properties, in ECs and SMCs. Several RHO related pathways were among the top 40 selected by Ingenuity analysis of dilated patients (Supplementary Table 6).

**Figure 6:**
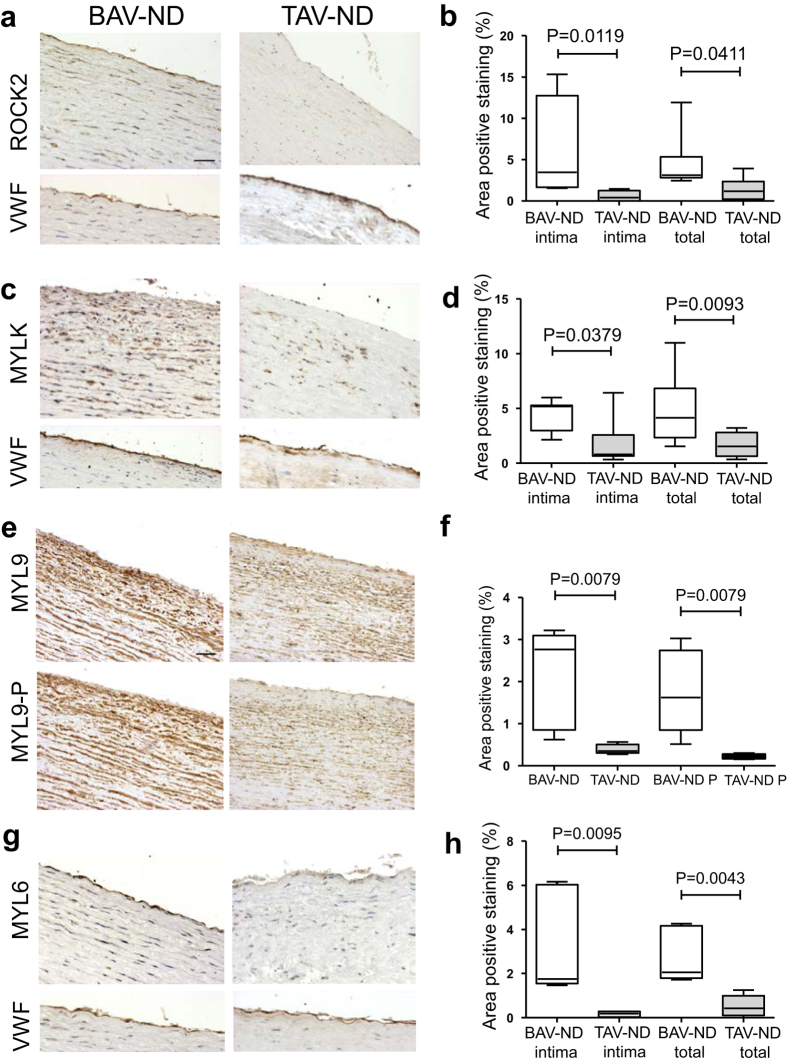
Enhanced activation of RHOA in aortic wall of BAV-ND patients. Representative images of major RHOA kinases, ROCK2 and MYLK) (**a–d**), and their targets MYL9 (**e,f**) and MYL6 (**g,h**). VWF staining shows the location of endothelium. Scale bars = 50 μm, N = 6–8 patients/group. Results are expressed as mean ± SD (Mann-Whitney U-test).

We studied the distribution of the two major RHOA kinases, Rho-associated, coiled coil-containing kinase (ROCK2) and the Ca2+-calmodulin-dependent myosin light chain kinase (MYLK). Both ROCK2 (Fig. 6a,b) and MYLK (Fig. 6c,d) protein showed a higher expression in the intima and media layer of BAV-ND compared to TAV-ND patients. Further, a distinct up-regulation of ROCK2 was observed in the intima layer of BAV patients (*P* = 0.0119, Fig. 5b). In dilated BAV, *ROCK2* mRNA and protein were significantly upregulated (Supplementary Table 9). MYLK mRNA was significantly higher in dilated patients, and confirmed to be up-regulated by using Western blot analysis in BAV-ND patients (Fig. S8c,d).

MYL9 encodes the 20-kD regulatory subunit of SMC-specific myosin II (R LC20) and regulates myosin phosphorylation and actomyosin contractile activity. MYL9 was substantially up-regulated in SMCs of BAV-ND patients (Fig. 6e,f). Use of the antibody directed against the serine 19-phosphorylated form of MYL9 (MYL9-P) also revealed a stronger staining of media in BAV-ND relative to TAV-ND (Fig. 6e,f). The proportion of MYL9/MYL9P was 1.5 times higher in BAV-ND compared to TAV-ND, implying that MYL9 was both up-regulated and more phosphorylated in BAV-ND. The *MYL6* gene encodes the 17-kD myosin II essential light chains and is expressed both in non-muscle cells and SMCs. MYL6 showed a higher expression in both endothelium and SMCs of BAV-ND compared to TAV-ND patients (Fig. 6g,h), which was further confirmed by proteomics (Supplementary Table 4a).

**Figure 7:**
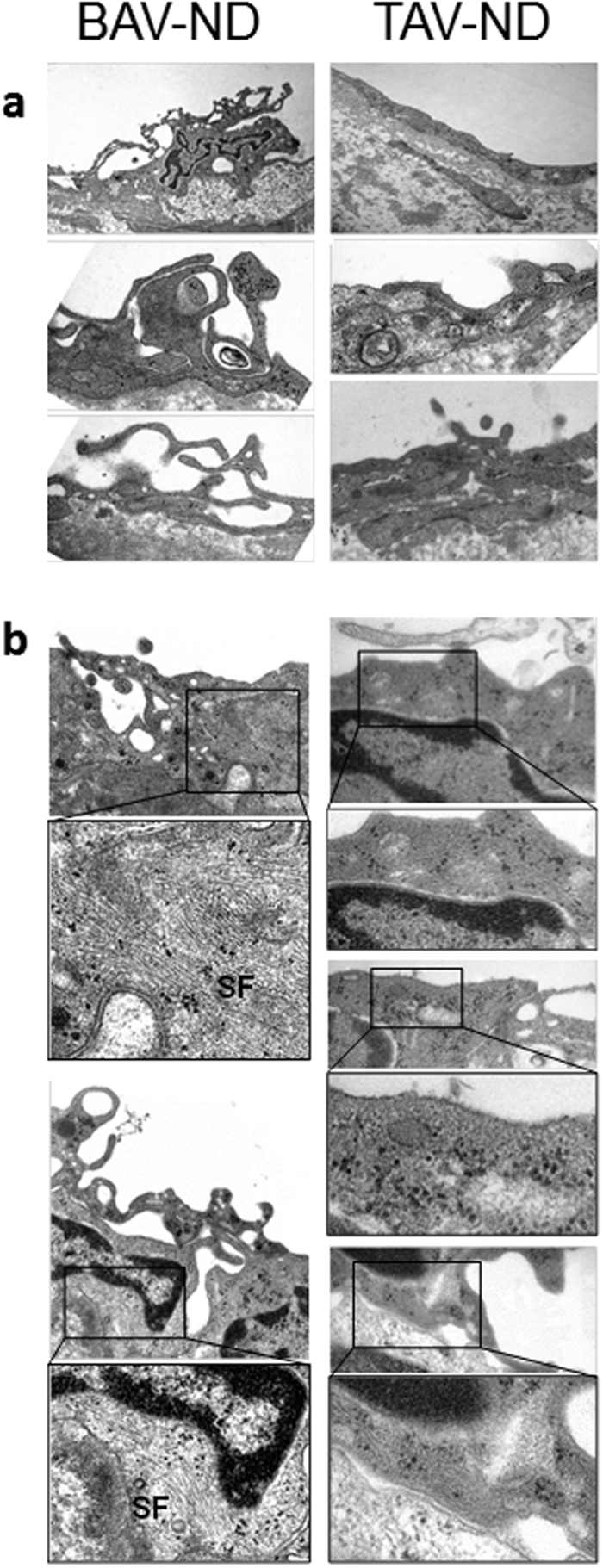
Increased pseudopodia and stress fiber formation in the endothelium of BAV patients prior to dilatation. Representative EM images of pseudopodia (**a**) and stress fibers, SF (**b**), N = 3 patients/group.

Ultrastructural studies of EC showed a complex pattern of pseudopodia formation in BAV-ND individuals, while in TAV-ND they were either absent or were much less extended (Fig. 7a). Moreover, stress fibers were more abundant in the endothelium of BAV-ND patients (Fig. 7b), which was consistent with higher expression of *ZYX* (Supplementary Table 7).

We concluded that the RHOA pathway was activated in intima-media of BAV-ND, causing the formation of pseudopodia and stress fibers in intima, features that are compatible with mesenchymal transition.

### NOTCH is activated in BAV-ND patients prior to dilation

**Figure 8:**
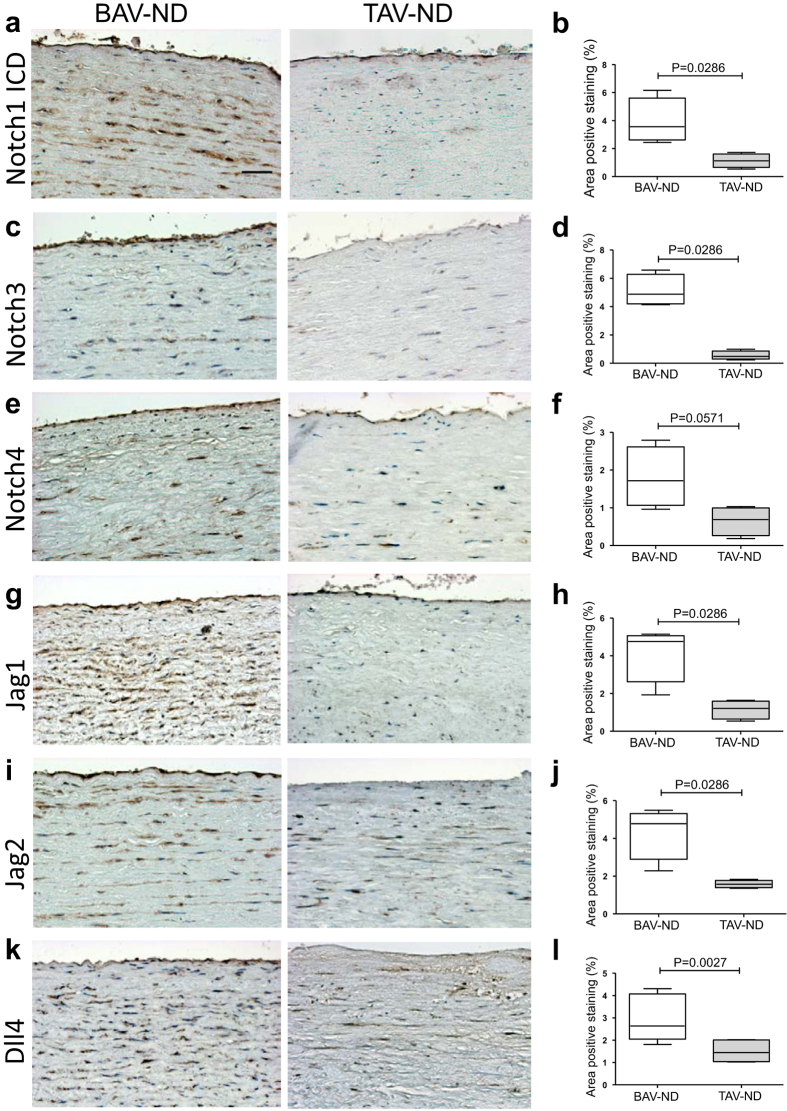
NOTCH is activated in BAV ascending aorta prior to dilation. Representative images of NOTCH receptors (**a–f**) and ligands (**g–l**). Scale bar = 50 μm, N = 7–10 patients/group. Results are expressed as mean ± SD (Mann-Whitney U-test).

Fine tuning of NOTCH ligand-receptor interaction, either by ligand endocytosis activated by E3 ubiquitin ligase Mind Bomb (*MIB1)* or by post translational modification of receptor via glycosyltransferase Manic Fringe (*MFNG*) in endocardium was reported to be required for the proper EndMT of cardiac cushion and formation of aortic valves in mice^25^. Using IHC, we showed a significantly higher expression of NOTCH3 and NOTCH1 intracellular domain (ICD), the ligands JAG1, JAG2, and DLL4 and a higher tendency for NOTCH4 expression (*P* = 0.0571) in intima-media of BAV-ND compared to TAV-ND (Fig. 8a-l). However, the NOTCH family receptors and ligands gene expression was not significantly different in non-dilated patients, indicating the post translational regulation of NOTCH signaling in BAV-ND. *MIB1* gene in BAV-ND and *MIB1* gene and protein were upregulated in BAV-D. Western blot analysis of intima-media extracts from BAV-ND and TAV-ND aortas (N = 8 + 8 = 16) showed lower protein expression of MFNF in BAV-ND patients (Fig. S8a,b). *MFNG* gene was also significantly downregulated in BAV-D compared to TAV-D (*P* < 0.00001, Supplementary Table 9). This was in line with the data published in mice^25^, supporting the regulatory role of MIB1 and MFNF for NOTCH pathway in BAV.

### Expression of cell junction and RHOA and NOTCH pathway-related proteins in internal thoracic artery

To investigate whether the observed differences were specific for the ascending aorta, internal thoracic arteries of the same patients were stained with antibodies used for aortic intima-media (CLDN5, CDH5, CDH5-Y658, MYLK, ROCK2, MYL9, MYL9-P, MYL6, NOTCH1-ICD, NOTCH3, JAG1 and DLL4). No significant difference between BAV-ND and TAV-ND patients was detected for any of the proteins (Figs S9–S12), implying the differences being aorta-specific.

## Discussion

We combined proteomic/KEGG/Hallmark/Ingenuity pathway analysis and literature search with IHC/confocal/electron microscopy to identify differential expression of proteins and cellular reorganization in non-dilated aorta of BAV and TAV patients. We provide evidence for ongoing processes compatible with the initial phase of EMT/EndMT in the ascending aorta of BAV-ND individuals, i.e. endocytosis, changes in focal adhesion, as well as induction of metabolic pathways known to be activated in EndMT/EMT/cancer. With focus on the intima, cytological data further confirmed EC junction instability and reorganization of cytoskeleton, features compatible with the induction of EndMT^5^. Proteomics studies of dilated aortic samples further strengthened that the initiation of protein trafficking, and a shift to cancer related metabolic pathways in BAV-ND, culminated in restructuring of cell junctions and cytoskeleton and induction of several cancer-related pathways. We propose that theses structural alteration can be of fundamental importance for instability of ascending aortas in BAV individuals.

The induction of EndMT/EMT in the ascending aorta of BAV could either be due to the life time exposure of aorta to non-physiological hemodynamic, and/or defective embryonic development. Disturbed flow has been shown to induce EndMT both *in vivo* and *in vitro*^26^,^27^, as well as influencing the state of cell junctions^18^ and the expression of RHO^28^. Furthermore, EMT/EndMT has a fundamental role in the development of semilunar valves, both during the formation of cardiac cushion from endocardium^5^ and in NCC delamination and migration from the neural epithelium, via either a full scale or partial EMT^11^. Either decreased^15^,^29^ or excessive^30^ EndMT of endocardial cushion resulted in the defective embryonic development of semilunar valves and OFT.

During cardiogenesis, the development of semilunar valves and septation of the outflow tract into the ascending aorta and the pulmonary trunk are spatiotemporally coupled. Recent studies using lineage specific animal models have addressed the molecular nature of this inter-connection and support the hypothesis that the deficient signal exchange between the endocardial cushion, SHF and NCC, at early stage of valve formation, is the link between malformation of valves and anomalies appearing in the aortic wall^4^. One example of such cross talk has recently been revealed for the NCC-derived SEMA3C that could activate endothelial NRP1 expression in the OFT to initiate EndMT, and that this communication was indispensable for the initiation of endocardial EndMT and promotion of OFT septation^31^. In those few transgenic animal studies where the abnormal development of semilunar valves and the pathological state of ascending aorta were both in focus, the impaired signal exchange between endocardial cushion, NCC and SHF gave rise to the defective development of the ascending aorta in parallel to valve malformations, confirming the importance of the cross talk^4^,^30^,^32^.

Due to the inconsistencies of biomarker expression, characterization of the EMT/EndMT process is complex, particularly *in vivo* and in human patient material, where the disease has most probably altered the tissues and the possibility of manipulation is non-existent. The molecular signature of EndMT/EMT can differ depending on the subtype, the tissue of origin (endothelial vs. epithelial cells), and signaling context. Nevertheless, the analysis of the proteomics data complemented with different types of microscopy and RNA analysis led us to conclude that the activation of endocytosis and RHO pathway, the instability of cell junctions, and induction of cancer-like metabolic reprogramming in intima and most probably in media of BAV-ND ascending aorta is the initial stage of an EMT/EndMT process. Further comparison of proteomic data between dilated and non-dilated patients indicated that the cellular and metabolic changes observed in non-dilated aorta culminated in the dilated state as alteration of cytoskeleton and cell polarity, cell junction instability and appearance of the new biological signaling that are even more explicitly involved in cancer/EMT/EndMT. The collective outcome of changes associated with EMT/EndMT would give rise to cells with mesenchymal characteristics and inherent instability in the aorta that may aggravate, contributing to aneurysm formation. Higher protein and mRNA expression of N-Cadherin, a fundamental biomarker of EMT/EndMT in BAV-D patients, further supports this conclusion.

Focusing on the intima in BAV-ND, we discovered cytological evidence for high endocytosis activity at the cell junctions, activation of the RHOA pathway (ROCK2 and MYLK), resulting in reorganization of cytoskeletal structure and formation of stress fibers, down regulation of tight and adherens junction proteins, as well as molecular fingerprints of junction destabilization—i.e., phosphorylation of VE-cadherin, down regulation of PTPRB. In these patients, activation of RHO pathway was not limited to the intima but was extended also to the media. ROCK2 and MYLK were more expressed, and their target, MYL9, was both more expressed and more phosphorylated in the media of BAV-ND. Myl6, another light chain of myosin, was also upregulated in BAV-ND, which was further confirmed by proteomic analysis, and MYL6 and MYL9, as well as ROCK2 proteins were upregulated in BAV-D patients. MYLK protein was not detected by proteomics, however, Western blot confirmed the up-regulation of MYLK in BAV-ND.

None of the investigated proteins were differentially expressed in internal thoracic arteries, emphasizing the aortic specificity of the molecular changes. Notably, most markers tested in IHC experiments, including CDH5, CLDN5, PTPRB, ZEB1, ROCK2, and MYLK, which were not differentially expressed at the mRNA level between BAV-ND and TAV-ND, were so in dilated samples.

The initiation and termination of EndMT in atrioventricular canal and OFT cardiac cushions is tightly regulated by oscillation of VEGF signaling^33^,^34^, which is selective for VEGFR1 during the EndMT of OFT^35^. We observed a significant down-regulation of *VEGFR1* and a trend for lower gene expression of other *VEGF* family members in the BAV ascending aorta. VEGF signaling is also known to play a role in migration of NCC^36^. Therefore, it is tempting to speculate that downregulation of VEGF signaling in BAV is associated with the improper termination of cushion EndMT and/or signals related to the migration of NCC. Evidently, the lower VEGF signaling could be the cause or the outcome of improper EndMT/EMT termination. VEGF acts upstream of NOTCH, and through mutual regulation they play a fundamental role in the proper formation of cardiac valves^37^. The two pathways have opposite roles in EndMT of cardiac valves^38^, which is in agreement with our observation that the EndMT signature in BAV is coincident with downregulation of VEGF and upregulation of NOTCH signaling.

NOTCH plays a crucial role in every step of embryonic development of semilunar valves and ascending aorta, including EndMT of the endocardial cushion^39^, and coordination of the interactions between EndMT, SHF progenitors, and migrating NCCs during OFT development^4^.

Here we show the upregulation of NOTCH ligands and receptors in BAV-ND. Previously, we reported upregulation of *NOTCH1* and *NOTCH3* genes in both BAV and TAV patients as a result of dilation, but this increase was more prominent in TAV-D^7^ while our proteomic data showed higher NOTCH3 protein expression in BAV-D patients. This discrepancy implied the regulation of NOTCH by protein modification in BAV. In support of that, MFNG protein and mRNA expression were downregulated in BAV-ND and BAV-D, respectively, consistent with the observation that MFNG is a chaperone negatively regulating NOTCH3 turnover in human cancer cell lines^40^. In addition, upregulation of *MIB1* mRNA in BAV-ND and its upregulation at gene and protein levels in BAV-D implied another level of posttranslational modification of NOTCH in BAV. Accordingly, regulation of NOTCH expression may differ mechanistically, serving different biological functions in the dilation of TAV and BAV aorta.

Based on presented results, we propose that at a critical window of time, a perturbed genetic and/or epigenetic signal may influence the proper communication between endocardium, SHF progenitors, and NCC resulting in the ”incomplete” termination of cardiac cushion EndMT and/or the EMT induced in NCC during delamination and migration into the OFT^11^. The outcome of the impaired cross talk would be a BAV and an improperly differentiated ascending aorta. The activation of RHOA, ZEB1, as well as higher proliferation judged by KI67 staining in the BAV’s aortic media fits well with this hypothesis and is in agreement with the immaturity reported for the SMCs of the ascending aorta of BAV individuals^2^. This perturbation is aortic-specific and does not affect the internal thoracic arteries. Alternatively, the EndMT state and improper differentiation of intima alone may cause an inherent instability of aortic walls. The dysfunctional/leaky endothelium may also give rise to RHOA activation and change of vascular tone in the media via contraction of SMCs. This agrees well with the extensive formation of stress fibers in the endothelium and higher stiffness reported for BAV aorta^41^. Evidently, both mechanisms can simultaneously contribute to the aortic instability in BAV.

In the absence of a representative animal model for human aneurysm associated with BAV, the information gained from patient based studies will be limited, and attempts to understand the detail of the cellular communication described above will largely remain speculative. However, our findings indicate that the NOTCH/VEGF balance may be an important ingredient of the signaling cascade for proper development of aortic valves and maturation of the ascending aorta, the changes of which could underlie the higher susceptibility to aneurysm. Our results further characterize aneurysm formation in BAV as a gradual process and a deepening of an already existing process, explaining the more dramatic changes of TAV relative to BAV upon aneurysm formation, in agreement with our previous report^7^.

Although defective signaling in a number of different pathways could influence the EndMT/EMT during OFT development, the familial inheritance of BAV with a *NOTCH1* mutation, the lack of strong evidence for BAV association with *TGFβ* mutations, and the results described here, collectively imply that a parallel deregulation of the NOTCH/VEGF pathways, rather than a TGFβ-driven process, may be more relevant for the development of aneurysm in BAV, in line with our recently published observations^42^. Indeed, activation of NOTCH upstream of TFGβ has been reported during cardiac development and oncogenic transformation^43^ which share common molecular features with EndMT/EMT.

## Methods

### Clinical samples

Ascending aortic and internal thoracic artery biopsies were obtained from the ASAP Biobank. A detailed description of the study population can be found elsewhere^44^. Briefly, patients underwent elective open-heart surgery at the Karolinska University Hospital, Sweden, for aortic valve and/or ascending aortic disease. Patients were classified based on aortic valve cuspidity and dilatation (aortic diameters of >45 mm and <40 mm were classified as dilated (D) and non-dilated (ND), respectively). Non-dilated patients were operated due to dysfunctional valves. Patients with syndromic aortic pathologies, dissection, and/or significant coronary artery disease were excluded. The study was approved by the Human Research Ethics Committee at Karolinska Institutet (application number 2006/784-31/1), and methods were carried out in accordance with the relevant guidelines. Written informed consent was obtained from all patients according to the Declaration of Helsinki.

Biopsies were taken from the anterior part of the ascending aorta, a few cm above the aortic valve, and from the proximal portion of the internal thoracic artery. The internal thoracic artery was used as a control for exposure to flow disturbances caused by the BAV, as well as for possible genetic alteration that could be traced in vessels other than the aorta. The intima-media of the aortic wall was separated from the adventitia. Due to the small size of non-dilated biopsies, randomly selected specimens were used for proteomic, RNA-analysis, immunohistochemistry, immunofluorescence, electron microscopy, and western blot, from BAV-ND = 62 and TAV-ND = 54. Proteomic on dilated aorta was performed on N = 6 BAV and N = 5 TAV. Characteristics of patients included in proteomic, RNA analysis and immunohistochemistry/fluorescence are shown in Supplementary Table 1a–c.

### HiRIEF and LC-MS/MS of protein samples

Total protein was extracted from the intima-media layer of 21 patients (5 BAV-ND, 5 TAV-ND, 6 BAV-D and 5 TAV-D). We used liquid chromatography–mass spectrometry (LC-MS/MS)-based method with high-resolution isoelectric focusing (HiRIEF) and pre-fractionation at the peptide level, at two different pH ranges, 3.7–4.9 and 4.00–4.25^9^. All MS/MS spectra were searched by Sequest and processed with Percolator under the software platform Proteome Discoverer 1.4 against the human subset of Swissprot, including canonical and isoform entries, version 2015-12 (42080 sequences, from uniprot.org). A cut off of 1% FDR at peptide level was used. One peptide per protein was considered sufficient for protein identification. Quantification was done using Isobaric tags for relative and absolute quantitation (iTRAQ, ABsciex). The 21 samples used for proteomics were arranged in three iTRAQ8plex sets, each set containing 7 samples and one internal standard (prepared by pooling the peptides of all 21 samples), which was used as denominator. iTRAQ reporter ion quantitation and ratio calculations were done by the “Reporter ions Quantifier” module of Proteome Discoverer. The quantitative value of each protein for each patient was calculated as the median of the PSM (peptide spectrum matches) ratios assigned to that particular protein. The obtained ratios were further normalized by median-centering of each sample.

The mass spectrometry proteomics data have been deposited to the ProteomeXchange Consortium via the PRIDE partner repository with the dataset identifier PXD003702 and can be accessed at https://www.ebi.ac.uk/pride/archive/login. Username: reviewer95031@ebi.ac.uk Password: EhAG6nMr).

### Immunohistochemistry (IHC)

Localization and protein expression was studied in aortas and internal thoracic arteries. Deparaffinized sections were treated with or without DIVA solution prior to primary antibodies (Supplementary Table 2).

### Western blot analysis

The expression level of Manic Fringe (MNFG) and myosin light chain kinase (MYLK) were analyzed in protein lysates from non-dilated aortas by Western Blot. Anti-MNFG and anti-MYLK monoclonal antibodies, and horseradish peroxidase–labeled secondary antibodies were used.

### Immunofluorescence

Acetone-fixed cryosections from four BAV and TAV non-dilated aorta were incubated with rabbit anti-VE-cadherin at 4 °C overnight, followed by AlexaFluor 594-labeled goat anti-rabbit IgG for 1 h. Sections were incubated overnight with mouse anti-clathrin, followed by AlexaFluor 488-labeled goat anti-mouse IgG. Nuclei were stained with DAPI. Antibodies specificity was confirmed by incubation with isotype-matched control IgG. Images were obtained using a Zeiss LSM700 confocal laser microscope using the x40 water, 1.2 NA objective lens. Each image consisted of a Z-stack of 15 to 20 optical slices taken at 0.3-μm intervals.

### Transmission electron microscopy (TEM)

Non-dilated and dilated aortic samples from BAV and TAV were fixed in 2% glutaraldehyde + 1% paraformaldehyde. Ultrathin sections (approximately 50–60 nm) were cut by a Leica EM UC 6 and contrasted with uranyl acetate followed by lead citrate and examined in a Tecnai 12 Spirit Bio TWIN transmission electron microscope at 100 kV. Digital images were taken by using a Veleta camera.

### Statistical analysis

Principal component analysis (PCA) and orthogonal projections to latent structures discriminant analysis (OPLS-DA) were used to analyze protein expression (Simca P + 14 × 64 software). Quality parameters for multivariate models are shown in Supplementary Table 3. The significance of quantified proteins was analyzed by means of loadings in the OPLS-DA model, including jack-knife confidence levels derived from cross-validation of the loadings, and calculated as [absolute values of loadings]–[absolute values of jack-knife confidence intervals]. Differences in protein expression for IHC and WB were assessed by Mann-Whitney U-test (GraphPad Prism 5). Differential gene expression has been previously published by us^7^ and here we present the age adjusted data. Briefly, Affymetrix GeneChip Human Exon 1.0 ST arrays were analyzed using student’s t-test, assuming unequal variance. The arrays were validated by qRT-PCR for 11 genes^7^. Data are expressed as mean and percent area positive staining ±SD for gene and protein expression, respectively. P < 0.05 was considered statistically significant. Ingenuity pathway analysis, KEGG pathway and Hallmark analysis (GSEA/MSigDB v5.0)^45^ was used to investigate differentially expressed proteins^45^. These proteins were selected either in non-dilated or dilated samples and were mapped to protein-protein interaction database (Human Protein Reference Database^46^. The retrieved interactions are shown in Fig. 1, and Figs S2 and S3.

## Additional Information

**How to cite this article**: Maleki, S. *et al*. Mesenchymal state of intimal cells may explain higher propensity to ascending aortic aneurysm in bicuspid aortic valves. *Sci. Rep.* **6**, 35712; doi: 10.1038/srep35712 (2016).

## Supplementary Material

Supplementary Information
