# Supplementary material for: Mesenchymal state of intimal cells may explain higher propensity to ascending aortic aneurysm in bicuspid aortic valves
Source: Sci Rep. 2016 Oct 25;6:35712. doi: 10.1038/srep35712 (PMC5078843; doi:10.1038/srep35712)
Supplement: Supplementary Information [file srep35712-s1.pdf]

Supplementary materials

Mesenchymal state of intimal cells may explain higher propensity to ascending aortic aneurysm in bicuspid aortic valves

Shohreh Maleki, Sanela Kjellqvist, Valentina Paloschi, Joelle Magné, Rui Branca, Lei Du, Kjell Hultenby, Johan Petrini, Jonas Fuxe, MIBAVA Leducq Consortium, Janne Lehtiö, Anders Franco-Cereceda, Per Eriksson, Hanna M. Björck.

**Page**	**Content**
2	Detailed Material and Methods
6	Supplementary Table 1A-C, Descriptive patient characteristics
8	Supplementary Table 2, Antibodies
9	Supplementary Table 3, Model parameter values
10	Supplementary Table 4, Significantly up and downregulated proteins, non-dilated and dilated aorta
46	Supplementary Table 5, KEGG and Hallmark analysis, non-dilated and dilated aorta
48	Supplementary Table 6, Ingenuity pathway analysis, non-dilated and dilated aorta
49	Supplementary Table 7, PubMed search for 30 top upregulated proteins in BAV-ND
51	Supplementary Table 8, PubMed search for 30 top downregulated proteins in BAV-ND
53	Supplementary Table 9, mRNA expression, non-dilated and dilated aorta
54	Figure S1, PCA and OPLS-DA of proteomic data
55	Figure S2, Protein–protein interactions (PPIs) network of proteins involved in enriched hallmark gene sets shown in Table S5b and S5d
56	Figure S3, Higher magnification of major pathways selected from Fig 1 and Fig S2
60	Figure S4, EM view of EC junctions in dilated BAV and TAV aorta
61	Figure S5, Staining of neovascularization areas as internal control
62	Figure S6, Relative distribution of PTPRB and CDH5-Y658
63	Figure S7, cell proliferation in relation to cell junction
64	Figure S8, Western blot analysis of MFNG and MYLK expression in BAV and TAV non-dilated aorta
65	Figure S9, Internal thoracic arteries
66	Figure S10, Internal thoracic arteries
67	Figure S11, Internal thoracic arteries
68	Figure S12, Internal thoracic arteries
69	References

Detailed Material and Methods

Clinical samples

Ascending aorta and internal thoracic artery biopsies were obtained from the Advanced Study of Aortic Pathology (ASAP) biobank. A detailed description of the study population can be found elsewhere.[1] In brief, patients undergoing elective open-heart surgery for aortic valve and/or ascending aortic disease at the Karolinska University Hospital, Stockholm, Sweden were included. Patients were classified according to aortic valve cuspidity and dilatation based on visual inspection and transesophagal diameter measurements of the ascending aorta during surgery, respectively. An ascending aortic diameter of >45 mm was considered dilated, ascending aortas with a diameter <40 mm were classified as non-dilated. Non-dilated patients were operated due to dysfunctional valves. Patients with syndromic aortic pathologies, dissection and/or significant coronary artery disease (according to angiography measurements) were excluded from the study. The study was approved by the Human Research Ethics Committee at Karolinska Institutet (application number 2006/784-31/1) and methods were carried out in accordance with the relevant guidelines. Written informed consent was obtained from all patients according to the declaration of Helsinki.

Aortic biopsies were taken from the anterior part of the ascending aorta, a few cm above the aortic valve, and from the proximal portion of the internal thoracic artery. Internal thoracic arteries were used as controls for exposure to flow disturbances caused by the BAV, as well as for possible genetic alteration that could be traced in vessels other than the aorta. The intima-media of the aortic wall was separated from the adventitia. Tissue biopsies for histological studies were incubated in 4% Zn-formaldehyde for 24 hours and stored in ethanol until paraffin embedding. Tissue biopsies for protein expression were snap frozen on dry ice at the site of surgery and stored at -80°C pending protein extraction. Due to the small size of surgically removed non-dilated aorta, non-overlapping and randomly selected specimens were used for proteomic, RNA analysis, immunohistochemistry, immunofluorescence, electron microscopy and western blot from BAV=62 and TAV=54. Proteomic on dilated aorta was done on N=6 BAV and N=5 TAV. Characteristics of patients included in proteomic, RNA analysis and immunohistochemistry/fluorescence are shown in Supplementary Table 1a-c.

HiRiEF and LC-MS/MS of protein samples

Total protein was extracted from the intima-media of 21 patients (5 BAV-ND, 5 TAV-ND, 6 BAV-D and 5 TAV-D), as previously described.[2] The intima-media were dissolved in a cell lysis buffer (2D protein extraction buffer V from GE Healthcare containing 30 mM Tris buffer pH 8.0) including protease inhibitor cocktail (Complete Mini Roche EDTA free). Tissue samples were mechanically homogenized using a TissueLyzer system (Qiagen), according to manufacturer's instructions. Samples were subsequently sonicated in a water bath sonicator at 4°C (30 seconds at high intensity followed by 30 seconds resting for 8 minutes) and pelleted at 12000g for 10 min at 4°C. Protein content was measured by Bradford protein assay (BioRad). Samples were aliquoted and stored at -80°C.

We used a liquid chromatography–mass spectrometry (LC-MS/MS)-based method with high-resolution isoelectric focusing (HiRIEF)[3] and pre-fractionation at the peptide level, at two different pH ranges, 3.7–4.9 and 4.00-4.25. For each LC-MS run of a HiRIEF fraction, the auto sampler (Ultimate 3000 RSLC system, Thermo Scientific Dionex) dispensed 15µl of mobile phase A (97% water, 3% acetonitrile, 0.1% formic acid) into the corresponding well of the microtiter plate (96 well V-bottom, polystyrene, Corning), mixed by aspirating/dispensing 10µl ten times, and finally injected 7µl into a C18 guard desalting column (Acclaim pepmap 100, 75µm x 2cm, nanoViper, Thermo). After 5min of flow at 5µl/min with the loading pump, the 10-port valve switched to analysis mode in which the NC pump provided a flow of 250nL/min through the guard column. The curved gradient (curve 6 in the Chromeleon software) then proceeded from 3% mobile phase B (95% acetonitrile, 5% water, 0.1% formic acid) to 45%B in 45min followed by wash at 99%B and re-equilibration. Total LC-MS run time was 69 min. We used a nano EASY-Spray column (pepmap RSLC, C18, 2µm bead size, 100Å, 75µm internal diameter, 50cm long, Thermo) on the nano electrospray ionization (NSI) EASY-Spray source

(Thermo) at 60°C. Online LC-MS was performed using a hybrid Q-Exactive mass spectrometer (Thermo Scientific). FTMS master scans with 70,000 resolution (and mass range 300-1700 m/z) were followed by data-dependent MS/MS (17,500 resolution) on the top 5 ions using higher energy collision dissociation (HCD) at 30% normalized collision energy. Precursors were isolated with a 2m/z window. Automatic gain control (AGC) targets were 1e6 for MS1 and 1e5 for MS2. Maximum injection times were 100ms for MS1 and 500ms for MS2. Dynamic exclusion was used with 60s duration. Precursors with unassigned charge state or charge state 1 were excluded. An underfill ratio of 1% was used.

All MS/MS spectra were searched by Sequest and processed with Percolator under the software platform Proteome Discoverer 1.4 against the human subset of Swissprot, including canonical and isoform entries, version 2015-12 (42080 sequences, from uniprot.org). Precursor mass tolerance of 10 ppm and product mass tolerance of 0.02 Da were used. The enzyme used was trypsin and up to one missed cleavage was considered. Oxidation of methionine was used as variable modification. Carbamidomethylation of cysteine, and iTRAQ8plex on peptide N-terminus and on lysine were used as fixed modifications. A cut off of 1% FDR at peptide level was used. One peptide per protein was considered sufficient for protein identification. Quantification was done using Isobaric tags for relative and absolute quantitation (iTRAQ, ABsciex). iTRAQ reporter ion quantitation and ratio calculations were done by the "Reporter ions Quantifier" module of Proteome Discoverer. The 21 samples used for proteomics were arranged in three iTRAQ8plex sets, each set containing 7 samples and one internal standard (prepared by pooling the peptides of all 21 samples), which was used as denominator in all ratio calculations. The quantitative value of each protein for each patient was calculated as the median of the PSM (peptide spectrum matches) ratios assigned to that particular protein. The obtained ratios were further normalized by median-centering of each sample.
The mass spectrometry proteomics data have been deposited to the ProteomeXchange Consortium via the PRIDE partner repository with the dataset identifier PXD003702 and can be accessed at https://www.ebi.ac.uk/pride/archive/login. (**Username:** reviewer95031@ebi.ac.uk
Password: EhAG6nMr)

Immunohistochemistry (IHC)
Localization and protein expression levels were tested by immunostaining using deparaffinized tissue sections treated with or without DIVA solution (Biocare Medical), as described previously.[4] Following antigen retrieval, sections were washed and quenched for endogenous peroxidase activity with 3% hydrogen peroxide for 5 minutes. Nonspecific binding sites were blocked with 20% normal goat or horse serum for 30 minutes and then incubated overnight at 4°C with primary antibody (Supplementary Table 2). After washing, sections were incubated with biotinylated anti-rabbit or anti-goat IgG (Vector Laboratories) for 1 hour. Sections were then incubated with Avidin-biotin peroxidase complex (Vectastain ABC kit, Vector Laboratories) for 30 min in room temperature, followed by visualization by 3,3’-diaminobenzidine tetrahydrochloride (Dako). All sections were counterstained with Mayer’s hematoxylin (Histolab Products). Quantification of percent area positive staining was performed using Image-Pro® Premier version 9.1 Software (Media Cybernetics®, Silver Spring, MD). The expression of each protein was studied in 6-10 patients from each group (BAV-ND and TAV-ND) in both aortas and internal thoracic arteries (unless otherwise stated).

Western Blot analysis
The expression level of Manic Fringe (MNFG) and MYLK (myosin light chain kinase) were analyzed by Western Blot. Protein lysates were prepared by tissue homogenization in RIPA buffer (Thermo Fisher Scientific) as described in the proteomic method section for non-dilated aorta of BAV and TAV patients. Twenty microgram of protein was re-suspended in Laemmli Sample Buffer (BioRad) containing 0.2M DTT and subjected to SDS-PAGE using 4-15% Bis-Tris Protein Gels (Invitrogen NuPAGE), or 4-20% Mini-PROTEAN TGX gels (BioRad) for 60 min at 120V. Proteins transfer was performed with Tran-Blot turbo system (Bio Rad) to nitrocellulose membranes (Bio Rad) according to standard protocols, followed by blocking in 5% dry-milk in TTBS. The blots were incubated with

monoclonal primary anti-MNFG or anti-MYLK antibodies overnight, followed by 30 minutes incubation with horseradish peroxidase–labeled secondary antibody. Enhanced chemiluminescence Western blot detection reagent (Amersham, GE Healthcare) was used for detection. Monoclonal anti-beta-actin antibody was used as loading control (Sigma).

Immunofluorescence

Acetone-fixed cryosections of four BAV and TAV non-dilated aorta were first incubated with rabbit anti-VE-Cadherin antibody or rabbit anti-Von Willebrand Factor antibody (Supplementary Table 2) at 4°C overnight, followed by AlexaFluor 594 labeled goat anti-rabbit IgG (Life technologies, Stockholm, Sweden) for 1h. Sections were then incubated overnight with mouse anti-clathrin antibody (Ab2731, Abcam, Stockholm, Sweden), followed by AlexaFluor 488 labeled goat anti-mouse IgG (Life technologies, Stockholm, Sweden), with subsequent staining of the nuclei using DAPI. Antibody specificity was confirmed by incubation with isotype-matched control IgG. Images were obtained using a Zeiss LSM700 confocal laser microscope using x40 water, 1.2 of numerical aperture (NA) objective lens. Each image consisted of a Z-stack of 15 to 20 optical slices taken at 0.3µm intervals.

Transmission electron microscopy (TEM)

TEM was done as described previously.[5] Briefly, samples of ascending aortas from three BAV and TAV non-dilated individuals were dissected and small pieces were fixed in 2% glutaraldehyde +1% paraformaldehyde in 0.1M phosphate buffer, pH 7.4 at room temperature and stored at 4°C. Specimens were rinsed in 0.1 M phosphate buffer, pH 7.4 and post-fixed in 2% osmium tetroxide 0.1 M phosphate buffer, pH 7.4 at 4oC for 2 hours, dehydrated in ethanol followed by acetone and embedded in LX-112 (Ladd, Burlington, Vermont, USA). Semi-thin sections were cut and stained with toluidine blue O and used for light microscopic analysis. Ultrathin sections (approximately 50-60 nm) were cut by a Leica EM UC 6 (Leica, Wien, Austria) and contrasted with uranyl acetate followed by lead citrate and examined in a Tecnai 12 Spirit Bio TWIN transmission electron microscope (FEI company, Eindhoven, The Netherlands) at 100 kV. Digital images were taken by using a Veleta camera (Olympus Soft Imaging Solutions, GmbH, Münster, Germany).[6]

Statistical analysis

Principal component analysis (PCA)[7], and orthogonal projections to latent structures discriminant analysis (OPLS-DA) were used to analyze proteomics data. OPLS-DA is a supervised multivariate analysis method where the systematic variation in X matrix is divided into two parts, one that is linearly related to Y (and therefore predictive) and one that is unrelated (orthogonal) to Y[8,9]. Two new units from two types of analyses (PCA and OPLS-DA), scores and loadings can be obtained. The scores give information about the new observations projected onto a lower dimensional plane that summarizes the behavior of the original ones. The loadings reveal how the PC model plane is inserted into the variable space and thereby can be used to interpret the meaning of the scores. Quality parameters for all multivariate analysis models performed are shown in Supplementary Table 3. The protein expressions were subjected to UV scaling and mean centering prior to multivariate data analysis. The PCA and OPLS-DA analyses were performed using Simca P+13.0.2x64 (Umetrics) software. The multivariate models were mean centered and unit variance scaled and model complexity was estimated according to 7-fold cross-validation[10] in Simca P+14x64 (Umetrics) software, unless otherwise stated.

PCA analysis was carried out using color coding of TAV and BAV samples. The significance of differentially expressed proteins was analyzed by means of loadings in OPLS-DA model (with BAV samples as a Y vector), including jack-knife confidence levels derived from cross-validation of the loadings.[9,10] Significance was calculated as absolute values of loadings – absolute values of jack-knife confidence intervals; if positive indicates significant protein. Differential gene expression has been previously published by us[11] and here we present the age adjusted data. Briefly, RNA was extracted from intima-media biopsies of both non-dilated and dilated aorta and analyzed by Affymetrix GeneChip Human Exon 1.0 ST arrays, using Student's T-test assuming unequal variance (R 2.13.0).

The array data was validated by qRT-PCR for 11 genes.[11] Differences in protein expression assessed by IHC and WB were analyzed by student's t-test (GraphPad Prism 5, La Jolla, CA). Data are expressed as mean and percent area positive staining ± SD for gene and protein expression, respectively. A P-value of $P<0.05$ was considered statistically significant. Ingenuity pathway analysis was performed on significant proteins from the OPLS-DA model. Canonical pathways are shown. KEGG pathway and Hallmark analysis within Gene Set Enrichment Analysis and the Molecular Signatures Database resource v 5.0 was used to investigate gene/protein sets.[12] Differentially expressed proteins were selected either in non-dilated or dilated samples and were mapped to protein-protein interaction database (Human Protein Reference Database.[13] The retrieved interactions are shown in Fig 1, and Figs S2 and S3.

Supplementary Table 1a-c. Descriptive patient characteristics

Parameter[1]	**BAV Non-dilated**	**TAV Non-dilated**	**P-value**	**BAV Dilated**	**TAV Dilated**	**P-value**
**N**	5	5		6	5	
**Age, years**	60 (7)	69 (8)	0.08	54 (9)	70 (7)	<0.05
**Female gender**	1 (20)	1 (20)	NS	1 (17)	2 (40)	0.51
**Smoking, current smokers**	1 (20)	0 (0)	0.29	0 (0)	0 (0)	NS
**BMI, Kg/m^2**	28.2 (1.7)	31.5 (6.7)	0.25	26.8 (3.5)	26.6 (4.5)	0.86
**Triglycerides, mmo/L**	1.36 (0.31)	1.74 (1.00)	0.25	1.46 (0.88)	1.05 (0.39)	0.52
**Cholesterol, mmo/L**	5.34 (1.14)	4.98 (0.64)	0.53	4.97 (0.79)	4.50 (1.13)	0.27
**hsCRP, mg/L**	0.68 (0.44)	1.95 (1.36)	0.08	1.63 (1.41)	33.10 (38.27)	<0.05
**Aortic aneurysm and valve disease**						
**Max ascending aortic diameter, mm**	34.0 (1.9)	31.6 (3.7)	0.35	53.1 (7.9)	56.7 (6.2)	0.27
**Aortic valve stenosis**	4 (80)	4 (80)	NS	3 (50)	0 (0)	0.06
**Aortic valve regurgitation**	1 (20)	1 (20)	NS	3 (50)	4 (80)	0.30
**Medication**						
**Beta blockers**	1 (20)	3 (60)	0.20	2 (33)	3 (60)	0.38
**Ca antagonists**	1 (20)	2 (40)	0.49	0 (0)	1 (20)	0.25
**ACE inhibitors**	1 (20)	1 (20)	NS	0 (0)	1 (20)	0.25
**Statins**	0 (0)	3 (60)	<0.05	1 (17)	1 (20)	0.89

**Parameter[1]**	**BAV Non-dilated**	**TAV Non-dilated**	**P-value**
**N**	15	15	
**Age, years**	52 (13)	75 (6)	<0.0001
**Female gender**	4 (27)	6 (40)	0.44
**Smoking, current smokers**	2 (13)	0 (0)	0.14
**BMI, Kg/m^2**	25.3 (4.4)	26.1 (3.5)	0.82
**Triglycerides, mmo/L**	1.39 (1.50)	1.18 (0.62)	0.87
**Cholesterol, mmo/L**	5.41 (1.21)	4.61 (0.91)	<0.05
**hsCRP, mg/L**	5.84 (15.08)	2.13 (1.92)	0.28
**Aortic aneurysm and valve disease**			
**Max ascending aortic diameter, mm**	33.4 (4.8)	30.7 (4.3)	0.10
**Aortic valve stenosis**	11 (73)	14 (93)	0.14
**Aortic valve regurgitation**	4 (27)	1 (7)	0.14
**Medication**			
**Beta blockers**	2 (13)	9 (60)	<0.01
**Ca antagonists**	1 (7)	5 (33)	0.07
**ACE inhibitors**	6 (40)	6 (40)	NS
**Statins**	4 (27)	11 (73)	<0.05

Parameters	BAV Non-dilated	TAV Non-dilated	P-value	BAV Dilated	TAV Dilated	P-value
**N**	31	23		44	21	
**Age, years**	58 (10)	67 (12)	<0.01	61 (11)	63 (14)	0.30
**Female gender**	6 (19)	6 (26)	0.56	11 (25)	9 (43)	0.14
**Smoking, current smokers**	4 (13)	2 (9)	0.63	5 (11)	2 (10)	0.82
**BMI, Kg/m^2**	26.7 (5.4)	26.8 (4.9)	0.91	26.2 (3.6)	27.1 (3.9)	0.51
**Triglycerides, mmo/L**	1.28 (0.55)	1.03 (0.41)	0.06	1.56 (2.00)	1.19 (0.65)	0.62
**Cholesterol, mmo/L**	5.07 (1.10)	5.33 (0.85)	0.16	5.08 (1.29)	4.83 (1.06)	0.42
**hsCRP, mg/L**	2.58 (3.89)	4.04 (4.08)	0.06	2.22 (2.80)	6.77 (16.89)	<0.05
**Aortic aneurysm and valve disease**						
**Max ascending aortic diameter, mm**	35.4 (3.4)	32.1 (4.3)	<0.01	50.1 (3.2)	54.9 (8.1)	0.06
**Aortic valve stenosis**	19 (61)	13 (57)	0.72	30 (68)	1 (5)	<0.0001
**Aortic valve regurgitation**	11 (35)	10 (43)	0.55	11 (25)	15 (71)	<0.001
**Medication**						
**Beta blockers**	13 (42)	10 (43)	0.91	16 (36)	10 (48)	0.39
**Ca antagonists**	4 (13)	0 (0)	0.07	4 (9)	2 (10)	0.96
**ACE inhibitors**	6 (19)	5 (22)	0.83	11 (25)	6 (29)	0.76
**Statins**	8 (26)	2 (9)	0.11	18 (41)	6 (29)	0.34

BMI, body mass index; hsCRP, high-sensitive C-reactive protein; ACE, Angiotensin-converting enzyme. [1]All continuous variables are presented as mean (SD) and ordinal variables are presented as n (%). P values indicate significance for differences between the two groups using a Mann-Whitney test for continuous variables and a Chi-square test for nominal variables. Non-dilated, aorta <40mm; Dilated, aorta >45mm; NS, not significant.

Supplementary Table 2. Antibodies

CLDN5	(sc-17667, Santa Cruz Biotechnology), without DIVA treatment
CDH5	(ab33168, Abcam)
CDH5-Phospho-Y658	(ab27775, Abcam)
CLTC (Clathrin)	(Ab2731, Abcam)
PTPRB	(sc-1114, Santa Cruz Biotechnology), without DIVA treatment
ROCK2	(sc-1851, Santa Cruz Biotechnology)
MYLK	(sc-25428, Santa Cruz Biotechnology; H00004638-M01, Abnova)
MYL9	(ab64161, Abcam)
MYL9-Phospho-S19	(ab64162, Abcam)
MYL6	(sc-109585, Santa Cruz Biotechnology)
MNFG	(H00004242-M07, Abnova)
KI67	(ab 16667, Abcam), without DIVA treatment
ZEB1	(ab87280, Abcam)
Activated NOTCH1	(ab8925, Abcam)
NOTCH3	(sc-5593, Santa Cruz Biotechnology)
NOTCH4	(sc-8644, Santa Cruz Biotechnology)
JAG1	(sc-6011, Santa Cruz Biotechnology)
JAG2	(sc-5604, Santa Cruz Biotechnology)
DLL4	(sc-18640, Santa Cruz Biotechnology)
vWF	(A0082, DakoCytomation)

Supplementary Table 3. Model parameter values

Model quality parameters of PCA and OPLS-DA. No of PCs refers to the optimal number of PCs for a particular multivariate data model according to cross-validation. R_X^2 accounts for the explained variance while Q_Y^2 accounts for the cumulative fraction of the total variation of Y that can be predicted by the model. Q_X^2 accounts for the cumulative fraction of the total variation of X that can be predicted by the model. R_Y^2 accounts for the explained variance for the Y component.

PCA model

Model description	No of PCs	No of samples	No of proteins	R_X^2	Q_X^2
Non-dilated patients only included - **Supp Figure 1a**	2	10	2894	0,42	0,04
Dilated patients only included - **Supp Figure 1c**	2	11	2894	0,5	0,2

OPLS-DA model

Model description	No of PCs	No of samples	No of proteins	R_Y^2	Q_Y^2	R_X^2
Non-dilated patients only included, TAV vs BAV discriminant analysis - **Supp Figure 1b**	1+3	10	2894	1	0,52	0,6
Dilated patients only included TAV vs BAV discriminant analysis - **Supp Figure 1d**	1+4	11	2894	1	0,88	0,75

Supplementary Table 4a. Loadings and jack-knife (JK) confidence intervals for significant proteins in non-dilated samples, sorted according to loading value. OPLS-DA model is based on TAV and BAV being added as a Y variable. The significance was calculated as ABS (loading) – ABS (jack-knife confidence interval); the positive values indicated significantly expressed proteins.

	Proteins upregulated in BAV non-dilated samples				
Protein accession number	Protein name	Loading value [a]	Fold change	jack-knife (JK) confidence interval[b]	ABS (Loading value)-ABS(JK)[c]
Q96RK0	Protein capicua homolog OS=Homo sapiens GN=CIC PE=1 SV=2 - [CIC_HUMAN]	0,046	1,25	0,031	0,015
P98170	E3 ubiquitin-protein ligase XIAP OS=Homo sapiens GN=XIAP PE=1 SV=2 - [XIAP_HUMAN]	0,046	1,18	0,022	0,024
P46527	Cyclin-dependent kinase inhibitor 1B OS=Homo sapiens GN=CDKN1B PE=1 SV=1 - [CDN1B_HUMAN]	0,045	1,59	0,016	0,029
Q9Y6K1-3	Isoform 3 of DNA (cytosine-5)-methyltransferase 3A OS=Homo sapiens GN=DNMT3A - [DNM3A_HUMAN]	0,044	1,53	0,031	0,013
Q15293	Reticulocalbin-1 OS=Homo sapiens GN=RCN1 PE=1 SV=1 - [RCN1_HUMAN]	0,043	1,20	0,022	0,021
P60900-2	Isoform 2 of Proteasome subunit alpha type-6 OS=Homo sapiens GN=PSMA6 - [PSA6_HUMAN]	0,043	1,21	0,010	0,033
O14964-2	Isoform 2 of Hepatocyte growth factor-regulated tyrosine kinase substrate OS=Homo sapiens GN=HGS - [HGS_HUMAN]	0,043	1,30	0,029	0,014
Q6UX71	Plexin domain-containing protein 2 OS=Homo sapiens GN=PLXDC2 PE=1 SV=1 - [PXDC2_HUMAN]	0,043	1,28	0,031	0,012
P0CG38	POTE ankyrin domain family member I OS=Homo sapiens GN=POTEI PE=3 SV=1 - [POTEI_HUMAN]	0,043	1,45	0,021	0,022
Q96FW1	Ubiquitin thioesterase OTUB1 OS=Homo sapiens GN=OTUB1 PE=1 SV=2 - [OTUB1_HUMAN]	0,043	1,26	0,021	0,022
P08579	U2 small nuclear ribonucleoprotein B'' OS=Homo sapiens GN=SNRPB2 PE=1 SV=1 - [RU2B_HUMAN]	0,042	1,31	0,027	0,016
Q9NSE4	Isoleucine--tRNA ligase, mitochondrial OS=Homo sapiens GN=IARS2 PE=1 SV=2 - [SYIM_HUMAN]	0,042	1,16	0,022	0,020
P80404	4-aminobutyrate aminotransferase, mitochondrial OS=Homo sapiens GN=ABAT PE=1 SV=3 - [GABT_HUMAN]	0,042	1,21	0,036	0,005
Q969E4	Transcription elongation factor A protein-like 3 OS=Homo sapiens GN=TCEAL3 PE=1 SV=1 - [TCAL3_HUMAN]	0,041	1,21	0,029	0,012
Q9UKZ1	CCR4-NOT transcription complex subunit 11 OS=Homo sapiens GN=CNOT11 PE=1 SV=1 - [CNO11_HUMAN]	0,041	1,25	0,024	0,017
P51689-2	Isoform 2 of Arylsulfatase D OS=Homo sapiens GN=ARSD - [ARSD_HUMAN]	0,041	1,15	0,040	0,002
P13995-2	Isoform 2 of Bifunctional methylenetetrahydrofolate dehydrogenase/cyclohydrolase, mitochondrial OS=Homo sapiens GN=MTHFD2 - [MTDC_HUMAN]	0,041	1,63	0,028	0,013
Q15942	Zyxin OS=Homo sapiens GN=ZYX PE=1 SV=1 - [ZYX_HUMAN]	0,041	1,37	0,026	0,015
Q14005-4	Isoform 4 of Pro-interleukin-16 OS=Homo sapiens GN=IL16 - [IL16_HUMAN]	0,041	1,24	0,027	0,014
Q86YS7-2	Isoform 2 of C2 domain-containing protein 5 OS=Homo sapiens GN=C2CD5 - [C2CD5_HUMAN]	0,041	1,11	0,035	0,006
Q14108	Lysosome membrane protein 2 OS=Homo sapiens GN=SCARB2 PE=1 SV=2 - [SCRB2_HUMAN]	0,040	1,24	0,020	0,021
Q8IVN8	Somatomedin-B and thrombospondin type-1 domain-containing protein OS=Homo sapiens GN=SBSPON PE=1 SV=2 - [SBSPO_HUMAN]	0,040	2,11	0,030	0,010

P48163	NADP-dependent malic enzyme OS=Homo sapiens GN=ME1 PE=1 SV=1 - [MAOX_HUMAN]	0,040	1,20	0,017	0,023
P46937-5	Isoform 5 of Transcriptional coactivator YAP1 OS=Homo sapiens GN=YAP1 - [YAP1_HUMAN]	0,040	1,23	0,021	0,019
Q01105-3	Isoform 3 of Protein SET OS=Homo sapiens GN=SET - [SET_HUMAN]	0,040	1,13	0,036	0,004
Q9NVG8	TBC1 domain family member 13 OS=Homo sapiens GN=TBC1D13 PE=1 SV=3 - [TBC13_HUMAN]	0,040	1,14	0,030	0,010
Q9NUJ1	Mycophenolic acid acyl-glucuronide esterase, mitochondrial OS=Homo sapiens GN=ABHD10 PE=1 SV=1 - [ABHDA_HUMAN]	0,040	1,42	0,015	0,025
Q9BRG1	Vacuolar protein-sorting-associated protein 25 OS=Homo sapiens GN=VPS25 PE=1 SV=1 - [VPS25_HUMAN]	0,040	1,34	0,031	0,008
P10515	Dihydrolipoyllysine-residue acetyltransferase component of pyruvate dehydrogenase complex, mitochondrial OS=Homo sapiens GN=DLAT PE=1 SV=3 - [ODP2_HUMAN]	0,040	1,32	0,031	0,009
Q15120	[Pyruvate dehydrogenase (acetyl-transferring)] kinase isozyme 3, mitochondrial OS=Homo sapiens GN=PDK3 PE=1 SV=1 - [PDK3_HUMAN]	0,039	1,15	0,024	0,015
Q9UK41	Vacuolar protein sorting-associated protein 28 homolog OS=Homo sapiens GN=VPS28 PE=1 SV=1 - [VPS28_HUMAN]	0,039	1,50	0,035	0,004
P35232	Prohibitin OS=Homo sapiens GN=PHB PE=1 SV=1 - [PHB_HUMAN]	0,039	1,21	0,033	0,007
P05166	Propionyl-CoA carboxylase beta chain, mitochondrial OS=Homo sapiens GN=PCCB PE=1 SV=3 - [PCCB_HUMAN]	0,039	1,09	0,029	0,010
Q63ZY3-3	Isoform 3 of KN motif and ankyrin repeat domain-containing protein 2 OS=Homo sapiens GN=KANK2 - [KANK2_HUMAN]	0,039	1,17	0,012	0,027
Q9UBQ0	Vacuolar protein sorting-associated protein 29 OS=Homo sapiens GN=VPS29 PE=1 SV=1 - [VPS29_HUMAN]	0,039	1,47	0,023	0,016
Q8TF09	Dynein light chain roadblock-type 2 OS=Homo sapiens GN=DYNLRB2 PE=1 SV=1 - [DLRB2_HUMAN]	0,038	1,50	0,015	0,024
Q8NE01-2	Isoform 2 of Metal transporter CNNM3 OS=Homo sapiens GN=CNNM3 - [CNNM3_HUMAN]	0,038	1,22	0,032	0,007
O95208-2	Isoform 2 of Epsin-2 OS=Homo sapiens GN=EPN2 - [EPN2_HUMAN]	0,038	1,23	0,037	0,001
O75083	WD repeat-containing protein 1 OS=Homo sapiens GN=WDR1 PE=1 SV=4 - [WDR1_HUMAN]	0,038	1,25	0,025	0,013
P36957	Dihydrolipoyllysine-residue succinyltransferase component of 2-oxoglutarate dehydrogenase complex, mitochondrial OS=Homo sapiens GN=DLST PE=1 SV=4 - [ODO2_HUMAN]	0,038	1,21	0,027	0,011
O14907	Tax1-binding protein 3 OS=Homo sapiens GN=TAX1BP3 PE=1 SV=2 - [TX1B3_HUMAN]	0,038	1,26	0,027	0,011
P0CG39	POTE ankyrin domain family member J OS=Homo sapiens GN=POTEJ PE=3 SV=1 - [POTEJ_HUMAN]	0,038	1,36	0,026	0,012
P61020	Ras-related protein Rab-5B OS=Homo sapiens GN=RAB5B PE=1 SV=1 - [RAB5B_HUMAN]	0,038	1,16	0,025	0,013
Q15311	RalA-binding protein 1 OS=Homo sapiens GN=RALBP1 PE=1 SV=3 - [RBP1_HUMAN]	0,038	1,29	0,032	0,006
Q96RQ3	Methylcrotonoyl-CoA carboxylase subunit alpha, mitochondrial OS=Homo sapiens GN=MCCC1 PE=1 SV=3 - [MCCA_HUMAN]	0,037	1,19	0,034	0,004
Q4G0N4-2	Isoform 2 of NAD kinase 2, mitochondrial OS=Homo sapiens GN=NADK2 - [NAKD2_HUMAN]	0,037	1,13	0,030	0,007
Q8TC07-2	Isoform 2 of TBC1 domain family member 15 OS=Homo sapiens GN=TBC1D15 - [TBC15_HUMAN]	0,037	1,34	0,032	0,005
P49755	Transmembrane emp24 domain-containing protein 10 OS=Homo sapiens GN=TMED10 PE=1 SV=2 - [TMEDA_HUMAN]	0,037	1,14	0,033	0,004
P30047	GTP cyclohydrolase 1 feedback regulatory protein OS=Homo sapiens GN=GCHFR PE=1 SV=3 - [GFRP_HUMAN]	0,037	1,18	0,033	0,004
P55786	Puromycin-sensitive aminopeptidase OS=Homo sapiens GN=NPEPPS PE=1 SV=2 - [PSA_HUMAN]	0,037	1,12	0,035	0,002
P42226-2	Isoform 2 of Signal transducer and activator of transcription 6 OS=Homo sapiens GN=STAT6 - [STAT6_HUMAN]	0,037	1,80	0,035	0,002

O00291-3	Isoform 3 of Huntingtin-interacting protein 1 OS=Homo sapiens GN=HIP1 - [HIP1_HUMAN]	0,037	1,18	0,035	0,002
Q9BY43	Charged multivesicular body protein 4a OS=Homo sapiens GN=CHMP4A PE=1 SV=3 - [CHM4A_HUMAN]	0,036	1,27	0,031	0,005
Q6YHK3-4	Isoform 4 of CD109 antigen OS=Homo sapiens GN=CD109 - [CD109_HUMAN]	0,036	1,20	0,032	0,005
Q99714-2	Isoform 2 of 3-hydroxyacyl-CoA dehydrogenase type-2 OS=Homo sapiens GN=HSD17B10 - [HCD2_HUMAN]	0,036	1,14	0,027	0,009
O95999	B-cell lymphoma/leukemia 10 OS=Homo sapiens GN=BCL10 PE=1 SV=1 - [BCL10_HUMAN]	0,036	1,23	0,027	0,009
Q15555-4	Isoform 4 of Microtubule-associated protein RP/EB family member 2 OS=Homo sapiens GN=MAPRE2 - [MARE2_HUMAN]	0,036	1,12	0,031	0,005
Q05209-2	Isoform 2 of Tyrosine-protein phosphatase non-receptor type 12 OS=Homo sapiens GN=PTPN12 - [PTN12_HUMAN]	0,036	1,22	0,033	0,003
P46926	Glucosamine-6-phosphate isomerase 1 OS=Homo sapiens GN=GNPDA1 PE=1 SV=1 - [GNPI1_HUMAN]	0,036	1,43	0,025	0,011
Q15165-3	Isoform 3 of Serum paraoxonase/arylesterase 2 OS=Homo sapiens GN=PON2 - [PON2_HUMAN]	0,036	1,29	0,026	0,010
P14649	Myosin light chain 6B OS=Homo sapiens GN=MYL6B PE=1 SV=1 - [MYL6B_HUMAN]	0,035	1,12	0,034	0,002
Q8NBJ7-2	Isoform 2 of Sulfatase-modifying factor 2 OS=Homo sapiens GN=SUMF2 - [SUMF2_HUMAN]	0,035	1,28	0,033	0,003
P00367	Glutamate dehydrogenase 1, mitochondrial OS=Homo sapiens GN=GLUD1 PE=1 SV=2 - [DHE3_HUMAN]	0,035	1,22	0,023	0,012
Q96IZ0	PRKC apoptosis WT1 regulator protein OS=Homo sapiens GN=PAWR PE=1 SV=1 - [PAWR_HUMAN]	0,035	1,13	0,022	0,013
Q9H8Y8-2	Isoform 2 of Golgi reassembly-stacking protein 2 OS=Homo sapiens GN=GORASP2 - [GORS2_HUMAN]	0,035	1,26	0,033	0,002
Q9NR99	Matrix-remodeling-associated protein 5 OS=Homo sapiens GN=MXRA5 PE=2 SV=3 - [MXRA5_HUMAN]	0,035	1,44	0,029	0,006
Q00796	Sorbitol dehydrogenase OS=Homo sapiens GN=SORD PE=1 SV=4 - [DHSO_HUMAN]	0,035	1,17	0,033	0,002
P67775-2	Isoform 2 of Serine/threonine-protein phosphatase 2A catalytic subunit alpha isoform OS=Homo sapiens GN=PPP2CA - [PP2AA_HUMAN]	0,035	1,26	0,032	0,003
P26440-2	Isoform 2 of Isovaleryl-CoA dehydrogenase, mitochondrial OS=Homo sapiens GN=IVD - [IVD_HUMAN]	0,035	1,38	0,021	0,013
Q9HAU0-5	Isoform 5 of Pleckstrin homology domain-containing family A member 5 OS=Homo sapiens GN=PLEKHA5 - [PKHA5_HUMAN]	0,034	1,13	0,031	0,003
Q16891-2	Isoform 2 of MICOS complex subunit MIC60 OS=Homo sapiens GN=IMMT - [MIC60_HUMAN]	0,034	1,17	0,029	0,005
O75436	Vacuolar protein sorting-associated protein 26A OS=Homo sapiens GN=VPS26A PE=1 SV=2 - [VP26A_HUMAN]	0,034	1,08	0,021	0,013
P45880-2	Isoform 2 of Voltage-dependent anion-selective channel protein 2 OS=Homo sapiens GN=VDAC2 - [VDAC2_HUMAN]	0,034	1,27	0,034	0,000
P09622-2	Isoform 2 of Dihydrolipoyl dehydrogenase, mitochondrial OS=Homo sapiens GN=DLD - [DLDH_HUMAN]	0,034	1,17	0,020	0,014
P24043	Laminin subunit alpha-2 OS=Homo sapiens GN=LAMA2 PE=1 SV=4 - [LAMA2_HUMAN]	0,034	1,56	0,031	0,003
Q96KA5-2	Isoform 2 of Cleft lip and palate transmembrane protein 1-like protein OS=Homo sapiens GN=CLPTM1L - [CLP1L_HUMAN]	0,034	1,18	0,033	0,001
Q92843-2	Isoform 3 of Bcl-2-like protein 2 OS=Homo sapiens GN=BCL2L2 - [B2CL2_HUMAN]	0,034	1,13	0,024	0,010
Q96GX2	Putative ataxin-7-like protein 3B OS=Homo sapiens GN=ATXN7L3B PE=3 SV=2 - [A7L3B_HUMAN]	0,033	1,16	0,027	0,007
P12111	Collagen alpha-3(VI) chain OS=Homo sapiens GN=COL6A3 PE=1 SV=5 - [CO6A3_HUMAN]	0,033	1,34	0,031	0,003

Q14249	Endonuclease G, mitochondrial OS=Homo sapiens GN=ENDOG PE=1 SV=4 - [NUCG_HUMAN]	0,033	1,63	0,027	0,006
P18669	Phosphoglycerate mutase 1 OS=Homo sapiens GN=PGAM1 PE=1 SV=2 - [PGAM1_HUMAN]	0,033	1,21	0,025	0,008
P07902	Galactose-1-phosphate uridylyltransferase OS=Homo sapiens GN=GALT PE=1 SV=3 - [GALT_HUMAN]	0,033	1,24	0,031	0,002
P29992	Guanine nucleotide-binding protein subunit alpha-11 OS=Homo sapiens GN=GNA11 PE=1 SV=2 - [GNA11_HUMAN]	0,033	1,44	0,021	0,012
Q9Y2H6-2	Isoform 2 of Fibronectin type-III domain-containing protein 3A OS=Homo sapiens GN=FNDC3A - [FND3A_HUMAN]	0,033	1,19	0,025	0,008
O00330-2	Isoform 2 of Pyruvate dehydrogenase protein X component, mitochondrial OS=Homo sapiens GN=PDHX - [ODPX_HUMAN]	0,033	1,20	0,025	0,008
P28300	Protein-lysine 6-oxidase OS=Homo sapiens GN=LOX PE=1 SV=2 - [LYOX_HUMAN]	0,033	1,93	0,030	0,003
P54922	[Protein ADP-ribosylarginine] hydrolase OS=Homo sapiens GN=ADPRH PE=1 SV=1 - [ADPRH_HUMAN]	0,033	1,23	0,032	0,001
Q96C86	m7GpppX diphosphatase OS=Homo sapiens GN=DCPS PE=1 SV=2 - [DCPS_HUMAN]	0,033	1,14	0,030	0,003
O95967	EGF-containing fibulin-like extracellular matrix protein 2 OS=Homo sapiens GN=EFEMP2 PE=1 SV=3 - [FBLN4_HUMAN]	0,033	1,44	0,029	0,003
Q9GZU7-2	Isoform 2 of Carboxy-terminal domain RNA polymerase II polypeptide A small phosphatase 1 OS=Homo sapiens GN=CTDSP1 - [CTDS1_HUMAN]	0,032	1,12	0,030	0,002
P63151	Serine/threonine-protein phosphatase 2A 55 kDa regulatory subunit B alpha isoform OS=Homo sapiens GN=PPP2R2A PE=1 SV=1 - [2ABA_HUMAN]	0,032	1,38	0,027	0,005
O00764-2	Isoform 2 of Pyridoxal kinase OS=Homo sapiens GN=PDXK - [PDXK_HUMAN]	0,032	1,29	0,028	0,004
Q9H444	Charged multivesicular body protein 4b OS=Homo sapiens GN=CHMP4B PE=1 SV=1 - [CHM4B_HUMAN]	0,032	1,26	0,023	0,009
P25942	Tumor necrosis factor receptor superfamily member 5 OS=Homo sapiens GN=CD40 PE=1 SV=1 - [TNR5_HUMAN]	0,032	1,23	0,030	0,002
Q9UJU6	Drebrin-like protein OS=Homo sapiens GN=DBNL PE=1 SV=1 - [DBNL_HUMAN]	0,032	1,39	0,019	0,012
P46109	Crk-like protein OS=Homo sapiens GN=CRKL PE=1 SV=1 - [CRKL_HUMAN]	0,032	1,23	0,030	0,001
A6NIH7	Protein unc-119 homolog B OS=Homo sapiens GN=UNC119B PE=1 SV=1 - [U119B_HUMAN]	0,032	1,26	0,023	0,009
Q9P0L0	Vesicle-associated membrane protein-associated protein A OS=Homo sapiens GN=VAPA PE=1 SV=3 - [VAPA_HUMAN]	0,031	1,13	0,029	0,002
P49593	Protein phosphatase 1F OS=Homo sapiens GN=PPM1F PE=1 SV=3 - [PPM1F_HUMAN]	0,031	1,15	0,022	0,009
P62070-3	Isoform 3 of Ras-related protein R-Ras2 OS=Homo sapiens GN=RRAS2 - [RRAS2_HUMAN]	0,031	1,46	0,024	0,007
Q9UHG3	Prenylcysteine oxidase 1 OS=Homo sapiens GN=PCYOX1 PE=1 SV=3 - [PCYOX_HUMAN]	0,031	1,15	0,031	0,000
Q92542-2	Isoform 2 of Nicastrin OS=Homo sapiens GN=NCSTN - [NICA_HUMAN]	0,031	1,32	0,027	0,004
Q03135	Caveolin-1 OS=Homo sapiens GN=CAV1 PE=1 SV=4 - [CAV1_HUMAN]	0,031	1,11	0,028	0,003
P61158	Actin-related protein 3 OS=Homo sapiens GN=ACTR3 PE=1 SV=3 - [ARP3_HUMAN]	0,031	1,08	0,027	0,004
Q9ULC3	Ras-related protein Rab-23 OS=Homo sapiens GN=RAB23 PE=1 SV=1 - [RAB23_HUMAN]	0,031	1,21	0,019	0,011
O00410	Importin-5 OS=Homo sapiens GN=IPO5 PE=1 SV=4 - [IPO5_HUMAN]	0,030	1,23	0,026	0,004
Q08379	Golgin subfamily A member 2 OS=Homo sapiens GN=GOLGA2 PE=1 SV=3 - [GOGA2_HUMAN]	0,030	1,45	0,025	0,005
P07741	Adenine phosphoribosyltransferase OS=Homo sapiens GN=APRT PE=1 SV=2 - [APT_HUMAN]	0,030	1,10	0,027	0,004
Q07507	Dermatopontin OS=Homo sapiens GN=DPT PE=1 SV=2 - [DERM_HUMAN]	0,030	1,41	0,029	0,001
Q6NZI2	Polymerase I and transcript release factor OS=Homo sapiens GN=PTRF PE=1 SV=1 - [PTRF_HUMAN]	0,030	1,13	0,022	0,009

O43739-2	Isoform 2 of Cytohesin-3 OS=Homo sapiens GN=CYTH3 - [CYH3_HUMAN]	0,030	1,12	0,021	0,008
O00468-6	Isoform 6 of Agrin OS=Homo sapiens GN=AGRN - [AGRIN_HUMAN]	0,029	1,45	0,016	0,013
P08865	40S ribosomal protein SA OS=Homo sapiens GN=RPSA PE=1 SV=4 - [RSSA_HUMAN]	0,029	1,21	0,018	0,011
Q93052	Lipoma-preferred partner OS=Homo sapiens GN=LPP PE=1 SV=1 - [LPP_HUMAN]	0,029	1,15	0,025	0,004
P25325	3-mercaptopyruvate sulfurtransferase OS=Homo sapiens GN=MPST PE=1 SV=3 - [THTM_HUMAN]	0,029	1,52	0,025	0,004
P07108	Acyl-CoA-binding protein OS=Homo sapiens GN=DBI PE=1 SV=2 - [ACBP_HUMAN]	0,029	1,31	0,020	0,009
P34741	Syndecan-2 OS=Homo sapiens GN=SDC2 PE=1 SV=2 - [SDC2_HUMAN]	0,029	1,20	0,024	0,005
Q9HB71	Calcyclin-binding protein OS=Homo sapiens GN=CACYBP PE=1 SV=2 - [CYBP_HUMAN]	0,028	1,13	0,028	0,001
P61923	Coatomer subunit zeta-1 OS=Homo sapiens GN=COPZ1 PE=1 SV=1 - [COPZ1_HUMAN]	0,028	1,18	0,027	0,002
Q9Y508-2	Isoform 2 of E3 ubiquitin-protein ligase RNF114 OS=Homo sapiens GN=RNF114 - [RN114_HUMAN]	0,028	1,13	0,026	0,002
Q8IW45-4	Isoform 4 of ATP-dependent (S)-NAD(P)H-hydrate dehydratase OS=Homo sapiens GN=CARKD - [NNRD_HUMAN]	0,028	1,13	0,027	0,001
Q92597	Protein NDRG1 OS=Homo sapiens GN=NDRG1 PE=1 SV=1 - [NDRG1_HUMAN]	0,028	1,18	0,024	0,004
O75718	Cartilage-associated protein OS=Homo sapiens GN=CRTAP PE=1 SV=1 - [CRTAP_HUMAN]	0,027	1,45	0,026	0,001
Q12929	Epidermal growth factor receptor kinase substrate 8 OS=Homo sapiens GN=EPS8 PE=1 SV=1 - [EPS8_HUMAN]	0,027	1,11	0,024	0,003
O60279	Sushi domain-containing protein 5 OS=Homo sapiens GN=SUSD5 PE=1 SV=3 - [SUSD5_HUMAN]	0,027	1,27	0,026	0,002
Q96TA1-2	Isoform 2 of Niban-like protein 1 OS=Homo sapiens GN=FAM129B - [NIBL1_HUMAN]	0,027	1,15	0,019	0,008
Q15785	Mitochondrial import receptor subunit TOM34 OS=Homo sapiens GN=TOMM34 PE=1 SV=2 - [TOM34_HUMAN]	0,027	1,36	0,025	0,003
P11177-2	Isoform 2 of Pyruvate dehydrogenase E1 component subunit beta, mitochondrial OS=Homo sapiens GN=PDHB - [ODPB_HUMAN]	0,027	1,19	0,020	0,008
Q15019	Septin-2 OS=Homo sapiens GN=SEPT2 PE=1 SV=1 - [SEPT2_HUMAN]	0,027	1,08	0,019	0,008
O60936	Nucleolar protein 3 OS=Homo sapiens GN=NOL3 PE=1 SV=2 - [NOL3_HUMAN]	0,027	1,25	0,020	0,006
P20774	Mimecan OS=Homo sapiens GN=OGN PE=1 SV=1 - [MIME_HUMAN]	0,027	1,32	0,022	0,005
P53680-2	Isoform 2 of AP-2 complex subunit sigma OS=Homo sapiens GN=AP2S1 - [AP2S1_HUMAN]	0,027	1,16	0,021	0,006
P43034	Platelet-activating factor acetylhydrolase IB subunit alpha OS=Homo sapiens GN=PAFAH1B1 PE=1 SV=2 - [LIS1_HUMAN]	0,025	1,19	0,017	0,009
P04275	von Willebrand factor OS=Homo sapiens GN=VWF PE=1 SV=4 - [VWF_HUMAN]	0,025	1,79	0,021	0,004
Q15286	Ras-related protein Rab-35 OS=Homo sapiens GN=RAB35 PE=1 SV=1 - [RAB35_HUMAN]	0,025	1,29	0,017	0,009
Q16363-2	Isoform 2 of Laminin subunit alpha-4 OS=Homo sapiens GN=LAMA4 - [LAMA4_HUMAN]	0,025	1,24	0,021	0,004
Q13043-2	Isoform 2 of Serine/threonine-protein kinase 4 OS=Homo sapiens GN=STK4 - [STK4_HUMAN]	0,025	1,12	0,020	0,005
Q8TF74-2	Isoform 2 of WAS/WASL-interacting protein family member 2 OS=Homo sapiens GN=WIPF2 - [WIPF2_HUMAN]	0,025	1,33	0,022	0,002
Q7Z304	MAM domain-containing protein 2 OS=Homo sapiens GN=MAMDC2 PE=2 SV=3 - [MAMC2_HUMAN]	0,025	1,75	0,019	0,006
P21810	Biglycan OS=Homo sapiens GN=BGN PE=1 SV=2 - [PGS1_HUMAN]	0,024	1,33	0,018	0,007
O43760	Synaptogyrin-2 OS=Homo sapiens GN=SYNGR2 PE=1 SV=1 - [SNG2_HUMAN]	0,024	1,29	0,020	0,004
Q92626	Peroxidasin homolog OS=Homo sapiens GN=PXDN PE=1 SV=2 - [PXDN_HUMAN]	0,024	1,19	0,019	0,004
Q15907-2	Isoform 2 of Ras-related protein Rab-11B OS=Homo sapiens GN=RAB11B - [RB11B_HUMAN]	0,024	1,28	0,021	0,003

Q8IWU6	Extracellular sulfatase Sulf-1 OS=Homo sapiens GN=SULF1 PE=1 SV=1 - [SULF1_HUMAN]	0,023	1,55	0,016	0,008
P11279	Lysosome-associated membrane glycoprotein 1 OS=Homo sapiens GN=LAMP1 PE=1 SV=3 - [LAMP1_HUMAN]	0,023	1,39	0,013	0,009
Q86X52	Chondroitin sulfate synthase 1 OS=Homo sapiens GN=CHSY1 PE=1 SV=3 - [CHSS1_HUMAN]	0,022	1,33	0,021	0,001
Q96CG8-3	Isoform 3 of Collagen triple helix repeat-containing protein 1 OS=Homo sapiens GN=CTHRC1 - [CTHR1_HUMAN]	0,020	1,30	0,013	0,007
O00300	Tumor necrosis factor receptor superfamily member 11B OS=Homo sapiens GN=TNFRSF11B PE=1 SV=3 - [TR11B_HUMAN]	0,020	1,37	0,015	0,005
Q9H8L6	Multimerin-2 OS=Homo sapiens GN=MMRN2 PE=1 SV=2 - [MMRN2_HUMAN]	0,020	1,76	0,017	0,003
Q96C24-2	Isoform 2 of Synaptotagmin-like protein 4 OS=Homo sapiens GN=SYTL4 - [SYTL4_HUMAN]	0,018	1,44	0,013	0,005

Proteins downregulated in BAV non-dilated samples

Protein accession number	Protein name	Loading value [a]	Fold change	jack-knife (JK) confidence interval[b]	ABS (Loading value)-ABS(JK)[c]
P01603	Ig kappa chain V-I region Ka OS=Homo sapiens PE=1 SV=1 - [KV111_HUMAN]	-0,050	0,67	0,016	0,034
P17980	26S protease regulatory subunit 6A OS=Homo sapiens GN=PSMC3 PE=1 SV=3 - [PRS6A_HUMAN]	-0,047	0,93	0,031	0,016
P01612	Ig kappa chain V-I region Mev OS=Homo sapiens PE=1 SV=1 - [KV120_HUMAN]	-0,047	0,68	0,021	0,026
Q6DHV7-2	Isoform 2 of Adenosine deaminase-like protein OS=Homo sapiens GN=ADAL - [ADAL_HUMAN]	-0,046	0,73	0,023	0,023
O43150-2	Isoform 2 of Arf-GAP with SH3 domain, ANK repeat and PH domain-containing protein 2 OS=Homo sapiens GN=ASAP2 - [ASAP2_HUMAN]	-0,045	0,80	0,015	0,031
Q9UH62	Armadillo repeat-containing X-linked protein 3 OS=Homo sapiens GN=ARMCX3 PE=1 SV=1 - [ARMX3_HUMAN]	-0,044	0,70	0,018	0,027
Q16537-2	Isoform 2 of Serine/threonine-protein phosphatase 2A 56 kDa regulatory subunit epsilon isoform OS=Homo sapiens GN=PPP2R5E - [2A5E_HUMAN]	-0,044	0,91	0,026	0,018
Q9H6S3	Epidermal growth factor receptor kinase substrate 8-like protein 2 OS=Homo sapiens GN=EPS8L2 PE=1 SV=2 - [ES8L2_HUMAN]	-0,044	0,84	0,025	0,018
Q9H6T3-2	Isoform 2 of RNA polymerase II-associated protein 3 OS=Homo sapiens GN=RPAP3 - [RPAP3_HUMAN]	-0,042	0,80	0,028	0,014
Q9NR09	Baculoviral IAP repeat-containing protein 6 OS=Homo sapiens GN=BIRC6 PE=1 SV=2 - [BIRC6_HUMAN]	-0,041	0,77	0,028	0,013
P00738	Haptoglobin OS=Homo sapiens GN=HP PE=1 SV=1 - [HPT_HUMAN]	-0,041	0,53	0,015	0,026
P01766	Ig heavy chain V-III region BRO OS=Homo sapiens PE=1 SV=1 - [HV305_HUMAN]	-0,041	0,75	0,024	0,017
P01137	Transforming growth factor beta-1 OS=Homo sapiens GN=TGFB1 PE=1 SV=2 - [TGFB1_HUMAN]	-0,041	0,74	0,029	0,012
P10114	Ras-related protein Rap-2a OS=Homo sapiens GN=RAP2A PE=1 SV=1 - [RAP2A_HUMAN]	-0,041	0,48	0,038	0,003
P00751	Complement factor B OS=Homo sapiens GN=CFB PE=1 SV=2 - [CFAB_HUMAN]	-0,040	0,69	0,014	0,026
Q6P179-3	Isoform 3 of Endoplasmic reticulum aminopeptidase 2 OS=Homo sapiens GN=ERAP2 - [ERAP2_HUMAN]	-0,040	0,67	0,026	0,015
Q8TBB5-2	Isoform 2 of Kelch domain-containing protein 4 OS=Homo sapiens GN=KLHDC4 - [KLDC4_HUMAN]	-0,040	0,69	0,028	0,012
P30443	HLA class I histocompatibility antigen, A-1 alpha chain OS=Homo sapiens GN=HLA-A PE=1 SV=1 - [1A01_HUMAN]	-0,040	0,18	0,019	0,021

Q9H4A4	Aminopeptidase B OS=Homo sapiens GN=RNPEP PE=1 SV=2 - [AMPB_HUMAN]	-0,040	0,76	0,031	0,008
P01834	Ig kappa chain C region OS=Homo sapiens GN=IGKC PE=1 SV=1 - [IGKC_HUMAN]	-0,040	0,56	0,025	0,015
Q15386	Ubiquitin-protein ligase E3C OS=Homo sapiens GN=UBE3C PE=1 SV=3 - [UBE3C_HUMAN]	-0,039	0,68	0,032	0,007
Q9NP78-3	Isoform 3 of ATP-binding cassette sub-family B member 9 OS=Homo sapiens GN=ABCB9 - [ABCB9_HUMAN]	-0,039	0,44	0,019	0,020
Q07812-5	Isoform Epsilon of Apoptosis regulator BAX OS=Homo sapiens GN=BAX - [BAX_HUMAN]	-0,039	0,90	0,031	0,008
P01596	Ig kappa chain V-I region CAR OS=Homo sapiens PE=1 SV=1 - [KV104_HUMAN]	-0,039	0,78	0,033	0,006
Q15813	Tubulin-specific chaperone E OS=Homo sapiens GN=TBCE PE=1 SV=1 - [TBCE_HUMAN]	-0,039	0,89	0,022	0,016
P01594	Ig kappa chain V-I region AU OS=Homo sapiens PE=1 SV=1 - [KV102_HUMAN]	-0,038	0,75	0,023	0,015
Q8IWV7	E3 ubiquitin-protein ligase UBR1 OS=Homo sapiens GN=UBR1 PE=1 SV=1 - [UBR1_HUMAN]	-0,038	0,81	0,025	0,013
P01605	Ig kappa chain V-I region Lay OS=Homo sapiens PE=1 SV=1 - [KV113_HUMAN]	-0,038	0,78	0,026	0,012
Q6IQ22	Ras-related protein Rab-12 OS=Homo sapiens GN=RAB12 PE=1 SV=3 - [RAB12_HUMAN]	-0,038	0,76	0,028	0,011
O43670-2	Isoform 2 of BUB3-interacting and GLEBS motif-containing protein ZNF207 OS=Homo sapiens GN=ZNF207 - [ZN207_HUMAN]	-0,038	0,85	0,035	0,003
P04424-3	Isoform 3 of Argininosuccinate lyase OS=Homo sapiens GN=ASL - [ARLY_HUMAN]	-0,038	0,81	0,021	0,018
Q13310-2	Isoform 2 of Polyadenylate-binding protein 4 OS=Homo sapiens GN=PABPC4 - [PABP4_HUMAN]	-0,038	0,85	0,032	0,006
P15586-2	Isoform 2 of N-acetylglucosamine-6-sulfatase OS=Homo sapiens GN=GNS - [GNS_HUMAN]	-0,037	0,89	0,031	0,006
A5D8V6	Vacuolar protein sorting-associated protein 37C OS=Homo sapiens GN=VPS37C PE=1 SV=2 - [VP37C_HUMAN]	-0,037	0,86	0,030	0,007
P52790	Hexokinase-3 OS=Homo sapiens GN=HK3 PE=1 SV=2 - [HXK3_HUMAN]	-0,037	0,62	0,021	0,015
Q9UGT4	Sushi domain-containing protein 2 OS=Homo sapiens GN=SUSD2 PE=1 SV=1 - [SUSD2_HUMAN]	-0,037	0,65	0,032	0,005
P61221	ATP-binding cassette sub-family E member 1 OS=Homo sapiens GN=ABCE1 PE=1 SV=1 - [ABCE1_HUMAN]	-0,037	0,86	0,029	0,008
Q6ZVK8-2	Isoform 2 of 8-oxo-dGDP phosphatase NUDT18 OS=Homo sapiens GN=NUDT18 - [NUD18_HUMAN]	-0,036	0,76	0,028	0,009
Q7Z478	ATP-dependent RNA helicase DHX29 OS=Homo sapiens GN=DHX29 PE=1 SV=2 - [DHX29_HUMAN]	-0,036	0,89	0,030	0,006
Q8IXT5	RNA-binding protein 12B OS=Homo sapiens GN=RBM12B PE=1 SV=2 - [RB12B_HUMAN]	-0,036	0,75	0,027	0,010
P06681	Complement C2 OS=Homo sapiens GN=C2 PE=1 SV=2 - [CO2_HUMAN]	-0,036	0,69	0,017	0,019
P41227-2	Isoform 2 of N-alpha-acetyltransferase 10 OS=Homo sapiens GN=NAA10 - [NAA10_HUMAN]	-0,036	0,80	0,034	0,002
Q7Z6Z7-2	Isoform 2 of E3 ubiquitin-protein ligase HUWE1 OS=Homo sapiens GN=HUWE1 - [HUWE1_HUMAN]	-0,036	0,87	0,030	0,006
Q8NG68	Tubulin--tyrosine ligase OS=Homo sapiens GN=TTL PE=1 SV=2 - [TTL_HUMAN]	-0,036	0,74	0,034	0,002
P48735-2	Isoform 2 of Isocitrate dehydrogenase [NADP], mitochondrial OS=Homo sapiens GN=IDH2 - [IDHP_HUMAN]	-0,036	0,88	0,027	0,009
O75348	V-type proton ATPase subunit G 1 OS=Homo sapiens GN=ATP6V1G1 PE=1 SV=3 - [VATG1_HUMAN]	-0,036	0,87	0,034	0,002
P49789	Bis(5'-adenosyl)-triphosphatase OS=Homo sapiens GN=FHIT PE=1 SV=3 - [FHIT_HUMAN]	-0,036	0,87	0,036	0,000
Q99832	T-complex protein 1 subunit eta OS=Homo sapiens GN=CCT7 PE=1 SV=2 - [TCPH_HUMAN]	-0,036	0,94	0,026	0,009
Q8N108-17	Isoform 7 of Mesoderm induction early response protein 1 OS=Homo sapiens GN=MIER1 - [MIER1_HUMAN]	-0,035	0,79	0,028	0,008
Q9NR45	Sialic acid synthase OS=Homo sapiens GN=NANS PE=1 SV=2 - [SIAS_HUMAN]	-0,035	0,80	0,027	0,008
P42858	Huntingtin OS=Homo sapiens GN=HTT PE=1 SV=2 - [HD_HUMAN]	-0,035	0,77	0,025	0,010

Q5TZA2	Rootletin OS=Homo sapiens GN=CROCC PE=1 SV=1 - [CROCC_HUMAN]	-0,035	0,84	0,027	0,008
P04434	Ig kappa chain V-III region VH (Fragment) OS=Homo sapiens PE=4 SV=1 - [KV310_HUMAN]	-0,035	0,62	0,030	0,005
P01024	Complement C3 OS=Homo sapiens GN=C3 PE=1 SV=2 - [CO3_HUMAN]	-0,035	0,67	0,023	0,012
Q5M775	Cytospin-B OS=Homo sapiens GN=SPECC1 PE=1 SV=1 - [CYTSB_HUMAN]	-0,035	0,74	0,032	0,002
P27361	Mitogen-activated protein kinase 3 OS=Homo sapiens GN=MAPK3 PE=1 SV=4 - [MK03_HUMAN]	-0,035	0,89	0,034	0,000
P09455	Retinol-binding protein 1 OS=Homo sapiens GN=RBP1 PE=1 SV=2 - [RET1_HUMAN]	-0,035	0,78	0,020	0,015
P29622	Kallistatin OS=Homo sapiens GN=SERPINA4 PE=1 SV=3 - [KAIN_HUMAN]	-0,034	0,72	0,029	0,006
Q9Y263	Phospholipase A-2-activating protein OS=Homo sapiens GN=PLAA PE=1 SV=2 - [PLAP_HUMAN]	-0,034	0,79	0,024	0,010
Q92859-2	Isoform 2 of Neogenin OS=Homo sapiens GN=NEO1 - [NEO1_HUMAN]	-0,034	0,84	0,033	0,001
O00231	26S proteasome non-ATPase regulatory subunit 11 OS=Homo sapiens GN=PSMD11 PE=1 SV=3 - [PSD11_HUMAN]	-0,034	0,85	0,018	0,016
Q9NYL9	Tropomodulin-3 OS=Homo sapiens GN=TMOD3 PE=1 SV=1 - [TMOD3_HUMAN]	-0,034	0,75	0,028	0,006
P13716	Delta-aminolevulinic acid dehydratase OS=Homo sapiens GN=ALAD PE=1 SV=1 - [HEM2_HUMAN]	-0,034	0,72	0,025	0,008
Q96GZ6-6	Isoform 6 of Solute carrier family 41 member 3 OS=Homo sapiens GN=SLC41A3 - [S41A3_HUMAN]	-0,034	0,73	0,032	0,001
O14939-4	Isoform PLD2B of Phospholipase D2 OS=Homo sapiens GN=PLD2 - [PLD2_HUMAN]	-0,033	0,85	0,029	0,005
P0CG05	Ig lambda-2 chain C regions OS=Homo sapiens GN=IGLC2 PE=1 SV=1 - [LAC2_HUMAN]	-0,033	0,66	0,031	0,002
Q8N163	Cell cycle and apoptosis regulator protein 2 OS=Homo sapiens GN=CCAR2 PE=1 SV=2 - [CCAR2_HUMAN]	-0,033	0,70	0,022	0,011
Q9H0D6-2	Isoform 2 of 5'-3' exoribonuclease 2 OS=Homo sapiens GN=XRN2 - [XRN2_HUMAN]	-0,033	0,71	0,023	0,010
Q9H3S7	Tyrosine-protein phosphatase non-receptor type 23 OS=Homo sapiens GN=PTPN23 PE=1 SV=1 - [PTN23_HUMAN]	-0,033	0,89	0,028	0,005
Q9HBI0-3	Isoform 3 of Gamma-parvin OS=Homo sapiens GN=PARVG - [PARVG_HUMAN]	-0,033	0,69	0,029	0,004
P01857	Ig gamma-1 chain C region OS=Homo sapiens GN=IGHG1 PE=1 SV=1 - [IGHG1_HUMAN]	-0,033	0,65	0,032	0,001
Q9BSB4	Autophagy-related protein 101 OS=Homo sapiens GN=ATG101 PE=1 SV=1 - [ATGA1_HUMAN]	-0,033	0,83	0,031	0,002
Q9NSY0	Nuclear receptor-binding protein 2 OS=Homo sapiens GN=NRBP2 PE=2 SV=2 - [NRBP2_HUMAN]	-0,032	0,85	0,030	0,003
P20339-2	Isoform 2 of Ras-related protein Rab-5A OS=Homo sapiens GN=RAB5A - [RAB5A_HUMAN]	-0,032	0,70	0,027	0,005
P30566	Adenylosuccinate lyase OS=Homo sapiens GN=ADSL PE=1 SV=2 - [PUR8_HUMAN]	-0,032	0,79	0,032	0,000
Q9NTJ4-3	Isoform 3 of Alpha-mannosidase 2C1 OS=Homo sapiens GN=MAN2C1 - [MA2C1_HUMAN]	-0,032	0,81	0,028	0,005
P06865	Beta-hexosaminidase subunit alpha OS=Homo sapiens GN=HEXA PE=1 SV=2 - [HEXA_HUMAN]	-0,032	0,88	0,032	0,000
P57076	UPF0769 protein C21orf59 OS=Homo sapiens GN=C21orf59 PE=1 SV=1 - [CU059_HUMAN]	-0,032	0,81	0,027	0,006
P17544-5	Isoform 5 of Cyclic AMP-dependent transcription factor ATF-7 OS=Homo sapiens GN=ATF7 - [ATF7_HUMAN]	-0,032	0,74	0,028	0,004
P01743	Ig heavy chain V-I region HG3 OS=Homo sapiens PE=3 SV=1 - [HV102_HUMAN]	-0,032	0,65	0,032	0,000
Q8IXB1-2	Isoform 2 of DnaJ homolog subfamily C member 10 OS=Homo sapiens GN=DNAJC10 - [DJC10_HUMAN]	-0,032	0,85	0,017	0,015
Q9NRW7-2	Isoform 2 of Vacuolar protein sorting-associated protein 45 OS=Homo sapiens GN=VPS45 - [VPS45_HUMAN]	-0,032	0,70	0,023	0,009
P08575-2	Isoform 2 of Receptor-type tyrosine-protein phosphatase C OS=Homo sapiens GN=PTPRC - [PTPRC_HUMAN]	-0,031	0,68	0,008	0,023
Q6P2Q9	Pre-mRNA-processing-splicing factor 8 OS=Homo sapiens GN=PRPF8 PE=1 SV=2 - [PRP8_HUMAN]	-0,031	0,81	0,020	0,011

P42566	Epidermal growth factor receptor substrate 15 OS=Homo sapiens GN=EPS15 PE=1 SV=2 - [EPS15_HUMAN]	-0,031	0,84	0,026	0,005
Q9NZ32	Actin-related protein 10 OS=Homo sapiens GN=ACTR10 PE=1 SV=1 - [ARP10_HUMAN]	-0,031	0,79	0,029	0,001
P52655	Transcription initiation factor IIA subunit 1 OS=Homo sapiens GN=GTF2A1 PE=1 SV=1 - [TF2AA_HUMAN]	-0,031	0,76	0,026	0,004
Q9ULC4-2	Isoform 2 of Malignant T-cell-amplified sequence 1 OS=Homo sapiens GN=MCTS1 - [MCTS1_HUMAN]	-0,031	0,86	0,026	0,004
P02679-2	Isoform Gamma-A of Fibrinogen gamma chain OS=Homo sapiens GN=FGG - [FIBG_HUMAN]	-0,030	0,68	0,016	0,014
P02675	Fibrinogen beta chain OS=Homo sapiens GN=FGB PE=1 SV=2 - [FIBB_HUMAN]	-0,030	0,69	0,020	0,010
Q5JSH3-4	Isoform 4 of WD repeat-containing protein 44 OS=Homo sapiens GN=WDR44 - [WDR44_HUMAN]	-0,030	0,89	0,025	0,005
Q10570	Cleavage and polyadenylation specificity factor subunit 1 OS=Homo sapiens GN=CPSF1 PE=1 SV=2 - [CPSF1_HUMAN]	-0,030	0,86	0,023	0,007
Q75N90	Fibrillin-3 OS=Homo sapiens GN=FBN3 PE=2 SV=3 - [FBN3_HUMAN]	-0,030	0,67	0,027	0,003
Q14624-3	Isoform 3 of Inter-alpha-trypsin inhibitor heavy chain H4 OS=Homo sapiens GN=ITIH4 - [ITIH4_HUMAN]	-0,030	0,72	0,020	0,010
P05155-2	Isoform 2 of Plasma protease C1 inhibitor OS=Homo sapiens GN=SERPING1 - [IC1_HUMAN]	-0,030	0,80	0,024	0,006
Q14457	Beclin-1 OS=Homo sapiens GN=BECN1 PE=1 SV=2 - [BECN1_HUMAN]	-0,030	0,95	0,018	0,012
P49327	Fatty acid synthase OS=Homo sapiens GN=FASN PE=1 SV=3 - [FAS_HUMAN]	-0,030	0,83	0,028	0,002
P27658	Collagen alpha-1(VIII) chain OS=Homo sapiens GN=COL8A1 PE=1 SV=2 - [CO8A1_HUMAN]	-0,030	0,58	0,029	0,001
P08603	Complement factor H OS=Homo sapiens GN=CFH PE=1 SV=4 - [CFAH_HUMAN]	-0,030	0,75	0,018	0,012
Q969Z0-2	Isoform 2 of Protein TBRG4 OS=Homo sapiens GN=TBRG4 - [TBRG4_HUMAN]	-0,029	0,86	0,028	0,002
Q12888	Tumor suppressor p53-binding protein 1 OS=Homo sapiens GN=TP53BP1 PE=1 SV=2 - [TP53B_HUMAN]	-0,029	0,85	0,029	0,001
P02768	Serum albumin OS=Homo sapiens GN=ALB PE=1 SV=2 - [ALBU_HUMAN]	-0,029	0,76	0,028	0,001
P67936-2	Isoform 2 of Tropomyosin alpha-4 chain OS=Homo sapiens GN=TPM4 - [TPM4_HUMAN]	-0,029	0,78	0,028	0,001
P02671-2	Isoform 2 of Fibrinogen alpha chain OS=Homo sapiens GN=FGA - [FIBA_HUMAN]	-0,029	0,67	0,021	0,008
Q9Y2X3	Nucleolar protein 58 OS=Homo sapiens GN=NOP58 PE=1 SV=1 - [NOP58_HUMAN]	-0,029	0,73	0,026	0,003
P02787	Serotransferrin OS=Homo sapiens GN=TF PE=1 SV=3 - [TRFE_HUMAN]	-0,028	0,82	0,027	0,002
P36894	Bone morphogenetic protein receptor type-1A OS=Homo sapiens GN=BMPR1A PE=1 SV=2 - [BMR1A_HUMAN]	-0,028	0,84	0,025	0,003
Q08AG7	Mitotic-spindle organizing protein 1 OS=Homo sapiens GN=MZT1 PE=1 SV=2 - [MZT1_HUMAN]	-0,028	0,83	0,018	0,010
P18428	Lipopolysaccharide-binding protein OS=Homo sapiens GN=LBP PE=1 SV=3 - [LBP_HUMAN]	-0,028	0,68	0,020	0,008
P54578	Ubiquitin carboxyl-terminal hydrolase 14 OS=Homo sapiens GN=USP14 PE=1 SV=3 - [UBP14_HUMAN]	-0,028	0,95	0,021	0,006
Q8TAQ9-2	Isoform 2 of SUN domain-containing protein 3 OS=Homo sapiens GN=SUN3 - [SUN3_HUMAN]	-0,028	0,64	0,022	0,006
P42704	Leucine-rich PPR motif-containing protein, mitochondrial OS=Homo sapiens GN=LRPPRC PE=1 SV=3 - [LPPRC_HUMAN]	-0,027	0,94	0,024	0,004
O00232-2	Isoform 2 of 26S proteasome non-ATPase regulatory subunit 12 OS=Homo sapiens GN=PSMD12 - [PSD12_HUMAN]	-0,027	0,83	0,025	0,002
P46977-2	Isoform 2 of Dolichyl-diphosphooligosaccharide--protein glycosyltransferase subunit STT3A OS=Homo sapiens GN=STT3A - [STT3A_HUMAN]	-0,027	0,90	0,025	0,002
Q9BVJ6-2	Isoform 2 of U3 small nucleolar RNA-associated protein 14 homolog A OS=Homo sapiens	-0,027	0,86	0,024	0,003

	GN=UTP14A - [UT14A_HUMAN]				
Q92616	Translational activator GCN1 OS=Homo sapiens GN=GCN1L1 PE=1 SV=6 - [GCN1L_HUMAN]	-0,027	0,94	0,023	0,003
Q96T58	Msx2-interacting protein OS=Homo sapiens GN=SPEN PE=1 SV=1 - [MINT_HUMAN]	-0,026	0,93	0,018	0,009
Q9NQE9	Histidine triad nucleotide-binding protein 3 OS=Homo sapiens GN=HINT3 PE=1 SV=1 - [HINT3_HUMAN]	-0,026	0,86	0,024	0,002
P39900	Macrophage metalloelastase OS=Homo sapiens GN=MMP12 PE=1 SV=1 - [MMP12_HUMAN]	-0,026	0,64	0,023	0,002
Q16799	Reticulon-1 OS=Homo sapiens GN=RTN1 PE=1 SV=1 - [RTN1_HUMAN]	-0,025	0,77	0,024	0,001
Q9NWY4	UPF0609 protein C4orf27 OS=Homo sapiens GN=C4orf27 PE=1 SV=2 - [CD027_HUMAN]	-0,025	0,89	0,012	0,013
P78527	DNA-dependent protein kinase catalytic subunit OS=Homo sapiens GN=PRKDC PE=1 SV=3 - [PRKDC_HUMAN]	-0,025	0,92	0,023	0,002
Q8IX12-2	Isoform 2 of Cell division cycle and apoptosis regulator protein 1 OS=Homo sapiens GN=CCAR1 - [CCAR1_HUMAN]	-0,025	0,84	0,017	0,007
Q5XPI4	E3 ubiquitin-protein ligase RNF123 OS=Homo sapiens GN=RNF123 PE=1 SV=1 - [RN123_HUMAN]	-0,023	0,88	0,017	0,006
P19971	Thymidine phosphorylase OS=Homo sapiens GN=TYMP PE=1 SV=2 - [TYPH_HUMAN]	-0,022	0,87	0,017	0,005
P07358	Complement component C8 beta chain OS=Homo sapiens GN=C8B PE=1 SV=3 - [CO8B_HUMAN]	-0,022	0,83	0,017	0,005

Supplementary Table 4b. Loadings and jack-knife (JK) confidence intervals for significant proteins in dilated samples, sorted according to loading value. OPLS-DA model is based on TAV and BAV being added as a Y variable. The significance was calculated as ABS (loading) – ABS (jack-knife confidence interval); the positive values indicated significantly expressed proteins.

Protein accession number	Protein name	Loading value [a]	Fold change	jack-knife (JK) confidence interval[b]	ABS (Loading value)-ABS(JK)[c]
	**Proteins upregulated in BAV dilated samples**				
O75525-2	Isoform 2 of KH domain-containing, RNA-binding, signal transduction-associated protein 3 OS=Homo sapiens GN=KHDRBS3 - [KHDR3_HUMAN]	0,038	1,95	0,017	0,021
P56945-4	Isoform 4 of Breast cancer anti-estrogen resistance protein 1 OS=Homo sapiens GN=BCAR1 - [BCAR1_HUMAN]	0,038	1,32	0,008	0,031
Q92620	Pre-mRNA-splicing factor ATP-dependent RNA helicase PRP16 OS=Homo sapiens GN=DHX38 PE=1 SV=2 - [PRP16_HUMAN]	0,038	1,35	0,025	0,013
O60547-2	Isoform 2 of GDP-mannose 4,6 dehydratase OS=Homo sapiens GN=GMDS - [GMDS_HUMAN]	0,037	1,65	0,012	0,025
O14907	Tax1-binding protein 3 OS=Homo sapiens GN=TAX1BP3 PE=1 SV=2 - [TX1B3_HUMAN]	0,037	1,16	0,022	0,015
Q53HC5	Kelch-like protein 26 OS=Homo sapiens GN=KLHL26 PE=1 SV=2 - [KLH26_HUMAN]	0,037	1,28	0,021	0,016
Q9NQX7-2	Isoform 2 of Integral membrane protein 2C OS=Homo sapiens GN=ITM2C - [ITM2C_HUMAN]	0,037	1,79	0,021	0,016
O60637-3	Isoform 3 of Tetraspanin-3 OS=Homo sapiens GN=TSPAN3 - [TSN3_HUMAN]	0,036	1,55	0,018	0,019
Q15785	Mitochondrial import receptor subunit TOM34 OS=Homo sapiens GN=TOMM34 PE=1 SV=2 - [TOM34_HUMAN]	0,036	1,55	0,016	0,020

Q8TF09	Dynein light chain roadblock-type 2 OS=Homo sapiens GN=DYNLRB2 PE=1 SV=1 - [DLRB2_HUMAN]	0,036	1,47	0,018	0,018
Q96HN2-3	Isoform 3 of Adenosylhomocysteinase 3 OS=Homo sapiens GN=AHCYL2 - [SAHH3_HUMAN]	0,036	1,39	0,028	0,008
P78356	Phosphatidylinositol 5-phosphate 4-kinase type-2 beta OS=Homo sapiens GN=PIP4K2B PE=1 SV=1 - [PI42B_HUMAN]	0,036	1,48	0,016	0,020
Q8IVH8-3	Isoform 3 of Mitogen-activated protein kinase kinase kinase kinase 3 OS=Homo sapiens GN=MAP4K3 - [M4K3_HUMAN]	0,036	1,39	0,016	0,019
Q9H7D0	Dedicator of cytokinesis protein 5 OS=Homo sapiens GN=DOCK5 PE=1 SV=3 - [DOCK5_HUMAN]	0,036	1,58	0,015	0,021
Q08722-2	Isoform OA3-293 of Leukocyte surface antigen CD47 OS=Homo sapiens GN=CD47 - [CD47_HUMAN]	0,035	1,68	0,021	0,015
P27986	Phosphatidylinositol 3-kinase regulatory subunit alpha OS=Homo sapiens GN=PIK3R1 PE=1 SV=2 - [P85A_HUMAN]	0,035	1,35	0,026	0,010
Q9BW83-2	Isoform 2 of Intraflagellar transport protein 27 homolog OS=Homo sapiens GN=IFT27 - [IFT27_HUMAN]	0,035	1,59	0,014	0,021
Q9Y5Z4	Heme-binding protein 2 OS=Homo sapiens GN=HEBP2 PE=1 SV=1 - [HEBP2_HUMAN]	0,035	1,40	0,015	0,019
O43739-2	Isoform 2 of Cytohesin-3 OS=Homo sapiens GN=CYTH3 - [CYH3_HUMAN]	0,035	1,41	0,013	0,022
P09211	Glutathione S-transferase P OS=Homo sapiens GN=GSTP1 PE=1 SV=2 - [GSTP1_HUMAN]	0,035	1,26	0,008	0,026
Q96RF0-2	Isoform 2 of Sorting nexin-18 OS=Homo sapiens GN=SNX18 - [SNX18_HUMAN]	0,035	1,35	0,011	0,024
Q8TF74-2	Isoform 2 of WAS/WASL-interacting protein family member 2 OS=Homo sapiens GN=WIPF2 - [WIPF2_HUMAN]	0,035	1,29	0,026	0,009
P08237-2	Isoform 2 of ATP-dependent 6-phosphofructokinase, muscle type OS=Homo sapiens GN=PFKM - [PFKAM_HUMAN]	0,034	1,59	0,018	0,017
O60279	Sushi domain-containing protein 5 OS=Homo sapiens GN=SUSD5 PE=1 SV=3 - [SUSD5_HUMAN]	0,034	1,88	0,011	0,023
Q96P44-3	Isoform 3 of Collagen alpha-1(XXI) chain OS=Homo sapiens GN=COL21A1 - [COLA1_HUMAN]	0,034	2,02	0,016	0,018
P48745	Protein NOV homolog OS=Homo sapiens GN=NOV PE=1 SV=1 - [NOV_HUMAN]	0,034	1,56	0,022	0,012
P00441	Superoxide dismutase [Cu-Zn] OS=Homo sapiens GN=SOD1 PE=1 SV=2 - [SODC_HUMAN]	0,034	1,37	0,016	0,018
O75112-2	Isoform 2 of LIM domain-binding protein 3 OS=Homo sapiens GN=LDB3 - [LDB3_HUMAN]	0,034	2,19	0,012	0,022
Q96CT7	Coiled-coil domain-containing protein 124 OS=Homo sapiens GN=CCDC124 PE=1 SV=1 - [CC124_HUMAN]	0,034	1,29	0,016	0,018
Q9BSD7	Cancer-related nucleoside-triphosphatase OS=Homo sapiens GN=NTPCR PE=1 SV=1 - [NTPCR_HUMAN]	0,034	1,85	0,023	0,011
P62979	Ubiquitin-40S ribosomal protein S27a OS=Homo sapiens GN=RPS27A PE=1 SV=2 - [RS27A_HUMAN]	0,034	1,30	0,023	0,011
P07902	Galactose-1-phosphate uridylyltransferase OS=Homo sapiens GN=GALT PE=1 SV=3 - [GALT_HUMAN]	0,034	1,27	0,020	0,013
O15357	Phosphatidylinositol 3,4,5-trisphosphate 5-phosphatase 2 OS=Homo sapiens GN=INPPL1 PE=1 SV=2 - [SHIP2_HUMAN]	0,034	1,16	0,010	0,024
O14964-2	Isoform 2 of Hepatocyte growth factor-regulated tyrosine kinase substrate OS=Homo sapiens GN=HGS - [HGS_HUMAN]	0,033	1,28	0,027	0,007
O94760	N(G),N(G)-dimethylarginine dimethylaminohydrolase 1 OS=Homo sapiens GN=DDAH1 PE=1 SV=3 - [DDAH1_HUMAN]	0,033	2,10	0,018	0,015
Q9H0E2	Toll-interacting protein OS=Homo sapiens GN=TOLLIP PE=1 SV=1 - [TOLIP_HUMAN]	0,033	1,31	0,024	0,009
Q9C0D5-2	Isoform 2 of Protein TANC1 OS=Homo sapiens GN=TANC1 - [TANC1_HUMAN]	0,033	1,32	0,021	0,013
O15231-5	Isoform 5 of Zinc finger protein 185 OS=Homo sapiens GN=ZNF185 - [ZN185_HUMAN]	0,033	2,02	0,017	0,017
Q9NSK0	Kinesin light chain 4 OS=Homo sapiens GN=KLC4 PE=1 SV=3 - [KLC4_HUMAN]	0,033	1,18	0,020	0,013
O95562	Vesicle transport protein SFT2B OS=Homo sapiens GN=SFT2D2 PE=1 SV=1 - [SFT2B_HUMAN]	0,033	1,29	0,020	0,013

O75718	Cartilage-associated protein OS=Homo sapiens GN=CRTAP PE=1 SV=1 - [CRTAP_HUMAN]	0,033	2,00	0,017	0,016
Q9NZN5-2	Isoform 2 of Rho guanine nucleotide exchange factor 12 OS=Homo sapiens GN=ARHGEF12 - [ARHGC_HUMAN]	0,033	1,35	0,021	0,012
Q9NRF8	CTP synthase 2 OS=Homo sapiens GN=CTPS2 PE=1 SV=1 - [PYRG2_HUMAN]	0,033	1,91	0,018	0,015
Q14974	Importin subunit beta-1 OS=Homo sapiens GN=KPNB1 PE=1 SV=2 - [IMB1_HUMAN]	0,033	1,72	0,023	0,009
P50579	Methionine aminopeptidase 2 OS=Homo sapiens GN=METAP2 PE=1 SV=1 - [MAP2_HUMAN]	0,033	1,16	0,020	0,012
Q9NZZ3	Charged multivesicular body protein 5 OS=Homo sapiens GN=CHMP5 PE=1 SV=1 - [CHMP5_HUMAN]	0,033	1,17	0,019	0,014
Q99439	Calponin-2 OS=Homo sapiens GN=CNN2 PE=1 SV=4 - [CNN2_HUMAN]	0,033	1,40	0,024	0,008
Q9UKG1	DCC-interacting protein 13-alpha OS=Homo sapiens GN=APPL1 PE=1 SV=1 - [DP13A_HUMAN]	0,033	1,24	0,016	0,017
Q14393-3	Isoform 3 of Growth arrest-specific protein 6 OS=Homo sapiens GN=GAS6 - [GAS6_HUMAN]	0,033	1,44	0,022	0,010
P33527-8	Isoform 8 of Multidrug resistance-associated protein 1 OS=Homo sapiens GN=ABCC1 - [MRP1_HUMAN]	0,032	1,53	0,021	0,012
Q06830	Peroxiredoxin-1 OS=Homo sapiens GN=PRDX1 PE=1 SV=1 - [PRDX1_HUMAN]	0,032	1,22	0,024	0,009
Q99747-2	Isoform 2 of Gamma-soluble NSF attachment protein OS=Homo sapiens GN=NAPG - [SNAG_HUMAN]	0,032	1,28	0,014	0,018
Q99518	Dimethylaniline monooxygenase [N-oxide-forming] 2 OS=Homo sapiens GN=FMO2 PE=1 SV=4 - [FMO2_HUMAN]	0,032	1,38	0,015	0,017
P09417	Dihydropteridine reductase OS=Homo sapiens GN=QDPR PE=1 SV=2 - [DHPR_HUMAN]	0,032	1,33	0,021	0,011
P15374	Ubiquitin carboxyl-terminal hydrolase isozyme L3 OS=Homo sapiens GN=UCHL3 PE=1 SV=1 - [UCHL3_HUMAN]	0,032	1,12	0,026	0,006
P35249-2	Isoform 2 of Replication factor C subunit 4 OS=Homo sapiens GN=RFC4 - [RFC4_HUMAN]	0,032	1,27	0,022	0,010
P05067-7	Isoform L-APP733 of Amyloid beta A4 protein OS=Homo sapiens GN=APP - [A4_HUMAN]	0,032	2,06	0,025	0,007
Q96T58	Msx2-interacting protein OS=Homo sapiens GN=SPEN PE=1 SV=1 - [MINT_HUMAN]	0,032	1,21	0,026	0,006
Q9H6S3	Epidermal growth factor receptor kinase substrate 8-like protein 2 OS=Homo sapiens GN=EPS8L2 PE=1 SV=2 - [ES8L2_HUMAN]	0,032	1,21	0,015	0,016
O95295	SNARE-associated protein Snapin OS=Homo sapiens GN=SNAPIN PE=1 SV=1 - [SNAPN_HUMAN]	0,032	1,27	0,021	0,010
P17812-2	Isoform 2 of CTP synthase 1 OS=Homo sapiens GN=CTPS1 - [PYRG1_HUMAN]	0,032	1,33	0,005	0,026
Q14011	Cold-inducible RNA-binding protein OS=Homo sapiens GN=CIRBP PE=1 SV=1 - [CIRBP_HUMAN]	0,032	1,96	0,018	0,013
Q14156-3	Isoform 3 of Protein EFR3 homolog A OS=Homo sapiens GN=EFR3A - [EFR3A_HUMAN]	0,032	1,22	0,009	0,022
Q14204	Cytoplasmic dynein 1 heavy chain 1 OS=Homo sapiens GN=DYNC1H1 PE=1 SV=5 - [DYHC1_HUMAN]	0,032	1,15	0,011	0,020
Q9P0K7-4	Isoform 4 of Ankycorbin OS=Homo sapiens GN=RAI14 - [RAI14_HUMAN]	0,032	1,23	0,018	0,014
Q9UKX5	Integrin alpha-11 OS=Homo sapiens GN=ITGA11 PE=1 SV=2 - [ITA11_HUMAN]	0,032	1,37	0,016	0,016
P25325	3-mercaptopyruvate sulfurtransferase OS=Homo sapiens GN=MPST PE=1 SV=3 - [THTM_HUMAN]	0,031	2,02	0,026	0,006
Q13126-7	Isoform 7 of S-methyl-5'-thioadenosine phosphorylase OS=Homo sapiens GN=MTAP - [MTAP_HUMAN]	0,031	1,65	0,015	0,016
Q8WUP2	Filamin-binding LIM protein 1 OS=Homo sapiens GN=FBLIM1 PE=1 SV=2 - [FBLI1_HUMAN]	0,031	1,36	0,015	0,017
O15061	Synemin OS=Homo sapiens GN=SYNM PE=1 SV=2 - [SYNEM_HUMAN]	0,031	1,29	0,013	0,018
P10644-2	Isoform 2 of cAMP-dependent protein kinase type I-alpha regulatory subunit OS=Homo sapiens GN=PRKAR1A - [KAP0_HUMAN]	0,031	1,29	0,013	0,018
Q13683-9	Isoform Alpha-7X2DB of Integrin alpha-7 OS=Homo sapiens GN=ITGA7 - [ITA7_HUMAN]	0,031	1,62	0,020	0,011
P35080-2	Isoform IIb of Profilin-2 OS=Homo sapiens GN=PFN2 - [PROF2_HUMAN]	0,031	1,26	0,009	0,022

O94875-2	Isoform 2 of Sorbin and SH3 domain-containing protein 2 OS=Homo sapiens GN=SORBS2 - [SRBS2_HUMAN]	0,031	1,38	0,025	0,006
Q6EMK4	Vasorin OS=Homo sapiens GN=VASN PE=1 SV=1 - [VASN_HUMAN]	0,031	1,46	0,016	0,015
Q9NRR1	Cytokine-like protein 1 OS=Homo sapiens GN=CYTL1 PE=1 SV=1 - [CYTL1_HUMAN]	0,031	3,03	0,029	0,002
Q96N67-4	Isoform 4 of Dedicator of cytokinesis protein 7 OS=Homo sapiens GN=DOCK7 - [DOCK7_HUMAN]	0,031	1,15	0,022	0,009
P09525	Annexin A4 OS=Homo sapiens GN=ANXA4 PE=1 SV=4 - [ANXA4_HUMAN]	0,031	1,18	0,023	0,008
Q9H330-3	Isoform 3 of Transmembrane protein 245 OS=Homo sapiens GN=TMEM245 - [TM245_HUMAN]	0,031	1,41	0,015	0,016
Q6PCE3	Glucose 1,6-bisphosphate synthase OS=Homo sapiens GN=PGM2L1 PE=1 SV=3 - [PGM2L_HUMAN]	0,031	1,51	0,019	0,012
P63151	Serine/threonine-protein phosphatase 2A 55 kDa regulatory subunit B alpha isoform OS=Homo sapiens GN=PPP2R2A PE=1 SV=1 - [2ABA_HUMAN]	0,031	1,58	0,024	0,007
O14576-4	Isoform 4 of Cytoplasmic dynein 1 intermediate chain 1 OS=Homo sapiens GN=DYNC1I1 - [DC1I1_HUMAN]	0,031	1,55	0,012	0,018
P53582	Methionine aminopeptidase 1 OS=Homo sapiens GN=METAP1 PE=1 SV=2 - [MAP11_HUMAN]	0,031	1,24	0,023	0,008
P14649	Myosin light chain 6B OS=Homo sapiens GN=MYL6B PE=1 SV=1 - [MYL6B_HUMAN]	0,031	1,33	0,011	0,020
P46937-5	Isoform 5 of Transcriptional coactivator YAP1 OS=Homo sapiens GN=YAP1 - [YAP1_HUMAN]	0,030	1,41	0,016	0,014
P35580	Myosin-10 OS=Homo sapiens GN=MYH10 PE=1 SV=3 - [MYH10_HUMAN]	0,030	1,40	0,008	0,023
Q9HB71	Calcyclin-binding protein OS=Homo sapiens GN=CACYBP PE=1 SV=2 - [CYBP_HUMAN]	0,030	1,07	0,015	0,015
Q8TDB4	Protein MGARP OS=Homo sapiens GN=MGARP PE=1 SV=1 - [HUMMR_HUMAN]	0,030	1,75	0,022	0,009
Q13393-4	Isoform PLD1D of Phospholipase D1 OS=Homo sapiens GN=PLD1 - [PLD1_HUMAN]	0,030	1,22	0,012	0,018
P11362-6	Isoform 12 of Fibroblast growth factor receptor 1 OS=Homo sapiens GN=FGFR1 - [FGFR1_HUMAN]	0,030	1,26	0,012	0,018
P50995-2	Isoform 2 of Annexin A11 OS=Homo sapiens GN=ANXA11 - [ANX11_HUMAN]	0,030	1,26	0,013	0,017
P15559-3	Isoform 3 of NAD(P)H dehydrogenase [quinone] 1 OS=Homo sapiens GN=NQO1 - [NQO1_HUMAN]	0,030	2,96	0,019	0,011
P53041	Serine/threonine-protein phosphatase 5 OS=Homo sapiens GN=PPP5C PE=1 SV=1 - [PPP5_HUMAN]	0,030	1,23	0,012	0,019
P06756-3	Isoform 3 of Integrin alpha-V OS=Homo sapiens GN=ITGAV - [ITAV_HUMAN]	0,030	1,31	0,011	0,020
P52943	Cysteine-rich protein 2 OS=Homo sapiens GN=CRIP2 PE=1 SV=1 - [CRIP2_HUMAN]	0,030	1,36	0,021	0,009
P20338	Ras-related protein Rab-4A OS=Homo sapiens GN=RAB4A PE=1 SV=3 - [RAB4A_HUMAN]	0,030	1,26	0,014	0,016
P08758	Annexin A5 OS=Homo sapiens GN=ANXA5 PE=1 SV=2 - [ANXA5_HUMAN]	0,030	1,19	0,021	0,009
Q5XXA6-3	Isoform 3 of Anoctamin-1 OS=Homo sapiens GN=ANO1 - [ANO1_HUMAN]	0,030	1,70	0,011	0,019
P28074-3	Isoform 3 of Proteasome subunit beta type-5 OS=Homo sapiens GN=PSMB5 - [PSB5_HUMAN]	0,030	1,42	0,015	0,015
Q16643	Drebrin OS=Homo sapiens GN=DBN1 PE=1 SV=4 - [DREB_HUMAN]	0,030	1,28	0,018	0,012
P06396-2	Isoform 2 of Gelsolin OS=Homo sapiens GN=GSN - [GELS_HUMAN]	0,030	1,26	0,016	0,014
Q9H098	Protein FAM107B OS=Homo sapiens GN=FAM107B PE=1 SV=1 - [F107B_HUMAN]	0,030	1,45	0,021	0,009
O00244	Copper transport protein ATOX1 OS=Homo sapiens GN=ATOX1 PE=1 SV=1 - [ATOX1_HUMAN]	0,030	1,18	0,017	0,013
P17174	Aspartate aminotransferase, cytoplasmic OS=Homo sapiens GN=GOT1 PE=1 SV=3 - [AATC_HUMAN]	0,030	1,12	0,018	0,012
Q96NS5	Ankyrin repeat and SOCS box protein 16 OS=Homo sapiens GN=ASB16 PE=1 SV=2 - [ASB16_HUMAN]	0,030	2,02	0,025	0,005
Q9P2B2	Prostaglandin F2 receptor negative regulator OS=Homo sapiens GN=PTGFRN PE=1 SV=2 - [FPRP_HUMAN]	0,030	1,41	0,026	0,004
P04083	Annexin A1 OS=Homo sapiens GN=ANXA1 PE=1 SV=2 - [ANXA1_HUMAN]	0,030	1,19	0,014	0,016
P62879	Guanine nucleotide-binding protein G(I)/G(S)/G(T) subunit beta-2 OS=Homo sapiens GN=GNB2 PE=1	0,030	1,20	0,023	0,007

	SV=3 - [GBB2_HUMAN]				
P12931	Proto-oncogene tyrosine-protein kinase Src OS=Homo sapiens GN=SRC PE=1 SV=3 - [SRC_HUMAN]	0,030	1,25	0,024	0,006
Q15111	Inactive phospholipase C-like protein 1 OS=Homo sapiens GN=PLCL1 PE=1 SV=3 - [PLCL1_HUMAN]	0,030	1,42	0,010	0,020
P30086	Phosphatidylethanolamine-binding protein 1 OS=Homo sapiens GN=PEBP1 PE=1 SV=3 - [PEBP1_HUMAN]	0,030	1,34	0,019	0,011
Q08380	Galectin-3-binding protein OS=Homo sapiens GN=LGALS3BP PE=1 SV=1 - [LG3BP_HUMAN]	0,030	1,92	0,024	0,006
Q92934	Bcl2-associated agonist of cell death OS=Homo sapiens GN=BAD PE=1 SV=3 - [BAD_HUMAN]	0,029	1,16	0,014	0,016
Q9Y4G8	Rap guanine nucleotide exchange factor 2 OS=Homo sapiens GN=RAPGEF2 PE=1 SV=1 - [RPGF2_HUMAN]	0,029	1,29	0,014	0,016
Q9UBQ0	Vacuolar protein sorting-associated protein 29 OS=Homo sapiens GN=VPS29 PE=1 SV=1 - [VPS29_HUMAN]	0,029	1,29	0,021	0,009
Q01995	Transgelin OS=Homo sapiens GN=TAGLN PE=1 SV=4 - [TAGL_HUMAN]	0,029	1,37	0,015	0,015
Q96HC4	PDZ and LIM domain protein 5 OS=Homo sapiens GN=PDLIM5 PE=1 SV=5 - [PDLI5_HUMAN]	0,029	1,16	0,013	0,016
Q14247	Src substrate cortactin OS=Homo sapiens GN=CTTN PE=1 SV=2 - [SRC8_HUMAN]	0,029	1,31	0,013	0,017
P50502	Hsc70-interacting protein OS=Homo sapiens GN=ST13 PE=1 SV=2 - [F10A1_HUMAN]	0,029	1,18	0,015	0,015
P57087-2	Isoform 2 of Junctional adhesion molecule B OS=Homo sapiens GN=JAM2 - [JAM2_HUMAN]	0,029	1,57	0,014	0,015
Q8WZA0	Protein LZIC OS=Homo sapiens GN=LZIC PE=1 SV=1 - [LZIC_HUMAN]	0,029	1,15	0,023	0,006
Q14678-2	Isoform 2 of KN motif and ankyrin repeat domain-containing protein 1 OS=Homo sapiens GN=KANK1 - [KANK1_HUMAN]	0,029	1,56	0,013	0,016
P0CAP2-3	Isoform 3 of DNA-directed RNA polymerase II subunit GRINL1A OS=Homo sapiens GN=POLR2M - [GRL1A_HUMAN]	0,029	1,22	0,013	0,016
Q92734-4	Isoform 4 of Protein TFG OS=Homo sapiens GN=TFG - [TFG_HUMAN]	0,029	1,26	0,014	0,014
Q12979-4	Isoform 4 of Active breakpoint cluster region-related protein OS=Homo sapiens GN=ABR - [ABR_HUMAN]	0,029	1,27	0,018	0,011
Q9NPC6	Myozenin-2 OS=Homo sapiens GN=MYOZ2 PE=1 SV=1 - [MYOZ2_HUMAN]	0,029	1,73	0,022	0,007
Q4G0F5	Vacuolar protein sorting-associated protein 26B OS=Homo sapiens GN=VPS26B PE=1 SV=2 - [VP26B_HUMAN]	0,029	1,25	0,012	0,017
P50416-2	Isoform 2 of Carnitine O-palmitoyltransferase 1, liver isoform OS=Homo sapiens GN=CPT1A - [CPT1A_HUMAN]	0,029	1,30	0,019	0,010
Q06124-2	Isoform 2 of Tyrosine-protein phosphatase non-receptor type 11 OS=Homo sapiens GN=PTPN11 - [PTN11_HUMAN]	0,029	1,28	0,010	0,018
Q99426-2	Isoform 2 of Tubulin-folding cofactor B OS=Homo sapiens GN=TBCB - [TBCB_HUMAN]	0,029	1,26	0,014	0,014
O95071-2	Isoform 2 of E3 ubiquitin-protein ligase UBR5 OS=Homo sapiens GN=UBR5 - [UBR5_HUMAN]	0,029	1,17	0,018	0,011
Q07092-2	Isoform 2 of Collagen alpha-1(XVI) chain OS=Homo sapiens GN=COL16A1 - [COGA1_HUMAN]	0,029	1,89	0,020	0,009
P29558-2	Isoform 2 of RNA-binding motif, single-stranded-interacting protein 1 OS=Homo sapiens GN=RBMS1 - [RBMS1_HUMAN]	0,029	1,15	0,016	0,013
Q9NZU5	LIM and cysteine-rich domains protein 1 OS=Homo sapiens GN=LMCD1 PE=1 SV=1 - [LMCD1_HUMAN]	0,029	1,40	0,010	0,018
O95757	Heat shock 70 kDa protein 4L OS=Homo sapiens GN=HSPA4L PE=1 SV=3 - [HS74L_HUMAN]	0,029	1,30	0,009	0,019
P26447	Protein S100-A4 OS=Homo sapiens GN=S100A4 PE=1 SV=1 - [S10A4_HUMAN]	0,029	1,28	0,023	0,006
Q9UBX5	Fibulin-5 OS=Homo sapiens GN=FBLN5 PE=1 SV=1 - [FBLN5_HUMAN]	0,029	1,47	0,011	0,017
Q16881-5	Isoform 5 of Thioredoxin reductase 1, cytoplasmic OS=Homo sapiens GN=TXNRD1 - [TRXR1_HUMAN]	0,029	1,26	0,012	0,017

Q9UBQ7	Glyoxylate reductase/hydroxypyruvate reductase OS=Homo sapiens GN=GRHPR PE=1 SV=1 - [GRHPR_HUMAN]	0,029	1,44	0,013	0,016
O00159-2	Isoform 2 of Unconventional myosin-Ic OS=Homo sapiens GN=MYO1C - [MYO1C_HUMAN]	0,029	1,35	0,014	0,015
Q9NR12-2	Isoform 2 of PDZ and LIM domain protein 7 OS=Homo sapiens GN=PDLIM7 - [PDLI7_HUMAN]	0,028	1,34	0,009	0,020
Q6ZVM7	TOM1-like protein 2 OS=Homo sapiens GN=TOM1L2 PE=1 SV=1 - [TM1L2_HUMAN]	0,028	1,31	0,027	0,002
P10915	Hyaluronan and proteoglycan link protein 1 OS=Homo sapiens GN=HAPLN1 PE=2 SV=2 - [HPLN1_HUMAN]	0,028	2,46	0,021	0,007
Q8NF91-4	Isoform 4 of Nesprin-1 OS=Homo sapiens GN=SYNE1 - [SYNE1_HUMAN]	0,028	1,27	0,017	0,011
Q9NRA8-2	Isoform 2 of Eukaryotic translation initiation factor 4E transporter OS=Homo sapiens GN=EIF4ENIF1 - [4ET_HUMAN]	0,028	1,25	0,019	0,010
Q06828	Fibromodulin OS=Homo sapiens GN=FMOD PE=1 SV=2 - [FMOD_HUMAN]	0,028	1,49	0,020	0,009
O60814	Histone H2B type 1-K OS=Homo sapiens GN=HIST1H2BK PE=1 SV=3 - [H2B1K_HUMAN]	0,028	1,24	0,025	0,003
Q9H7C9-3	Isoform 3 of Mth938 domain-containing protein OS=Homo sapiens GN=AAMDC - [AAMDC_HUMAN]	0,028	1,51	0,027	0,002
P42330	Aldo-keto reductase family 1 member C3 OS=Homo sapiens GN=AKR1C3 PE=1 SV=4 - [AK1C3_HUMAN]	0,028	1,40	0,012	0,016
Q6UVK1	Chondroitin sulfate proteoglycan 4 OS=Homo sapiens GN=CSPG4 PE=1 SV=2 - [CSPG4_HUMAN]	0,028	1,26	0,021	0,008
Q969P0-3	Isoform 3 of Immunoglobulin superfamily member 8 OS=Homo sapiens GN=IGSF8 - [IGSF8_HUMAN]	0,028	1,38	0,016	0,012
O75751	Solute carrier family 22 member 3 OS=Homo sapiens GN=SLC22A3 PE=1 SV=1 - [S22A3_HUMAN]	0,028	2,07	0,020	0,008
Q9BXF6	Rab11 family-interacting protein 5 OS=Homo sapiens GN=RAB11FIP5 PE=1 SV=1 - [RFIP5_HUMAN]	0,028	1,28	0,020	0,008
Q16832	Discoidin domain-containing receptor 2 OS=Homo sapiens GN=DDR2 PE=1 SV=2 - [DDR2_HUMAN]	0,028	1,26	0,022	0,006
Q9UPQ0-10	Isoform 10 of LIM and calponin homology domains-containing protein 1 OS=Homo sapiens GN=LIMCH1 - [LIMC1_HUMAN]	0,028	1,31	0,015	0,013
Q8NBF2	NHL repeat-containing protein 2 OS=Homo sapiens GN=NHLRC2 PE=1 SV=1 - [NHLC2_HUMAN]	0,028	1,15	0,021	0,007
P02461	Collagen alpha-1(III) chain OS=Homo sapiens GN=COL3A1 PE=1 SV=4 - [CO3A1_HUMAN]	0,028	1,48	0,024	0,004
O75110-2	Isoform Short of Probable phospholipid-transporting ATPase IIA OS=Homo sapiens GN=ATP9A - [ATP9A_HUMAN]	0,028	1,35	0,017	0,011
Q9BUP0	EF-hand domain-containing protein D1 OS=Homo sapiens GN=EFHD1 PE=1 SV=1 - [EFHD1_HUMAN]	0,028	1,39	0,019	0,009
O95834	Echinoderm microtubule-associated protein-like 2 OS=Homo sapiens GN=EML2 PE=1 SV=1 - [EMAL2_HUMAN]	0,028	1,31	0,011	0,016
Q9H6U6-6	Isoform 6 of Breast carcinoma-amplified sequence 3 OS=Homo sapiens GN=BCAS3 - [BCAS3_HUMAN]	0,028	1,22	0,017	0,011
P11142	Heat shock cognate 71 kDa protein OS=Homo sapiens GN=HSPA8 PE=1 SV=1 - [HSP7C_HUMAN]	0,028	1,07	0,011	0,017
P08174-4	Isoform 4 of Complement decay-accelerating factor OS=Homo sapiens GN=CD55 - [DAF_HUMAN]	0,028	1,34	0,013	0,014
P34096	Ribonuclease 4 OS=Homo sapiens GN=RNASE4 PE=1 SV=3 - [RNAS4_HUMAN]	0,028	1,45	0,017	0,010
Q8IWZ3	Ankyrin repeat and KH domain-containing protein 1 OS=Homo sapiens GN=ANKHD1 PE=1 SV=1 - [ANKH1_HUMAN]	0,028	1,23	0,026	0,001
Q13563-5	Isoform 5 of Polycystin-2 OS=Homo sapiens GN=PKD2 - [PKD2_HUMAN]	0,028	1,28	0,012	0,016
Q9NVE7	Pantothenate kinase 4 OS=Homo sapiens GN=PANK4 PE=1 SV=1 - [PANK4_HUMAN]	0,028	1,23	0,019	0,009
Q13439-3	Isoform 3 of Golgin subfamily A member 4 OS=Homo sapiens GN=GOLGA4 - [GOGA4_HUMAN]	0,028	1,19	0,019	0,009
Q7Z304	MAM domain-containing protein 2 OS=Homo sapiens GN=MAMDC2 PE=2 SV=3 - [MAMC2_HUMAN]	0,028	1,55	0,025	0,003
P09936	Ubiquitin carboxyl-terminal hydrolase isozyme L1 OS=Homo sapiens GN=UCHL1 PE=1 SV=2 -	0,028	1,56	0,016	0,011

	[UCHL1_HUMAN]				
P07311	Acylphosphatase-1 OS=Homo sapiens GN=ACYP1 PE=1 SV=2 - [ACYP1_HUMAN]	0,028	1,34	0,013	0,015
P53602	Diphosphomevalonate decarboxylase OS=Homo sapiens GN=MVD PE=1 SV=1 - [MVD1_HUMAN]	0,028	1,16	0,025	0,003
Q96RW7-2	Isoform 2 of Hemicentin-1 OS=Homo sapiens GN=HMCN1 - [HMCN1_HUMAN]	0,027	1,43	0,023	0,005
P40222	Alpha-taxilin OS=Homo sapiens GN=TXLNA PE=1 SV=3 - [TXLNA_HUMAN]	0,027	1,10	0,019	0,008
Q12765	Secernin-1 OS=Homo sapiens GN=SCRN1 PE=1 SV=2 - [SCRN1_HUMAN]	0,027	1,15	0,017	0,010
Q13620-3	Isoform 3 of Cullin-4B OS=Homo sapiens GN=CUL4B - [CUL4B_HUMAN]	0,027	1,20	0,020	0,008
P45974-2	Isoform Short of Ubiquitin carboxyl-terminal hydrolase 5 OS=Homo sapiens GN=USP5 - [UBP5_HUMAN]	0,027	1,13	0,017	0,011
O43707	Alpha-actinin-4 OS=Homo sapiens GN=ACTN4 PE=1 SV=2 - [ACTN4_HUMAN]	0,027	1,19	0,016	0,011
Q07954	Prolow-density lipoprotein receptor-related protein 1 OS=Homo sapiens GN=LRP1 PE=1 SV=2 - [LRP1_HUMAN]	0,027	1,18	0,024	0,003
P07737	Profilin-1 OS=Homo sapiens GN=PFN1 PE=1 SV=2 - [PROF1_HUMAN]	0,027	1,09	0,019	0,008
P61020	Ras-related protein Rab-5B OS=Homo sapiens GN=RAB5B PE=1 SV=1 - [RAB5B_HUMAN]	0,027	1,22	0,022	0,005
Q9Y2B9	cAMP-dependent protein kinase inhibitor gamma OS=Homo sapiens GN=PKIG PE=2 SV=1 - [IPKG_HUMAN]	0,027	1,35	0,024	0,003
P19105	Myosin regulatory light chain 12A OS=Homo sapiens GN=MYL12A PE=1 SV=2 - [ML12A_HUMAN]	0,027	1,23	0,013	0,014
Q9NRX5	Serine incorporator 1 OS=Homo sapiens GN=SERINC1 PE=1 SV=1 - [SERC1_HUMAN]	0,027	1,21	0,016	0,011
P16112-3	Isoform 3 of Aggrecan core protein OS=Homo sapiens GN=ACAN - [PGCA_HUMAN]	0,027	1,75	0,023	0,004
Q32MZ4-4	Isoform 4 of Leucine-rich repeat flightless-interacting protein 1 OS=Homo sapiens GN=LRRFIP1 - [LRRF1_HUMAN]	0,027	1,23	0,013	0,014
O75116	Rho-associated protein kinase 2 OS=Homo sapiens GN=ROCK2 PE=1 SV=4 - [ROCK2_HUMAN]	0,027	1,23	0,012	0,015
Q15843	NEDD8 OS=Homo sapiens GN=NEDD8 PE=1 SV=1 - [NEDD8_HUMAN]	0,027	1,17	0,025	0,002
Q4G0N4-2	Isoform 2 of NAD kinase 2, mitochondrial OS=Homo sapiens GN=NADK2 - [NAKD2_HUMAN]	0,027	1,15	0,023	0,003
Q9UBT7-3	Isoform 3 of Alpha-catulin OS=Homo sapiens GN=CTNNAL1 - [CTNL1_HUMAN]	0,027	1,74	0,017	0,010
Q9UBC2-3	Isoform 3 of Epidermal growth factor receptor substrate 15-like 1 OS=Homo sapiens GN=EPS15L1 - [EP15R_HUMAN]	0,027	1,09	0,027	0,000
Q8N6G6	ADAMTS-like protein 1 OS=Homo sapiens GN=ADAMTSL1 PE=1 SV=4 - [ATL1_HUMAN]	0,027	1,46	0,010	0,017
Q8TET4	Neutral alpha-glucosidase C OS=Homo sapiens GN=GANC PE=2 SV=3 - [GANC_HUMAN]	0,027	1,36	0,015	0,012
O60888-3	Isoform C of Protein CutA OS=Homo sapiens GN=CUTA - [CUTA_HUMAN]	0,027	1,37	0,014	0,012
P46934-4	Isoform 4 of E3 ubiquitin-protein ligase NEDD4 OS=Homo sapiens GN=NEDD4 - [NEDD4_HUMAN]	0,027	1,34	0,009	0,018
Q00613-2	Isoform Short of Heat shock factor protein 1 OS=Homo sapiens GN=HSF1 - [HSF1_HUMAN]	0,027	1,13	0,025	0,002
Q15121	Astrocytic phosphoprotein PEA-15 OS=Homo sapiens GN=PEA15 PE=1 SV=2 - [PEA15_HUMAN]	0,027	1,21	0,023	0,004
Q53LP3	Ankyrin repeat domain-containing protein SOWAHC OS=Homo sapiens GN=SOWAHC PE=1 SV=1 - [SWAHC_HUMAN]	0,027	1,18	0,020	0,007
Q9UNE7-2	Isoform 2 of E3 ubiquitin-protein ligase CHIP OS=Homo sapiens GN=STUB1 - [CHIP_HUMAN]	0,027	1,27	0,015	0,011
Q8N573-8	Isoform 8 of Oxidation resistance protein 1 OS=Homo sapiens GN=OXR1 - [OXR1_HUMAN]	0,027	1,24	0,019	0,008
P48960-2	Isoform 2 of CD97 antigen OS=Homo sapiens GN=CD97 - [CD97_HUMAN]	0,027	1,52	0,026	0,000
Q9P2K6	Kelch-like protein 42 OS=Homo sapiens GN=KLHL42 PE=1 SV=2 - [KLH42_HUMAN]	0,027	1,62	0,015	0,012
Q14289-2	Isoform 2 of Protein-tyrosine kinase 2-beta OS=Homo sapiens GN=PTK2B - [FAK2_HUMAN]	0,027	1,34	0,013	0,014

Q9UBP9	PTB domain-containing engulfment adapter protein 1 OS=Homo sapiens GN=GULP1 PE=1 SV=1 - [GULP1_HUMAN]	0,027	1,27	0,016	0,010
Q9NQ48-3	Isoform 3 of Leucine zipper transcription factor-like protein 1 OS=Homo sapiens GN=LZTFL1 - [LZTL1_HUMAN]	0,026	1,16	0,015	0,012
O95861-4	Isoform 4 of 3'(2'),5'-bisphosphate nucleotidase 1 OS=Homo sapiens GN=BPNT1 - [BPNT1_HUMAN]	0,026	1,09	0,017	0,010
Q9H3U1-2	Isoform 2 of Protein unc-45 homolog A OS=Homo sapiens GN=UNC45A - [UN45A_HUMAN]	0,026	1,17	0,017	0,009
Q7L7X3-3	Isoform 3 of Serine/threonine-protein kinase TAO1 OS=Homo sapiens GN=TAOK1 - [TAOK1_HUMAN]	0,026	1,09	0,015	0,012
O75628	GTP-binding protein REM 1 OS=Homo sapiens GN=REM1 PE=1 SV=2 - [REM1_HUMAN]	0,026	1,46	0,014	0,013
Q16513-2	Isoform 2 of Serine/threonine-protein kinase N2 OS=Homo sapiens GN=PKN2 - [PKN2_HUMAN]	0,026	1,22	0,014	0,012
Q08431	Lactadherin OS=Homo sapiens GN=MFGE8 PE=1 SV=2 - [MFGM_HUMAN]	0,026	1,48	0,023	0,003
P52294	Importin subunit alpha-5 OS=Homo sapiens GN=KPNA1 PE=1 SV=3 - [IMA5_HUMAN]	0,026	1,17	0,022	0,004
Q9P2K5-3	Isoform 3 of Myelin expression factor 2 OS=Homo sapiens GN=MYEF2 - [MYEF2_HUMAN]	0,026	1,30	0,013	0,014
Q6ZMJ4-2	Isoform 2 of Interleukin-34 OS=Homo sapiens GN=IL34 - [IL34_HUMAN]	0,026	1,43	0,014	0,012
Q9BUK6-5	Isoform 5 of Protein misato homolog 1 OS=Homo sapiens GN=MSTO1 - [MSTO1_HUMAN]	0,026	1,17	0,026	0,000
Q96EC8	Protein YIPF6 OS=Homo sapiens GN=YIPF6 PE=1 SV=2 - [YIPF6_HUMAN]	0,026	1,18	0,025	0,002
O60551	Glycylpeptide N-tetradecanoyltransferase 2 OS=Homo sapiens GN=NMT2 PE=1 SV=1 - [NMT2_HUMAN]	0,026	1,15	0,021	0,005
Q9Y266	Nuclear migration protein nudC OS=Homo sapiens GN=NUDC PE=1 SV=1 - [NUDC_HUMAN]	0,026	1,10	0,017	0,009
Q9BV57	1,2-dihydroxy-3-keto-5-methylthiopentene dioxygenase OS=Homo sapiens GN=ADI1 PE=1 SV=1 - [MTND_HUMAN]	0,026	1,29	0,019	0,007
P51911	Calponin-1 OS=Homo sapiens GN=CNN1 PE=1 SV=2 - [CNN1_HUMAN]	0,026	1,49	0,014	0,012
Q9NZN3	EH domain-containing protein 3 OS=Homo sapiens GN=EHD3 PE=1 SV=2 - [EHD3_HUMAN]	0,026	1,45	0,013	0,013
Q9UMX5	Neudesin OS=Homo sapiens GN=NENF PE=1 SV=1 - [NENF_HUMAN]	0,026	1,20	0,014	0,012
A6NIH7	Protein unc-119 homolog B OS=Homo sapiens GN=UNC119B PE=1 SV=1 - [U119B_HUMAN]	0,026	1,15	0,021	0,004
Q86YT6	E3 ubiquitin-protein ligase MIB1 OS=Homo sapiens GN=MIB1 PE=1 SV=1 - [MIB1_HUMAN]	0,026	1,16	0,022	0,004
Q53SF7-4	Isoform 4 of Cordon-bleu protein-like 1 OS=Homo sapiens GN=COBLL1 - [COBL1_HUMAN]	0,026	1,18	0,010	0,016
Q8N6Y2	Leucine-rich repeat-containing protein 17 OS=Homo sapiens GN=LRRC17 PE=2 SV=1 - [LRC17_HUMAN]	0,026	1,50	0,017	0,009
P13591-4	Isoform 4 of Neural cell adhesion molecule 1 OS=Homo sapiens GN=NCAM1 - [NCAM1_HUMAN]	0,026	2,37	0,024	0,002
P24844	Myosin regulatory light polypeptide 9 OS=Homo sapiens GN=MYL9 PE=1 SV=4 - [MYL9_HUMAN]	0,026	1,37	0,014	0,012
P09471-2	Isoform Alpha-2 of Guanine nucleotide-binding protein G(o) subunit alpha OS=Homo sapiens GN=GNAO1 - [GNAO_HUMAN]	0,026	1,85	0,014	0,011
Q15642-2	Isoform 2 of Cdc42-interacting protein 4 OS=Homo sapiens GN=TRIP10 - [CIP4_HUMAN]	0,026	1,27	0,017	0,009
O75578-3	Isoform 3 of Integrin alpha-10 OS=Homo sapiens GN=ITGA10 - [ITA10_HUMAN]	0,026	1,40	0,018	0,007
P08133	Annexin A6 OS=Homo sapiens GN=ANXA6 PE=1 SV=3 - [ANXA6_HUMAN]	0,026	1,20	0,016	0,010
P09486	SPARC OS=Homo sapiens GN=SPARC PE=1 SV=1 - [SPRC_HUMAN]	0,026	1,40	0,020	0,006
P22061	Protein-L-isoaspartate(D-aspartate) O-methyltransferase OS=Homo sapiens GN=PCMT1 PE=1 SV=4 - [PIMT_HUMAN]	0,026	1,41	0,021	0,005
O43237-2	Isoform 2 of Cytoplasmic dynein 1 light intermediate chain 2 OS=Homo sapiens GN=DYNC1LI2 - [DC1L2_HUMAN]	0,026	1,06	0,022	0,003

P55083	Microfibril-associated glycoprotein 4 OS=Homo sapiens GN=MFAP4 PE=1 SV=2 - [MFAP4_HUMAN]	0,026	1,86	0,016	0,010
Q96EQ0	Small glutamine-rich tetratricopeptide repeat-containing protein beta OS=Homo sapiens GN=SGTB PE=1 SV=1 - [SGTB_HUMAN]	0,026	1,20	0,013	0,013
Q14714	Sarcospan OS=Homo sapiens GN=SSPN PE=2 SV=3 - [SSPN_HUMAN]	0,026	1,52	0,021	0,004
Q6UXI9	Nephronectin OS=Homo sapiens GN=NPNT PE=2 SV=3 - [NPNT_HUMAN]	0,026	1,73	0,019	0,006
Q9UNF0-2	Isoform 2 of Protein kinase C and casein kinase substrate in neurons protein 2 OS=Homo sapiens GN=PACSIN2 - [PACN2_HUMAN]	0,026	1,15	0,019	0,006
O15498	Synaptobrevin homolog YKT6 OS=Homo sapiens GN=YKT6 PE=1 SV=1 - [YKT6_HUMAN]	0,025	1,13	0,016	0,010
Q14642	Type I inositol 1,4,5-trisphosphate 5-phosphatase OS=Homo sapiens GN=INPP5A PE=1 SV=1 - [I5P1_HUMAN]	0,025	1,25	0,014	0,012
Q86UU1-3	Isoform 3 of Pleckstrin homology-like domain family B member 1 OS=Homo sapiens GN=PHLDB1 - [PHLB1_HUMAN]	0,025	1,22	0,016	0,009
P11216	Glycogen phosphorylase, brain form OS=Homo sapiens GN=PYGB PE=1 SV=5 - [PYGB_HUMAN]	0,025	1,32	0,025	0,001
Q96DR7	Rho guanine nucleotide exchange factor 26 OS=Homo sapiens GN=ARHGEF26 PE=1 SV=4 - [ARHGQ_HUMAN]	0,025	1,35	0,014	0,012
P08572	Collagen alpha-2(IV) chain OS=Homo sapiens GN=COL4A2 PE=1 SV=4 - [CO4A2_HUMAN]	0,025	1,28	0,021	0,004
P36871	Phosphoglucomutase-1 OS=Homo sapiens GN=PGM1 PE=1 SV=3 - [PGM1_HUMAN]	0,025	1,34	0,016	0,009
Q6IQ22	Ras-related protein Rab-12 OS=Homo sapiens GN=RAB12 PE=1 SV=3 - [RAB12_HUMAN]	0,025	1,27	0,007	0,018
O95926-2	Isoform 2 of Pre-mRNA-splicing factor SYF2 OS=Homo sapiens GN=SYF2 - [SYF2_HUMAN]	0,025	1,28	0,016	0,009
P30626-3	Isoform 3 of Sorcin OS=Homo sapiens GN=SRI - [SORCN_HUMAN]	0,025	1,19	0,019	0,007
P30153	Serine/threonine-protein phosphatase 2A 65 kDa regulatory subunit A alpha isoform OS=Homo sapiens GN=PPP2R1A PE=1 SV=4 - [2AAA_HUMAN]	0,025	1,13	0,017	0,008
O60237	Protein phosphatase 1 regulatory subunit 12B OS=Homo sapiens GN=PPP1R12B PE=1 SV=2 - [MYPT2_HUMAN]	0,025	1,27	0,012	0,013
Q6ZN30-2	Isoform 2 of Zinc finger protein basonuclin-2 OS=Homo sapiens GN=BNC2 - [BNC2_HUMAN]	0,025	1,47	0,017	0,009
O75157-2	Isoform 2 of TSC22 domain family protein 2 OS=Homo sapiens GN=TSC22D2 - [T22D2_HUMAN]	0,025	1,26	0,024	0,001
Q9UJ04	Testis-specific Y-encoded-like protein 4 OS=Homo sapiens GN=TSPYL4 PE=1 SV=2 - [TSYL4_HUMAN]	0,025	1,15	0,015	0,010
P40123	Adenylyl cyclase-associated protein 2 OS=Homo sapiens GN=CAP2 PE=1 SV=1 - [CAP2_HUMAN]	0,025	1,24	0,012	0,013
P50238	Cysteine-rich protein 1 OS=Homo sapiens GN=CRIP1 PE=1 SV=3 - [CRIP1_HUMAN]	0,025	1,58	0,018	0,007
P40763-3	Isoform 3 of Signal transducer and activator of transcription 3 OS=Homo sapiens GN=STAT3 - [STAT3_HUMAN]	0,025	1,14	0,023	0,002
Q9UM47	Neurogenic locus notch homolog protein 3 OS=Homo sapiens GN=NOTCH3 PE=1 SV=2 - [NOTC3_HUMAN]	0,025	1,37	0,012	0,013
O75475	PC4 and SFRS1-interacting protein OS=Homo sapiens GN=PSIP1 PE=1 SV=1 - [PSIP1_HUMAN]	0,025	1,36	0,014	0,011
Q96JY6-4	Isoform 4 of PDZ and LIM domain protein 2 OS=Homo sapiens GN=PDLIM2 - [PDLI2_HUMAN]	0,025	1,17	0,018	0,007
Q15404	Ras suppressor protein 1 OS=Homo sapiens GN=RSU1 PE=1 SV=3 - [RSU1_HUMAN]	0,025	1,28	0,014	0,011
Q99497	Protein deglycase DJ-1 OS=Homo sapiens GN=PARK7 PE=1 SV=2 - [PARK7_HUMAN]	0,025	1,18	0,019	0,006
P43034	Platelet-activating factor acetylhydrolase IB subunit alpha OS=Homo sapiens GN=PAFAH1B1 PE=1 SV=2 - [LIS1_HUMAN]	0,025	1,18	0,020	0,005
Q8NCH0	Carbohydrate sulfotransferase 14 OS=Homo sapiens GN=CHST14 PE=1 SV=2 - [CHSTE_HUMAN]	0,025	1,67	0,022	0,002
Q96ST3	Paired amphipathic helix protein Sin3a OS=Homo sapiens GN=SIN3A PE=1 SV=2 - [SIN3A_HUMAN]	0,025	1,12	0,018	0,007

Q99436-2	Isoform 2 of Proteasome subunit beta type-7 OS=Homo sapiens GN=PSMB7 - [PSB7_HUMAN]	0,025	1,24	0,014	0,011
P13611-5	Isoform Vint of Versican core protein OS=Homo sapiens GN=VCAN - [CSPG2_HUMAN]	0,025	1,97	0,023	0,002
P13797	Plastin-3 OS=Homo sapiens GN=PLS3 PE=1 SV=4 - [PLST_HUMAN]	0,025	1,30	0,019	0,006
Q9Y281-3	Isoform 3 of Cofilin-2 OS=Homo sapiens GN=CFL2 - [COF2_HUMAN]	0,025	1,32	0,016	0,009
Q6NZY7	Cdc42 effector protein 5 OS=Homo sapiens GN=CDC42EP5 PE=2 SV=1 - [BORG3_HUMAN]	0,025	1,19	0,022	0,003
Q9UBP4	Dickkopf-related protein 3 OS=Homo sapiens GN=DKK3 PE=1 SV=2 - [DKK3_HUMAN]	0,025	1,84	0,018	0,007
O43813	LanC-like protein 1 OS=Homo sapiens GN=LANCL1 PE=1 SV=1 - [LANC1_HUMAN]	0,025	1,22	0,014	0,011
Q96I34	Protein phosphatase 1 regulatory subunit 16A OS=Homo sapiens GN=PPP1R16A PE=1 SV=1 - [PP16A_HUMAN]	0,025	1,22	0,011	0,014
Q9NSY0	Nuclear receptor-binding protein 2 OS=Homo sapiens GN=NRBP2 PE=2 SV=2 - [NRBP2_HUMAN]	0,025	1,22	0,014	0,010
O14733	Dual specificity mitogen-activated protein kinase kinase 7 OS=Homo sapiens GN=MAP2K7 PE=1 SV=2 - [MP2K7_HUMAN]	0,025	1,13	0,022	0,003
Q9C0E8-2	Isoform 2 of Protein lunapark OS=Homo sapiens GN=LNP - [LNP_HUMAN]	0,025	1,23	0,013	0,011
P60981	Destrin OS=Homo sapiens GN=DSTN PE=1 SV=3 - [DEST_HUMAN]	0,025	1,28	0,020	0,004
P48059-3	Isoform 3 of LIM and senescent cell antigen-like-containing domain protein 1 OS=Homo sapiens GN=LIMS1 - [LIMS1_HUMAN]	0,025	1,29	0,007	0,018
P55196	Afadin OS=Homo sapiens GN=MLLT4 PE=1 SV=3 - [AFAD_HUMAN]	0,025	1,18	0,012	0,013
Q16851	UTP--glucose-1-phosphate uridylyltransferase OS=Homo sapiens GN=UGP2 PE=1 SV=5 - [UGPA_HUMAN]	0,025	1,16	0,021	0,004
Q9NZM3-4	Isoform 4 of Intersectin-2 OS=Homo sapiens GN=ITSN2 - [ITSN2_HUMAN]	0,025	1,18	0,017	0,008
Q9BY44-3	Isoform 3 of Eukaryotic translation initiation factor 2A OS=Homo sapiens GN=EIF2A - [EIF2A_HUMAN]	0,025	1,20	0,023	0,001
Q8N1G4	Leucine-rich repeat-containing protein 47 OS=Homo sapiens GN=LRRC47 PE=1 SV=1 - [LRC47_HUMAN]	0,025	1,15	0,019	0,006
Q9BZG1-4	Isoform 4 of Ras-related protein Rab-34 OS=Homo sapiens GN=RAB34 - [RAB34_HUMAN]	0,025	1,23	0,019	0,006
Q13976-2	Isoform Beta of cGMP-dependent protein kinase 1 OS=Homo sapiens GN=PRKG1 - [KGP1_HUMAN]	0,025	1,25	0,020	0,005
Q6AZY7-2	Isoform 2 of Scavenger receptor class A member 3 OS=Homo sapiens GN=SCARA3 - [SCAR3_HUMAN]	0,025	1,54	0,019	0,006
P20336	Ras-related protein Rab-3A OS=Homo sapiens GN=RAB3A PE=1 SV=1 - [RAB3A_HUMAN]	0,025	1,24	0,010	0,015
Q8WUY3-4	Isoform 4 of Protein prune homolog 2 OS=Homo sapiens GN=PRUNE2 - [PRUN2_HUMAN]	0,025	1,39	0,015	0,010
Q6NW29	RWD domain-containing protein 4 OS=Homo sapiens GN=RWDD4 PE=1 SV=3 - [RWDD4_HUMAN]	0,025	1,14	0,024	0,001
P51178	1-phosphatidylinositol 4,5-bisphosphate phosphodiesterase delta-1 OS=Homo sapiens GN=PLCD1 PE=1 SV=2 - [PLCD1_HUMAN]	0,025	1,17	0,023	0,002
Q5JR59	Microtubule-associated tumor suppressor candidate 2 OS=Homo sapiens GN=MTUS2 PE=1 SV=3 - [MTUS2_HUMAN]	0,025	1,43	0,011	0,013
P04792	Heat shock protein beta-1 OS=Homo sapiens GN=HSPB1 PE=1 SV=2 - [HSPB1_HUMAN]	0,024	1,27	0,015	0,009
Q9HD33-3	Isoform 3 of 39S ribosomal protein L47, mitochondrial OS=Homo sapiens GN=MRPL47 - [RM47_HUMAN]	0,024	1,18	0,020	0,004
Q9Y625	Glypican-6 OS=Homo sapiens GN=GPC6 PE=1 SV=1 - [GPC6_HUMAN]	0,024	1,75	0,016	0,008
P19022-2	Isoform 2 of Cadherin-2 OS=Homo sapiens GN=CDH2 - [CADH2_HUMAN]	0,024	1,35	0,021	0,003
Q93063	Exostosin-2 OS=Homo sapiens GN=EXT2 PE=1 SV=1 - [EXT2_HUMAN]	0,024	1,70	0,015	0,009
P02511	Alpha-crystallin B chain OS=Homo sapiens GN=CRYAB PE=1 SV=2 - [CRYAB_HUMAN]	0,024	1,38	0,024	0,000

P12429	Annexin A3 OS=Homo sapiens GN=ANXA3 PE=1 SV=3 - [ANXA3_HUMAN]	0,024	1,91	0,013	0,011
P53420	Collagen alpha-4(IV) chain OS=Homo sapiens GN=COL4A4 PE=1 SV=3 - [CO4A4_HUMAN]	0,024	1,54	0,022	0,002
Q4KMQ2-3	Isoform 3 of Anoctamin-6 OS=Homo sapiens GN=ANO6 - [ANO6_HUMAN]	0,024	1,26	0,013	0,011
P05230	Fibroblast growth factor 1 OS=Homo sapiens GN=FGF1 PE=1 SV=1 - [FGF1_HUMAN]	0,024	1,29	0,011	0,013
O75368	SH3 domain-binding glutamic acid-rich-like protein OS=Homo sapiens GN=SH3BGRL PE=1 SV=1 - [SH3L1_HUMAN]	0,024	1,20	0,014	0,011
Q15020-4	Isoform 4 of Squamous cell carcinoma antigen recognized by T-cells 3 OS=Homo sapiens GN=SART3 - [SART3_HUMAN]	0,024	1,26	0,018	0,006
O94832	Unconventional myosin-Id OS=Homo sapiens GN=MYO1D PE=1 SV=2 - [MYO1D_HUMAN]	0,024	1,24	0,019	0,006
O15230	Laminin subunit alpha-5 OS=Homo sapiens GN=LAMA5 PE=1 SV=8 - [LAMA5_HUMAN]	0,024	1,23	0,016	0,008
Q96JQ0	Protocadherin-16 OS=Homo sapiens GN=DCHS1 PE=1 SV=1 - [PCD16_HUMAN]	0,024	1,45	0,017	0,007
Q9UGI8-2	Isoform 2 of Testin OS=Homo sapiens GN=TES - [TES_HUMAN]	0,024	1,40	0,015	0,009
O60271-4	Isoform 4 of C-Jun-amino-terminal kinase-interacting protein 4 OS=Homo sapiens GN=SPAG9 - [JIP4_HUMAN]	0,024	1,15	0,018	0,006
P23352	Anosmin-1 OS=Homo sapiens GN=ANOS1 PE=1 SV=3 - [KALM_HUMAN]	0,024	1,34	0,016	0,008
Q75T13-2	Isoform 2 of GPI inositol-deacylase OS=Homo sapiens GN=PGAP1 - [PGAP1_HUMAN]	0,024	1,16	0,022	0,002
Q5JRA6-2	Isoform 2 of Melanoma inhibitory activity protein 3 OS=Homo sapiens GN=MIA3 - [MIA3_HUMAN]	0,024	1,05	0,019	0,005
Q9Y2G5-2	Isoform B of GDP-fucose protein O-fucosyltransferase 2 OS=Homo sapiens GN=POFUT2 - [OFUT2_HUMAN]	0,024	1,19	0,019	0,005
Q9P227-2	Isoform 2 of Rho GTPase-activating protein 23 OS=Homo sapiens GN=ARHGAP23 - [RHG23_HUMAN]	0,024	1,17	0,020	0,004
O43294-2	Isoform 2 of Transforming growth factor beta-1-induced transcript 1 protein OS=Homo sapiens GN=TGFB1I1 - [TGFI1_HUMAN]	0,024	1,17	0,017	0,007
O75970-5	Isoform 4 of Multiple PDZ domain protein OS=Homo sapiens GN=MPDZ - [MPDZ_HUMAN]	0,024	1,17	0,013	0,010
Q8IW45-4	Isoform 4 of ATP-dependent (S)-NAD(P)H-hydrate dehydratase OS=Homo sapiens GN=CARKD - [NNRD_HUMAN]	0,024	1,34	0,018	0,006
P62847-2	Isoform 2 of 40S ribosomal protein S24 OS=Homo sapiens GN=RPS24 - [RS24_HUMAN]	0,024	1,46	0,020	0,004
Q9H3U7	SPARC-related modular calcium-binding protein 2 OS=Homo sapiens GN=SMOC2 PE=2 SV=2 - [SMOC2_HUMAN]	0,024	1,30	0,020	0,003
Q96FW1	Ubiquitin thioesterase OTUB1 OS=Homo sapiens GN=OTUB1 PE=1 SV=2 - [OTUB1_HUMAN]	0,024	1,15	0,020	0,003
Q9H082	Ras-related protein Rab-33B OS=Homo sapiens GN=RAB33B PE=1 SV=1 - [RB33B_HUMAN]	0,024	1,19	0,012	0,012
Q7L0Y3	Mitochondrial ribonuclease P protein 1 OS=Homo sapiens GN=TRMT10C PE=1 SV=2 - [MRRP1_HUMAN]	0,023	1,49	0,020	0,004
O14495	Phospholipid phosphatase 3 OS=Homo sapiens GN=PLPP3 PE=1 SV=1 - [PLPP3_HUMAN]	0,023	1,34	0,017	0,006
Q6GMV3	Putative peptidyl-tRNA hydrolase PTRHD1 OS=Homo sapiens GN=PTRHD1 PE=1 SV=1 - [PTRD1_HUMAN]	0,023	1,17	0,020	0,003
Q8WW12-2	Isoform 2 of PEST proteolytic signal-containing nuclear protein OS=Homo sapiens GN=PCNP - [PCNP_HUMAN]	0,023	1,20	0,019	0,005
O95359-3	Isoform 3 of Transforming acidic coiled-coil-containing protein 2 OS=Homo sapiens GN=TACC2 - [TACC2_HUMAN]	0,023	1,28	0,015	0,008
Q9NTK5-2	Isoform 2 of Obg-like ATPase 1 OS=Homo sapiens GN=OLA1 - [OLA1_HUMAN]	0,023	1,14	0,023	0,000
P55011-3	Isoform 2 of Solute carrier family 12 member 2 OS=Homo sapiens GN=SLC12A2 - [S12A2_HUMAN]	0,023	1,20	0,016	0,007
Q7Z3D6-5	Isoform 5 of UPF0317 protein C14orf159, mitochondrial OS=Homo sapiens GN=C14orf159 -	0,023	1,60	0,021	0,002

	[CN159_HUMAN]				
Q13425	Beta-2-syntrophin OS=Homo sapiens GN=SNTB2 PE=1 SV=1 - [SNTB2_HUMAN]	0,023	1,35	0,015	0,008
Q9P1Z2-4	Isoform 4 of Calcium-binding and coiled-coil domain-containing protein 1 OS=Homo sapiens GN=CALCOCO1 - [CACO1_HUMAN]	0,023	1,27	0,017	0,007
P63092-3	Isoform 3 of Guanine nucleotide-binding protein G(s) subunit alpha isoforms short OS=Homo sapiens GN=GNAS - [GNAS2_HUMAN]	0,023	1,23	0,019	0,004
Q8WX93-3	Isoform 3 of Palladin OS=Homo sapiens GN=PALLD - [PALLD_HUMAN]	0,023	1,25	0,014	0,009
P11413	Glucose-6-phosphate 1-dehydrogenase OS=Homo sapiens GN=G6PD PE=1 SV=4 - [G6PD_HUMAN]	0,023	1,12	0,020	0,003
O00233-2	Isoform p27-S of 26S proteasome non-ATPase regulatory subunit 9 OS=Homo sapiens GN=PSMD9 - [PSMD9_HUMAN]	0,023	1,09	0,019	0,004
P21926	CD9 antigen OS=Homo sapiens GN=CD9 PE=1 SV=4 - [CD9_HUMAN]	0,023	1,44	0,019	0,004
Q13642-1	Isoform 1 of Four and a half LIM domains protein 1 OS=Homo sapiens GN=FHL1 - [FHL1_HUMAN]	0,023	1,29	0,020	0,003
Q8WVM7-2	Isoform 2 of Cohesin subunit SA-1 OS=Homo sapiens GN=STAG1 - [STAG1_HUMAN]	0,023	1,15	0,022	0,001
Q5EBL4	RILP-like protein 1 OS=Homo sapiens GN=RILPL1 PE=1 SV=1 - [RIPL1_HUMAN]	0,023	1,25	0,021	0,002
Q8IVN8	Somatomedin-B and thrombospondin type-1 domain-containing protein OS=Homo sapiens GN=SBSPON PE=1 SV=2 - [SBSPO_HUMAN]	0,023	1,79	0,021	0,001
O00178	GTP-binding protein 1 OS=Homo sapiens GN=GTPBP1 PE=1 SV=3 - [GTPB1_HUMAN]	0,023	1,12	0,017	0,006
P30622-2	Isoform 3 of CAP-Gly domain-containing linker protein 1 OS=Homo sapiens GN=CLIP1 - [CLIP1_HUMAN]	0,023	1,11	0,023	0,000
Q9Y4D1-2	Isoform 2 of Disheveled-associated activator of morphogenesis 1 OS=Homo sapiens GN=DAAM1 - [DAAM1_HUMAN]	0,023	1,17	0,016	0,007
O95425-4	Isoform SV4 of Supervillin OS=Homo sapiens GN=SVIL - [SVIL_HUMAN]	0,023	1,27	0,017	0,005
O43175	D-3-phosphoglycerate dehydrogenase OS=Homo sapiens GN=PHGDH PE=1 SV=4 - [SERA_HUMAN]	0,023	1,38	0,022	0,000
P62736	Actin, aortic smooth muscle OS=Homo sapiens GN=ACTA2 PE=1 SV=1 - [ACTA_HUMAN]	0,023	1,24	0,018	0,004
P56199	Integrin alpha-1 OS=Homo sapiens GN=ITGA1 PE=1 SV=2 - [ITA1_HUMAN]	0,023	1,20	0,013	0,010
Q13418	Integrin-linked protein kinase OS=Homo sapiens GN=ILK PE=1 SV=2 - [ILK_HUMAN]	0,023	1,23	0,014	0,009
Q9ULV0	Unconventional myosin-Vb OS=Homo sapiens GN=MYO5B PE=1 SV=3 - [MYO5B_HUMAN]	0,023	1,52	0,017	0,006
O95630-2	Isoform 2 of STAM-binding protein OS=Homo sapiens GN=STAMBP - [STABP_HUMAN]	0,023	1,14	0,020	0,002
Q03001	Dystonin OS=Homo sapiens GN=DST PE=1 SV=4 - [DYST_HUMAN]	0,023	1,08	0,015	0,007
Q8N436	Inactive carboxypeptidase-like protein X2 OS=Homo sapiens GN=CPXM2 PE=2 SV=3 - [CPXM2_HUMAN]	0,022	1,39	0,015	0,007
P16035	Metalloproteinase inhibitor 2 OS=Homo sapiens GN=TIMP2 PE=1 SV=2 - [TIMP2_HUMAN]	0,022	1,42	0,018	0,004
Q9HBG6-11	Isoform 11 of Intraflagellar transport protein 122 homolog OS=Homo sapiens GN=IFT122 - [IF122_HUMAN]	0,022	1,22	0,018	0,004
P67775-2	Isoform 2 of Serine/threonine-protein phosphatase 2A catalytic subunit alpha isoform OS=Homo sapiens GN=PPP2CA - [PP2AA_HUMAN]	0,022	1,19	0,017	0,005
Q8IX30	Signal peptide, CUB and EGF-like domain-containing protein 3 OS=Homo sapiens GN=SCUBE3 PE=1 SV=1 - [SCUB3_HUMAN]	0,022	1,61	0,020	0,003
Q92625	Ankyrin repeat and SAM domain-containing protein 1A OS=Homo sapiens GN=ANKS1A PE=1 SV=4 - [ANS1A_HUMAN]	0,022	1,21	0,019	0,004
P80404	4-aminobutyrate aminotransferase, mitochondrial OS=Homo sapiens GN=ABAT PE=1 SV=3 - [GABT_HUMAN]	0,022	1,25	0,019	0,004

P18084	Integrin beta-5 OS=Homo sapiens GN=ITGB5 PE=1 SV=1 - [ITB5_HUMAN]	0,022	1,42	0,018	0,004
Q8IWV2	Contactin-4 OS=Homo sapiens GN=CNTN4 PE=1 SV=1 - [CNTN4_HUMAN]	0,022	1,41	0,020	0,002
O00151	PDZ and LIM domain protein 1 OS=Homo sapiens GN=PDLIM1 PE=1 SV=4 - [PDLI1_HUMAN]	0,022	1,33	0,016	0,006
Q8IXJ6-2	Isoform 2 of NAD-dependent protein deacetylase sirtuin-2 OS=Homo sapiens GN=SIRT2 - [SIR2_HUMAN]	0,022	1,10	0,021	0,001
P62805	Histone H4 OS=Homo sapiens GN=HIST1H4A PE=1 SV=2 - [H4_HUMAN]	0,022	1,16	0,020	0,002
Q99622	Protein C10 OS=Homo sapiens GN=C12orf57 PE=1 SV=1 - [C10_HUMAN]	0,022	1,27	0,021	0,001
P54750-3	Isoform 3 of Calcium/calmodulin-dependent 3',5'-cyclic nucleotide phosphodiesterase 1A OS=Homo sapiens GN=PDE1A - [PDE1A_HUMAN]	0,022	1,17	0,010	0,012
O60245	Protocadherin-7 OS=Homo sapiens GN=PCDH7 PE=1 SV=2 - [PCDH7_HUMAN]	0,022	1,29	0,020	0,002
P10909-4	Isoform 4 of Clusterin OS=Homo sapiens GN=CLU - [CLUS_HUMAN]	0,022	1,38	0,021	0,001
Q8WUM4	Programmed cell death 6-interacting protein OS=Homo sapiens GN=PDCD6IP PE=1 SV=1 - [PDC6I_HUMAN]	0,022	1,13	0,013	0,009
Q14643-4	Isoform 4 of Inositol 1,4,5-trisphosphate receptor type 1 OS=Homo sapiens GN=ITPR1 - [ITPR1_HUMAN]	0,022	1,19	0,019	0,003
O43150-2	Isoform 2 of Arf-GAP with SH3 domain, ANK repeat and PH domain-containing protein 2 OS=Homo sapiens GN=ASAP2 - [ASAP2_HUMAN]	0,022	1,18	0,016	0,006
Q96DH6-2	Isoform 2 of RNA-binding protein Musashi homolog 2 OS=Homo sapiens GN=MSI2 - [MSI2H_HUMAN]	0,022	1,26	0,016	0,006
O94851-2	Isoform 2 of Protein-methionine sulfoxide oxidase MICAL2 OS=Homo sapiens GN=MICAL2 - [MICA2_HUMAN]	0,022	1,17	0,020	0,002
O95631	Netrin-1 OS=Homo sapiens GN=NTN1 PE=1 SV=2 - [NET1_HUMAN]	0,022	1,73	0,014	0,008
Q86VS8	Protein Hook homolog 3 OS=Homo sapiens GN=HOOK3 PE=1 SV=2 - [HOOK3_HUMAN]	0,022	1,10	0,020	0,001
P04406-2	Isoform 2 of Glyceraldehyde-3-phosphate dehydrogenase OS=Homo sapiens GN=GAPDH - [G3P_HUMAN]	0,022	1,10	0,021	0,000
Q8N612	FTS and Hook-interacting protein OS=Homo sapiens GN=FAM160A2 PE=1 SV=3 - [F16A2_HUMAN]	0,022	1,14	0,021	0,001
Q9NUJ1	Mycophenolic acid acyl-glucuronide esterase, mitochondrial OS=Homo sapiens GN=ABHD10 PE=1 SV=1 - [ABHDA_HUMAN]	0,022	1,30	0,014	0,007
Q14203-3	Isoform 3 of Dynactin subunit 1 OS=Homo sapiens GN=DCTN1 - [DCTN1_HUMAN]	0,022	1,10	0,015	0,006
P53814-5	Isoform B2 of Smoothelin OS=Homo sapiens GN=SMTN - [SMTN_HUMAN]	0,022	1,40	0,017	0,005
P54725-2	Isoform 2 of UV excision repair protein RAD23 homolog A OS=Homo sapiens GN=RAD23A - [RD23A_HUMAN]	0,022	1,16	0,017	0,004
Q9HCB6	Spondin-1 OS=Homo sapiens GN=SPON1 PE=1 SV=2 - [SPON1_HUMAN]	0,022	1,81	0,013	0,008
P07437	Tubulin beta chain OS=Homo sapiens GN=TUBB PE=1 SV=2 - [TBB5_HUMAN]	0,022	1,06	0,019	0,002
Q9BZL4-3	Isoform 3 of Protein phosphatase 1 regulatory subunit 12C OS=Homo sapiens GN=PPP1R12C - [PP12C_HUMAN]	0,021	1,19	0,019	0,002
O43795-2	Isoform 2 of Unconventional myosin-Ib OS=Homo sapiens GN=MYO1B - [MYO1B_HUMAN]	0,021	1,16	0,013	0,009
Q12860-2	Isoform 2 of Contactin-1 OS=Homo sapiens GN=CNTN1 - [CNTN1_HUMAN]	0,021	1,84	0,018	0,004
Q9BR39	Junctophilin-2 OS=Homo sapiens GN=JPH2 PE=1 SV=2 - [JPH2_HUMAN]	0,021	1,19	0,020	0,001
O75688-5	Isoform 5 of Protein phosphatase 1B OS=Homo sapiens GN=PPM1B - [PPM1B_HUMAN]	0,021	1,27	0,021	0,000
Q9BSJ2-3	Isoform 2 of Gamma-tubulin complex component 2 OS=Homo sapiens GN=TUBGCP2 - [GCP2_HUMAN]	0,021	1,12	0,019	0,002

Q9Y608-2	Isoform 2 of Leucine-rich repeat flightless-interacting protein 2 OS=Homo sapiens GN=LRRFIP2 - [LRRF2_HUMAN]	0,021	1,17	0,016	0,005
Q9UBI6	Guanine nucleotide-binding protein G(I)/G(S)/G(O) subunit gamma-12 OS=Homo sapiens GN=GNG12 PE=1 SV=3 - [GBG12_HUMAN]	0,021	1,22	0,016	0,005
P07951	Tropomyosin beta chain OS=Homo sapiens GN=TPM2 PE=1 SV=1 - [TPM2_HUMAN]	0,021	1,45	0,017	0,004
O00186	Syntaxin-binding protein 3 OS=Homo sapiens GN=STXBP3 PE=1 SV=2 - [STXB3_HUMAN]	0,021	1,16	0,011	0,010
Q9NR46	Endophilin-B2 OS=Homo sapiens GN=SH3GLB2 PE=1 SV=1 - [SHLB2_HUMAN]	0,021	1,28	0,018	0,003
Q13618-3	Isoform 3 of Cullin-3 OS=Homo sapiens GN=CUL3 - [CUL3_HUMAN]	0,021	1,19	0,018	0,003
P29536-2	Isoform 2 of Leiomodin-1 OS=Homo sapiens GN=LMOD1 - [LMOD1_HUMAN]	0,021	1,30	0,017	0,004
Q16270	Insulin-like growth factor-binding protein 7 OS=Homo sapiens GN=IGFBP7 PE=1 SV=1 - [IBP7_HUMAN]	0,021	1,28	0,019	0,001
O95816	BAG family molecular chaperone regulator 2 OS=Homo sapiens GN=BAG2 PE=1 SV=1 - [BAG2_HUMAN]	0,021	1,28	0,019	0,002
Q8N283	Ankyrin repeat domain-containing protein 35 OS=Homo sapiens GN=ANKRD35 PE=2 SV=2 - [ANR35_HUMAN]	0,020	1,28	0,019	0,002
P11532-4	Isoform 3 of Dystrophin OS=Homo sapiens GN=DMD - [DMD_HUMAN]	0,020	1,23	0,018	0,003
Q6EEV4-2	Isoform 5 of DNA-directed RNA polymerase II subunit GRINL1A, isoforms 4/5 OS=Homo sapiens GN=POLR2M - [GL1AD_HUMAN]	0,020	1,16	0,019	0,001
Q9UIQ6-3	Isoform 3 of Leucyl-cystinyl aminopeptidase OS=Homo sapiens GN=LNPEP - [LCAP_HUMAN]	0,020	1,16	0,017	0,003
Q5T9L3-2	Isoform 2 of Protein wntless homolog OS=Homo sapiens GN=WLS - [WLS_HUMAN]	0,020	1,27	0,016	0,004
Q99523	Sortilin OS=Homo sapiens GN=SORT1 PE=1 SV=3 - [SORT_HUMAN]	0,020	1,21	0,018	0,002
O43639	Cytoplasmic protein NCK2 OS=Homo sapiens GN=NCK2 PE=1 SV=2 - [NCK2_HUMAN]	0,020	1,19	0,018	0,001
P21397	Amine oxidase [flavin-containing] A OS=Homo sapiens GN=MAOA PE=1 SV=1 - [AOFA_HUMAN]	0,020	1,17	0,017	0,003
Q63ZY3-3	Isoform 3 of KN motif and ankyrin repeat domain-containing protein 2 OS=Homo sapiens GN=KANK2 - [KANK2_HUMAN]	0,020	1,21	0,014	0,005
Q8N2S1-2	Isoform 2 of Latent-transforming growth factor beta-binding protein 4 OS=Homo sapiens GN=LTBP4 - [LTBP4_HUMAN]	0,019	1,40	0,013	0,006
Q15124	Phosphoglucomutase-like protein 5 OS=Homo sapiens GN=PGM5 PE=1 SV=2 - [PGM5_HUMAN]	0,019	1,37	0,018	0,001
P0DI83	Ras-related protein Rab-34, isoform NARR OS=Homo sapiens GN=RAB34 PE=1 SV=1 - [NARR_HUMAN]	0,019	1,12	0,011	0,008
O00401	Neural Wiskott-Aldrich syndrome protein OS=Homo sapiens GN=WASL PE=1 SV=2 - [WASL_HUMAN]	0,019	1,13	0,017	0,002
P32119	Peroxiredoxin-2 OS=Homo sapiens GN=PRDX2 PE=1 SV=5 - [PRDX2_HUMAN]	0,019	1,15	0,010	0,009
O60437	Periplakin OS=Homo sapiens GN=PPL PE=1 SV=4 - [PEPL_HUMAN]	0,019	1,33	0,017	0,002
P49023-2	Isoform Alpha of Paxillin OS=Homo sapiens GN=PXN - [PAXI_HUMAN]	0,019	1,15	0,013	0,006
P40818	Ubiquitin carboxyl-terminal hydrolase 8 OS=Homo sapiens GN=USP8 PE=1 SV=1 - [UBP8_HUMAN]	0,019	1,15	0,013	0,005
Q9UIC8	Leucine carboxyl methyltransferase 1 OS=Homo sapiens GN=LCMT1 PE=1 SV=2 - [LCMT1_HUMAN]	0,019	1,09	0,015	0,003
Q5BJF6-2	Isoform 2 of Outer dense fiber protein 2 OS=Homo sapiens GN=ODF2 - [ODFP2_HUMAN]	0,018	1,14	0,018	0,001
P50552	Vasodilator-stimulated phosphoprotein OS=Homo sapiens GN=VASP PE=1 SV=3 - [VASP_HUMAN]	0,018	1,09	0,015	0,003
P27348	14-3-3 protein theta OS=Homo sapiens GN=YWHAQ PE=1 SV=1 - [1433T_HUMAN]	0,018	1,12	0,017	0,002
P62070-3	Isoform 3 of Ras-related protein R-Ras2 OS=Homo sapiens GN=RRAS2 - [RRAS2_HUMAN]	0,018	1,25	0,016	0,002
Q96AC1	Fermitin family homolog 2 OS=Homo sapiens GN=FERMT2 PE=1 SV=1 - [FERM2_HUMAN]	0,018	1,18	0,018	0,000

Q9H2U2	Inorganic pyrophosphatase 2, mitochondrial OS=Homo sapiens GN=PPA2 PE=1 SV=2 - [IPYR2_HUMAN]	0,018	1,34	0,017	0,001
P61626	Lysozyme C OS=Homo sapiens GN=LYZ PE=1 SV=1 - [LYSC_HUMAN]	0,018	1,28	0,015	0,003
P05204	Non-histone chromosomal protein HMG-17 OS=Homo sapiens GN=HMGN2 PE=1 SV=3 - [HMGN2_HUMAN]	0,018	1,16	0,010	0,007
Q7Z2X4-3	Isoform 3 of PTB-containing, cubilin and LRP1-interacting protein OS=Homo sapiens GN=PID1 - [PCLI1_HUMAN]	0,018	1,30	0,015	0,003
Q16558	Calcium-activated potassium channel subunit beta-1 OS=Homo sapiens GN=KCNMB1 PE=1 SV=5 - [KCMB1_HUMAN]	0,018	1,23	0,017	0,000
Q15654	Thyroid receptor-interacting protein 6 OS=Homo sapiens GN=TRIP6 PE=1 SV=3 - [TRIP6_HUMAN]	0,018	1,27	0,016	0,002
Q92765	Secreted frizzled-related protein 3 OS=Homo sapiens GN=FRZB PE=1 SV=2 - [SFRP3_HUMAN]	0,017	1,47	0,016	0,001
P46108-2	Isoform Crk-I of Adapter molecule crk OS=Homo sapiens GN=CRK - [CRK_HUMAN]	0,017	1,09	0,015	0,002
Q03135	Caveolin-1 OS=Homo sapiens GN=CAV1 PE=1 SV=4 - [CAV1_HUMAN]	0,017	1,16	0,013	0,004
P25686-2	Isoform 2 of DnaJ homolog subfamily B member 2 OS=Homo sapiens GN=DNAJB2 - [DNJB2_HUMAN]	0,017	1,20	0,014	0,003
O95810	Serum deprivation-response protein OS=Homo sapiens GN=SDPR PE=1 SV=3 - [SDPR_HUMAN]	0,017	1,23	0,011	0,006
O60879-2	Isoform 2 of Protein diaphanous homolog 2 OS=Homo sapiens GN=DIAPH2 - [DIAP2_HUMAN]	0,016	1,12	0,015	0,000
P61353	60S ribosomal protein L27 OS=Homo sapiens GN=RPL27 PE=1 SV=2 - [RL27_HUMAN]	0,016	1,40	0,015	0,000
P26038	Moesin OS=Homo sapiens GN=MSN PE=1 SV=3 - [MOES_HUMAN]	0,015	1,05	0,013	0,003
Q9Y2A7	Nck-associated protein 1 OS=Homo sapiens GN=NCKAP1 PE=1 SV=1 - [NCKP1_HUMAN]	0,015	1,22	0,012	0,002
P28370-2	Isoform 2 of Probable global transcription activator SNF2L1 OS=Homo sapiens GN=SMARCA1 - [SMCA1_HUMAN]	0,012	1,10	0,011	0,001

Proteins downregulated in BAV dilated samples

Protein accession number	Protein name	Loading value [a]	Fold change	jack-knife (JK) confidence interval[b]	ABS (Loading value)-ABS(JK)[c]
Q9BXW7-2	Isoform 1 of Cat eye syndrome critical region protein 5 OS=Homo sapiens GN=CECR5 - [CECR5_HUMAN]	-0,038	0,47	0,020	0,018
Q96JG6	Syndetin OS=Homo sapiens GN=VPS50 PE=1 SV=3 - [VPS50_HUMAN]	-0,037	0,55	0,020	0,017
Q9H089	Large subunit GTPase 1 homolog OS=Homo sapiens GN=LSG1 PE=1 SV=2 - [LSG1_HUMAN]	-0,037	0,54	0,013	0,024
P18440	Arylamine N-acetyltransferase 1 OS=Homo sapiens GN=NAT1 PE=1 SV=2 - [ARY1_HUMAN]	-0,036	0,70	0,019	0,017
Q9NR30	Nucleolar RNA helicase 2 OS=Homo sapiens GN=DDX21 PE=1 SV=5 - [DDX21_HUMAN]	-0,035	0,80	0,022	0,013
P27695	DNA-(apurinic or apyrimidinic site) lyase OS=Homo sapiens GN=APEX1 PE=1 SV=2 - [APEX1_HUMAN]	-0,035	0,83	0,010	0,026
P22033	Methylmalonyl-CoA mutase, mitochondrial OS=Homo sapiens GN=MUT PE=1 SV=4 - [MUTA_HUMAN]	-0,035	0,69	0,015	0,020
Q00653-4	Isoform 4 of Nuclear factor NF-kappa-B p100 subunit OS=Homo sapiens GN=NFKB2 - [NFKB2_HUMAN]	-0,035	0,73	0,011	0,024
Q9H6T3-2	Isoform 2 of RNA polymerase II-associated protein 3 OS=Homo sapiens GN=RPAP3 - [RPAP3_HUMAN]	-0,034	0,75	0,022	0,012
Q16134-3	Isoform 2 of Electron transfer flavoprotein-ubiquinone oxidoreductase, mitochondrial OS=Homo	-0,034	0,77	0,030	0,004

	sapiens GN=ETFDH - [ETFD_HUMAN]				
P23246-2	Isoform Short of Splicing factor, proline- and glutamine-rich OS=Homo sapiens GN=SFPQ - [SFPQ_HUMAN]	-0,034	0,82	0,013	0,021
P05455	Lupus La protein OS=Homo sapiens GN=SSB PE=1 SV=2 - [LA_HUMAN]	-0,034	0,91	0,017	0,017
O75165	DnaJ homolog subfamily C member 13 OS=Homo sapiens GN=DNAJC13 PE=1 SV=5 - [DJC13_HUMAN]	-0,034	0,83	0,015	0,019
O43768	Alpha-endosulfine OS=Homo sapiens GN=ENSA PE=1 SV=1 - [ENSA_HUMAN]	-0,034	0,84	0,034	0,000
Q9P258	Protein RCC2 OS=Homo sapiens GN=RCC2 PE=1 SV=2 - [RCC2_HUMAN]	-0,034	0,43	0,011	0,022
Q01968-2	Isoform B of Inositol polyphosphate 5-phosphatase OCRL-1 OS=Homo sapiens GN=OCRL - [OCRL_HUMAN]	-0,034	0,79	0,015	0,019
Q13148	TAR DNA-binding protein 43 OS=Homo sapiens GN=TARDBP PE=1 SV=1 - [TADBP_HUMAN]	-0,034	0,82	0,017	0,016
O43353-2	Isoform 2 of Receptor-interacting serine/threonine-protein kinase 2 OS=Homo sapiens GN=RIPK2 - [RIPK2_HUMAN]	-0,034	0,71	0,020	0,014
Q9Y6D5	Brefeldin A-inhibited guanine nucleotide-exchange protein 2 OS=Homo sapiens GN=ARFGEF2 PE=1 SV=3 - [BIG2_HUMAN]	-0,034	0,81	0,015	0,019
Q9UGT4	Sushi domain-containing protein 2 OS=Homo sapiens GN=SUSD2 PE=1 SV=1 - [SUSD2_HUMAN]	-0,033	0,60	0,009	0,024
Q9Y2W1	Thyroid hormone receptor-associated protein 3 OS=Homo sapiens GN=THRAP3 PE=1 SV=2 - [TR150_HUMAN]	-0,033	0,80	0,010	0,023
Q8IXT5	RNA-binding protein 12B OS=Homo sapiens GN=RBM12B PE=1 SV=2 - [RB12B_HUMAN]	-0,033	0,79	0,017	0,016
P18031	Tyrosine-protein phosphatase non-receptor type 1 OS=Homo sapiens GN=PTPN1 PE=1 SV=1 - [PTN1_HUMAN]	-0,033	0,65	0,012	0,021
Q9HCC0-2	Isoform 2 of Methylcrotonoyl-CoA carboxylase beta chain, mitochondrial OS=Homo sapiens GN=MCCC2 - [MCCB_HUMAN]	-0,033	0,61	0,022	0,011
Q12802-4	Isoform 3 of A-kinase anchor protein 13 OS=Homo sapiens GN=AKAP13 - [AKP13_HUMAN]	-0,033	0,71	0,010	0,023
Q9HBI0-3	Isoform 3 of Gamma-parvin OS=Homo sapiens GN=PARVG - [PARVG_HUMAN]	-0,033	0,33	0,009	0,024
P19484-2	Isoform 2 of Transcription factor EB OS=Homo sapiens GN=TFEB - [TFEB_HUMAN]	-0,033	0,80	0,022	0,011
O00499-9	Isoform BIN1-10-13 of Myc box-dependent-interacting protein 1 OS=Homo sapiens GN=BIN1 - [BIN1_HUMAN]	-0,033	0,71	0,008	0,025
Q9UEE9-2	Isoform 2 of Craniofacial development protein 1 OS=Homo sapiens GN=CFDP1 - [CFDP1_HUMAN]	-0,033	0,80	0,020	0,013
Q9H2W1	Membrane-spanning 4-domains subfamily A member 6A OS=Homo sapiens GN=MS4A6A PE=2 SV=1 - [M4A6A_HUMAN]	-0,032	0,56	0,015	0,017
O14776-2	Isoform 2 of Transcription elongation regulator 1 OS=Homo sapiens GN=TCERG1 - [TCRG1_HUMAN]	-0,032	0,73	0,010	0,022
P49961-3	Isoform 3 of Ectonucleoside triphosphate diphosphohydrolase 1 OS=Homo sapiens GN=ENTPD1 - [ENTP1_HUMAN]	-0,032	0,56	0,004	0,028
P07910-2	Isoform C1 of Heterogeneous nuclear ribonucleoproteins C1/C2 OS=Homo sapiens GN=HNRNPC - [HNRPC_HUMAN]	-0,032	0,76	0,016	0,016
Q92888-2	Isoform 2 of Rho guanine nucleotide exchange factor 1 OS=Homo sapiens GN=ARHGEF1 - [ARHG1_HUMAN]	-0,032	0,80	0,011	0,021
P20339-2	Isoform 2 of Ras-related protein Rab-5A OS=Homo sapiens GN=RAB5A - [RAB5A_HUMAN]	-0,032	0,58	0,022	0,010
Q9BV38	WD repeat-containing protein 18 OS=Homo sapiens GN=WDR18 PE=1 SV=2 - [WDR18_HUMAN]	-0,032	0,74	0,015	0,017
Q86WA6-2	Isoform 2 of Valacyclovir hydrolase OS=Homo sapiens GN=BPHL - [BPHL_HUMAN]	-0,032	0,77	0,017	0,015
Q6UW02	Cytochrome P450 20A1 OS=Homo sapiens GN=CYP20A1 PE=1 SV=1 - [CP20A_HUMAN]	-0,032	0,77	0,026	0,005
Q6DHV7-2	Isoform 2 of Adenosine deaminase-like protein OS=Homo sapiens GN=ADAL - [ADAL_HUMAN]	-0,032	0,78	0,022	0,010

P09874	Poly [ADP-ribose] polymerase 1 OS=Homo sapiens GN=PARP1 PE=1 SV=4 - [PARP1_HUMAN]	-0,032	0,80	0,017	0,015
Q16666-3	Isoform 3 of Gamma-interferon-inducible protein 16 OS=Homo sapiens GN=IFI16 - [IF16_HUMAN]	-0,032	0,52	0,008	0,023
Q8IVF2-3	Isoform 3 of Protein AHNAK2 OS=Homo sapiens GN=AHNAK2 - [AHNK2_HUMAN]	-0,032	0,71	0,020	0,011
Q92692-2	Isoform Alpha of Nectin-2 OS=Homo sapiens GN=PVRL2 - [PVRL2_HUMAN]	-0,032	0,64	0,015	0,017
Q00839-2	Isoform Short of Heterogeneous nuclear ribonucleoprotein U OS=Homo sapiens GN=HNRNPU - [HNRPU_HUMAN]	-0,032	0,93	0,020	0,011
Q9H3G5	Probable serine carboxypeptidase CPVL OS=Homo sapiens GN=CPVL PE=1 SV=2 - [CPVL_HUMAN]	-0,032	0,24	0,011	0,021
Q14141-2	Isoform I of Septin-6 OS=Homo sapiens GN=SEPT6 - [SEPT6_HUMAN]	-0,032	0,61	0,014	0,018
Q9H845	Acyl-CoA dehydrogenase family member 9, mitochondrial OS=Homo sapiens GN=ACAD9 PE=1 SV=1 - [ACAD9_HUMAN]	-0,031	0,48	0,021	0,011
P05787	Keratin, type II cytoskeletal 8 OS=Homo sapiens GN=KRT8 PE=1 SV=7 - [K2C8_HUMAN]	-0,031	0,43	0,029	0,003
Q92688-2	Isoform 2 of Acidic leucine-rich nuclear phosphoprotein 32 family member B OS=Homo sapiens GN=ANP32B - [AN32B_HUMAN]	-0,031	0,57	0,010	0,022
Q8TDZ2-2	Isoform 2 of Protein-methionine sulfoxide oxidase MICAL1 OS=Homo sapiens GN=MICAL1 - [MICA1_HUMAN]	-0,031	0,68	0,025	0,006
P49754-2	Isoform 2 of Vacuolar protein sorting-associated protein 41 homolog OS=Homo sapiens GN=VPS41 - [VPS41_HUMAN]	-0,031	0,69	0,017	0,014
Q9BZH6	WD repeat-containing protein 11 OS=Homo sapiens GN=WDR11 PE=1 SV=1 - [WDR11_HUMAN]	-0,031	0,89	0,019	0,012
Q9NUQ9	Protein FAM49B OS=Homo sapiens GN=FAM49B PE=1 SV=1 - [FA49B_HUMAN]	-0,031	0,49	0,012	0,019
Q96SB3	Neurabin-2 OS=Homo sapiens GN=PPP1R9B PE=1 SV=2 - [NEB2_HUMAN]	-0,031	0,83	0,014	0,017
Q13576	Ras GTPase-activating-like protein IQGAP2 OS=Homo sapiens GN=IQGAP2 PE=1 SV=4 - [IQGA2_HUMAN]	-0,031	0,37	0,010	0,021
Q9H0D6-2	Isoform 2 of 5'-3' exoribonuclease 2 OS=Homo sapiens GN=XRN2 - [XRN2_HUMAN]	-0,031	0,41	0,015	0,016
Q96CX2	BTB/POZ domain-containing protein KCTD12 OS=Homo sapiens GN=KCTD12 PE=1 SV=1 - [KCD12_HUMAN]	-0,031	0,71	0,015	0,016
Q07812-5	Isoform Epsilon of Apoptosis regulator BAX OS=Homo sapiens GN=BAX - [BAX_HUMAN]	-0,031	0,72	0,010	0,020
Q9NVZ3-3	Isoform 3 of Adaptin ear-binding coat-associated protein 2 OS=Homo sapiens GN=NECAP2 - [NECP2_HUMAN]	-0,031	0,53	0,016	0,015
P00488	Coagulation factor XIII A chain OS=Homo sapiens GN=F13A1 PE=1 SV=4 - [F13A_HUMAN]	-0,031	0,39	0,010	0,021
Q92541	RNA polymerase-associated protein RTF1 homolog OS=Homo sapiens GN=RTF1 PE=1 SV=4 - [RTF1_HUMAN]	-0,031	0,84	0,025	0,006
Q9H0Q0	Protein FAM49A OS=Homo sapiens GN=FAM49A PE=2 SV=1 - [FA49A_HUMAN]	-0,031	0,67	0,020	0,011
P98172	Ephrin-B1 OS=Homo sapiens GN=EFNB1 PE=1 SV=1 - [EFNB1_HUMAN]	-0,031	0,70	0,029	0,002
P17813-2	Isoform Short of Endoglin OS=Homo sapiens GN=ENG - [EGLN_HUMAN]	-0,031	0,46	0,022	0,008
Q5VTR2	E3 ubiquitin-protein ligase BRE1A OS=Homo sapiens GN=RNF20 PE=1 SV=2 - [BRE1A_HUMAN]	-0,031	0,86	0,014	0,017
Q16611-2	Isoform 2 of Bcl-2 homologous antagonist/killer OS=Homo sapiens GN=BAK1 - [BAK_HUMAN]	-0,031	0,81	0,012	0,018
P13674-3	Isoform 3 of Prolyl 4-hydroxylase subunit alpha-1 OS=Homo sapiens GN=P4HA1 - [P4HA1_HUMAN]	-0,031	0,62	0,020	0,011
P08575-2	Isoform 2 of Receptor-type tyrosine-protein phosphatase C OS=Homo sapiens GN=PTPRC - [PTPRC_HUMAN]	-0,031	0,38	0,009	0,022
Q9Y4W6	AFG3-like protein 2 OS=Homo sapiens GN=AFG3L2 PE=1 SV=2 - [AFG32_HUMAN]	-0,031	0,81	0,016	0,014
P19338	Nucleolin OS=Homo sapiens GN=NCL PE=1 SV=3 - [NUCL_HUMAN]	-0,031	0,87	0,018	0,012

Q75N90	Fibrillin-3 OS=Homo sapiens GN=FBN3 PE=2 SV=3 - [FBN3_HUMAN]	-0,031	0,80	0,017	0,013
Q8N1F7	Nuclear pore complex protein Nup93 OS=Homo sapiens GN=NUP93 PE=1 SV=2 - [NUP93_HUMAN]	-0,030	0,76	0,016	0,014
P13796	Plastin-2 OS=Homo sapiens GN=LCP1 PE=1 SV=6 - [PLSL_HUMAN]	-0,030	0,28	0,009	0,021
Q9NY15	Stabilin-1 OS=Homo sapiens GN=STAB1 PE=1 SV=3 - [STAB1_HUMAN]	-0,030	0,44	0,019	0,012
Q07955-3	Isoform ASF-3 of Serine/arginine-rich splicing factor 1 OS=Homo sapiens GN=SRSF1 - [SRSF1_HUMAN]	-0,030	0,86	0,019	0,012
Q969G3-6	Isoform 6 of SWI/SNF-related matrix-associated actin-dependent regulator of chromatin subfamily E member 1 OS=Homo sapiens GN=SMARCE1 - [SMCE1_HUMAN]	-0,030	0,89	0,021	0,009
Q9Y394-2	Isoform 2 of Dehydrogenase/reductase SDR family member 7 OS=Homo sapiens GN=DHRS7 - [DHRS7_HUMAN]	-0,030	0,79	0,021	0,010
O60762	Dolichol-phosphate mannosyltransferase subunit 1 OS=Homo sapiens GN=DPM1 PE=1 SV=1 - [DPM1_HUMAN]	-0,030	0,74	0,027	0,003
Q9Y6W5	Wiskott-Aldrich syndrome protein family member 2 OS=Homo sapiens GN=WASF2 PE=1 SV=3 - [WASF2_HUMAN]	-0,030	0,79	0,018	0,012
P46459-2	Isoform 2 of Vesicle-fusing ATPase OS=Homo sapiens GN=NSF - [NSF_HUMAN]	-0,030	0,81	0,027	0,003
P33897	ATP-binding cassette sub-family D member 1 OS=Homo sapiens GN=ABCD1 PE=1 SV=2 - [ABCD1_HUMAN]	-0,030	0,70	0,021	0,010
Q9BSB4	Autophagy-related protein 101 OS=Homo sapiens GN=ATG101 PE=1 SV=1 - [ATGA1_HUMAN]	-0,030	0,56	0,021	0,009
Q14764	Major vault protein OS=Homo sapiens GN=MVP PE=1 SV=4 - [MVP_HUMAN]	-0,030	0,65	0,012	0,018
Q9BW27-3	Isoform 3 of Nuclear pore complex protein Nup85 OS=Homo sapiens GN=NUP85 - [NUP85_HUMAN]	-0,030	0,76	0,020	0,010
P48681	Nestin OS=Homo sapiens GN=NES PE=1 SV=2 - [NEST_HUMAN]	-0,030	0,72	0,015	0,015
Q15029-2	Isoform 2 of 116 kDa U5 small nuclear ribonucleoprotein component OS=Homo sapiens GN=EFTUD2 - [U5S1_HUMAN]	-0,030	0,60	0,015	0,015
P78527	DNA-dependent protein kinase catalytic subunit OS=Homo sapiens GN=PRKDC PE=1 SV=3 - [PRKDC_HUMAN]	-0,030	0,86	0,020	0,010
Q96B97-2	Isoform 2 of SH3 domain-containing kinase-binding protein 1 OS=Homo sapiens GN=SH3KBP1 - [SH3K1_HUMAN]	-0,030	0,61	0,011	0,019
Q92608	Dedicator of cytokinesis protein 2 OS=Homo sapiens GN=DOCK2 PE=1 SV=2 - [DOCK2_HUMAN]	-0,030	0,40	0,011	0,019
Q6NUQ1	RAD50-interacting protein 1 OS=Homo sapiens GN=RINT1 PE=1 SV=1 - [RINT1_HUMAN]	-0,029	0,75	0,024	0,005
P41218	Myeloid cell nuclear differentiation antigen OS=Homo sapiens GN=MNDA PE=1 SV=1 - [MNDA_HUMAN]	-0,029	0,47	0,009	0,021
Q10570	Cleavage and polyadenylation specificity factor subunit 1 OS=Homo sapiens GN=CPSF1 PE=1 SV=2 - [CPSF1_HUMAN]	-0,029	0,74	0,021	0,009
Q9BVK6	Transmembrane emp24 domain-containing protein 9 OS=Homo sapiens GN=TMED9 PE=1 SV=2 - [TMED9_HUMAN]	-0,029	0,66	0,023	0,007
Q99613-2	Isoform 2 of Eukaryotic translation initiation factor 3 subunit C OS=Homo sapiens GN=EIF3C - [EIF3C_HUMAN]	-0,029	0,83	0,019	0,010
P31146	Coronin-1A OS=Homo sapiens GN=CORO1A PE=1 SV=4 - [COR1A_HUMAN]	-0,029	0,28	0,011	0,018
P05362	Intercellular adhesion molecule 1 OS=Homo sapiens GN=ICAM1 PE=1 SV=2 - [ICAM1_HUMAN]	-0,029	0,25	0,016	0,013
P33241-2	Isoform 2 of Lymphocyte-specific protein 1 OS=Homo sapiens GN=LSP1 - [LSP1_HUMAN]	-0,029	0,27	0,011	0,018
Q9H792	Pseudopodium-enriched atypical kinase 1 OS=Homo sapiens GN=PEAK1 PE=1 SV=4 - [PEAK1_HUMAN]	-0,029	0,80	0,020	0,009
P57740-2	Isoform 2 of Nuclear pore complex protein Nup107 OS=Homo sapiens GN=NUP107 -	-0,029	0,78	0,017	0,012

	[NU107_HUMAN]				
P20700	Lamin-B1 OS=Homo sapiens GN=LMNB1 PE=1 SV=2 - [LMNB1_HUMAN]	-0,029	0,45	0,011	0,018
Q96N66-3	Isoform 3 of Lysophospholipid acyltransferase 7 OS=Homo sapiens GN=MBOAT7 - [MBOA7_HUMAN]	-0,029	0,68	0,022	0,008
Q14112-2	Isoform 2 of Nidogen-2 OS=Homo sapiens GN=NID2 - [NID2_HUMAN]	-0,029	0,69	0,016	0,014
Q15528-2	Isoform Surf5A of Mediator of RNA polymerase II transcription subunit 22 OS=Homo sapiens GN=MED22 - [MED22_HUMAN]	-0,029	0,84	0,020	0,009
Q10713	Mitochondrial-processing peptidase subunit alpha OS=Homo sapiens GN=PMPCA PE=1 SV=2 - [MPPA_HUMAN]	-0,029	0,78	0,019	0,010
P06753-5	Isoform 5 of Tropomyosin alpha-3 chain OS=Homo sapiens GN=TPM3 - [TPM3_HUMAN]	-0,029	0,44	0,010	0,019
P31930	Cytochrome b-c1 complex subunit 1, mitochondrial OS=Homo sapiens GN=UQCRC1 PE=1 SV=3 - [QCR1_HUMAN]	-0,029	0,68	0,017	0,012
Q9BXX0	EMILIN-2 OS=Homo sapiens GN=EMILIN2 PE=1 SV=3 - [EMIL2_HUMAN]	-0,029	0,28	0,010	0,019
P50454	Serpin H1 OS=Homo sapiens GN=SERPINH1 PE=1 SV=2 - [SERPH_HUMAN]	-0,029	0,79	0,027	0,003
P10809	60 kDa heat shock protein, mitochondrial OS=Homo sapiens GN=HSPD1 PE=1 SV=2 - [CH60_HUMAN]	-0,029	0,76	0,010	0,019
O15117-2	Isoform FYB-130 of FYN-binding protein OS=Homo sapiens GN=FYB - [FYB_HUMAN]	-0,029	0,52	0,011	0,018
Q9UH62	Armadillo repeat-containing X-linked protein 3 OS=Homo sapiens GN=ARMCX3 PE=1 SV=1 - [ARMX3_HUMAN]	-0,029	0,71	0,023	0,006
Q9BQA9-2	Isoform 2 of Uncharacterized protein C17orf62 OS=Homo sapiens GN=C17orf62 - [CQ062_HUMAN]	-0,029	0,59	0,019	0,010
O75643	U5 small nuclear ribonucleoprotein 200 kDa helicase OS=Homo sapiens GN=SNRNP200 PE=1 SV=2 - [U520_HUMAN]	-0,029	0,84	0,017	0,012
P09917-3	Isoform 3 of Arachidonate 5-lipoxygenase OS=Homo sapiens GN=ALOX5 - [LOX5_HUMAN]	-0,029	0,24	0,008	0,021
Q01105-3	Isoform 3 of Protein SET OS=Homo sapiens GN=SET - [SET_HUMAN]	-0,029	0,75	0,014	0,015
P05783	Keratin, type I cytoskeletal 18 OS=Homo sapiens GN=KRT18 PE=1 SV=2 - [K1C18_HUMAN]	-0,029	0,19	0,028	0,001
Q8WU90	Zinc finger CCCH domain-containing protein 15 OS=Homo sapiens GN=ZC3H15 PE=1 SV=1 - [ZC3HF_HUMAN]	-0,029	0,80	0,022	0,007
P14735	Insulin-degrading enzyme OS=Homo sapiens GN=IDE PE=1 SV=4 - [IDE_HUMAN]	-0,029	0,76	0,016	0,013
Q9UPN7	Serine/threonine-protein phosphatase 6 regulatory subunit 1 OS=Homo sapiens GN=PPP6R1 PE=1 SV=5 - [PP6R1_HUMAN]	-0,029	0,74	0,016	0,013
P29966	Myristoylated alanine-rich C-kinase substrate OS=Homo sapiens GN=MARCKS PE=1 SV=4 - [MARCS_HUMAN]	-0,029	0,39	0,010	0,018
P52566	Rho GDP-dissociation inhibitor 2 OS=Homo sapiens GN=ARHGDIB PE=1 SV=3 - [GDIR2_HUMAN]	-0,029	0,71	0,009	0,019
O43615	Mitochondrial import inner membrane translocase subunit TIM44 OS=Homo sapiens GN=TIMM44 PE=1 SV=2 - [TIM44_HUMAN]	-0,029	0,83	0,021	0,007
O00299	Chloride intracellular channel protein 1 OS=Homo sapiens GN=CLIC1 PE=1 SV=4 - [CLIC1_HUMAN]	-0,029	0,84	0,018	0,010
Q9BRG1	Vacuolar protein-sorting-associated protein 25 OS=Homo sapiens GN=VPS25 PE=1 SV=1 - [VPS25_HUMAN]	-0,029	0,87	0,018	0,010
Q15813	Tubulin-specific chaperone E OS=Homo sapiens GN=TBCE PE=1 SV=1 - [TBCE_HUMAN]	-0,029	0,78	0,025	0,004
P27144	Adenylate kinase 4, mitochondrial OS=Homo sapiens GN=AK4 PE=1 SV=1 - [KAD4_HUMAN]	-0,028	0,79	0,016	0,013
P42166	Lamina-associated polypeptide 2, isoform alpha OS=Homo sapiens GN=TMPO PE=1 SV=2 - [LAP2A_HUMAN]	-0,028	0,69	0,021	0,007
P62888	60S ribosomal protein L30 OS=Homo sapiens GN=RPL30 PE=1 SV=2 - [RL30_HUMAN]	-0,028	0,73	0,018	0,011
Q99735-2	Isoform 2 of Microsomal glutathione S-transferase 2 OS=Homo sapiens GN=MGST2 -	-0,028	0,77	0,025	0,003

	[MGST2_HUMAN]				
Q9UL46	Proteasome activator complex subunit 2 OS=Homo sapiens GN=PSME2 PE=1 SV=4 - [PSME2_HUMAN]	-0,028	0,80	0,019	0,009
P35869	Aryl hydrocarbon receptor OS=Homo sapiens GN=AHR PE=1 SV=2 - [AHR_HUMAN]	-0,028	0,73	0,024	0,005
P27824	Calnexin OS=Homo sapiens GN=CANX PE=1 SV=2 - [CALX_HUMAN]	-0,028	0,85	0,016	0,012
P55058-3	Isoform 3 of Phospholipid transfer protein OS=Homo sapiens GN=PLTP - [PLTP_HUMAN]	-0,028	0,44	0,015	0,013
Q9Y676	28S ribosomal protein S18b, mitochondrial OS=Homo sapiens GN=MRPS18B PE=1 SV=1 - [RT18B_HUMAN]	-0,028	0,70	0,020	0,009
P82909	28S ribosomal protein S36, mitochondrial OS=Homo sapiens GN=MRPS36 PE=1 SV=2 - [RT36_HUMAN]	-0,028	0,80	0,025	0,003
Q9H4B7	Tubulin beta-1 chain OS=Homo sapiens GN=TUBB1 PE=1 SV=1 - [TBB1_HUMAN]	-0,028	0,80	0,025	0,004
P20020-5	Isoform E of Plasma membrane calcium-transporting ATPase 1 OS=Homo sapiens GN=ATP2B1 - [AT2B1_HUMAN]	-0,028	0,72	0,019	0,010
P19971	Thymidine phosphorylase OS=Homo sapiens GN=TYMP PE=1 SV=2 - [TYPH_HUMAN]	-0,028	0,33	0,009	0,019
Q5T447	E3 ubiquitin-protein ligase HECTD3 OS=Homo sapiens GN=HECTD3 PE=1 SV=1 - [HECD3_HUMAN]	-0,028	0,83	0,024	0,005
Q9NZ32	Actin-related protein 10 OS=Homo sapiens GN=ACTR10 PE=1 SV=1 - [ARP10_HUMAN]	-0,028	0,83	0,027	0,001
Q6FI81-3	Isoform 3 of Anamorsin OS=Homo sapiens GN=CIAPIN1 - [CPIN1_HUMAN]	-0,028	0,78	0,012	0,016
P00738	Haptoglobin OS=Homo sapiens GN=HP PE=1 SV=1 - [HPT_HUMAN]	-0,028	0,44	0,023	0,005
Q5T2E6-2	Isoform 2 of UPF0668 protein C10orf76 OS=Homo sapiens GN=C10orf76 - [CJ076_HUMAN]	-0,028	0,76	0,021	0,007
P16220-3	Isoform 3 of Cyclic AMP-responsive element-binding protein 1 OS=Homo sapiens GN=CREB1 - [CREB1_HUMAN]	-0,028	0,80	0,024	0,004
P14406	Cytochrome c oxidase subunit 7A2, mitochondrial OS=Homo sapiens GN=COX7A2 PE=1 SV=1 - [CX7A2_HUMAN]	-0,028	0,61	0,019	0,008
Q9UGP8	Translocation protein SEC63 homolog OS=Homo sapiens GN=SEC63 PE=1 SV=2 - [SEC63_HUMAN]	-0,028	0,79	0,021	0,007
P06753-6	Isoform 6 of Tropomyosin alpha-3 chain OS=Homo sapiens GN=TPM3 - [TPM3_HUMAN]	-0,028	0,42	0,010	0,018
Q14C86-4	Isoform 4 of GTPase-activating protein and VPS9 domain-containing protein 1 OS=Homo sapiens GN=GAPVD1 - [GAPD1_HUMAN]	-0,028	0,92	0,024	0,004
O14745	Na(+)/H(+) exchange regulatory cofactor NHE-RF1 OS=Homo sapiens GN=SLC9A3R1 PE=1 SV=4 - [NHRF1_HUMAN]	-0,028	0,75	0,024	0,004
P08571	Monocyte differentiation antigen CD14 OS=Homo sapiens GN=CD14 PE=1 SV=2 - [CD14_HUMAN]	-0,028	0,39	0,011	0,017
O00592-2	Isoform 2 of Podocalyxin OS=Homo sapiens GN=PODXL - [PODXL_HUMAN]	-0,028	0,53	0,022	0,006
P06865	Beta-hexosaminidase subunit alpha OS=Homo sapiens GN=HEXA PE=1 SV=2 - [HEXA_HUMAN]	-0,028	0,72	0,008	0,020
P04080	Cystatin-B OS=Homo sapiens GN=CSTB PE=1 SV=2 - [CYTB_HUMAN]	-0,028	0,86	0,022	0,006
Q9ULZ3-2	Isoform 2 of Apoptosis-associated speck-like protein containing a CARD OS=Homo sapiens GN=PYCARD - [ASC_HUMAN]	-0,028	0,46	0,014	0,013
P55008	Allograft inflammatory factor 1 OS=Homo sapiens GN=AIF1 PE=1 SV=1 - [AIF1_HUMAN]	-0,028	0,42	0,010	0,017
Q96C19	EF-hand domain-containing protein D2 OS=Homo sapiens GN=EFHD2 PE=1 SV=1 - [EFHD2_HUMAN]	-0,028	0,53	0,012	0,016
Q6P2Q9	Pre-mRNA-processing-splicing factor 8 OS=Homo sapiens GN=PRPF8 PE=1 SV=2 - [PRP8_HUMAN]	-0,028	0,87	0,027	0,001
P19320-2	Isoform 2 of Vascular cell adhesion protein 1 OS=Homo sapiens GN=VCAM1 - [VCAM1_HUMAN]	-0,028	0,53	0,011	0,016
P07585	Decorin OS=Homo sapiens GN=DCN PE=1 SV=1 - [PGS2_HUMAN]	-0,028	0,40	0,015	0,012
Q7L099	Protein RUFY3 OS=Homo sapiens GN=RUFY3 PE=1 SV=1 - [RUFY3_HUMAN]	-0,028	0,85	0,017	0,010

Q9UKK3	Poly [ADP-ribose] polymerase 4 OS=Homo sapiens GN=PARP4 PE=1 SV=3 - [PARP4_HUMAN]	-0,028	0,75	0,015	0,013
P61225	Ras-related protein Rap-2b OS=Homo sapiens GN=RAP2B PE=1 SV=1 - [RAP2B_HUMAN]	-0,027	0,60	0,023	0,004
P51659	Peroxisomal multifunctional enzyme type 2 OS=Homo sapiens GN=HSD17B4 PE=1 SV=3 - [DHB4_HUMAN]	-0,027	0,59	0,019	0,008
P42765	3-ketoacyl-CoA thiolase, mitochondrial OS=Homo sapiens GN=ACAA2 PE=1 SV=2 - [THIM_HUMAN]	-0,027	0,67	0,019	0,008
Q13838	Spliceosome RNA helicase DDX39B OS=Homo sapiens GN=DDX39B PE=1 SV=1 - [DX39B_HUMAN]	-0,027	0,81	0,023	0,005
P04066	Tissue alpha-L-fucosidase OS=Homo sapiens GN=FUCA1 PE=1 SV=4 - [FUCO_HUMAN]	-0,027	0,72	0,017	0,011
Q13637	Ras-related protein Rab-32 OS=Homo sapiens GN=RAB32 PE=1 SV=3 - [RAB32_HUMAN]	-0,027	0,65	0,022	0,005
P36543	V-type proton ATPase subunit E 1 OS=Homo sapiens GN=ATP6V1E1 PE=1 SV=1 - [VATE1_HUMAN]	-0,027	0,69	0,026	0,002
P52948-6	Isoform 6 of Nuclear pore complex protein Nup98-Nup96 OS=Homo sapiens GN=NUP98 - [NUP98_HUMAN]	-0,027	0,84	0,020	0,007
Q8TAQ2-2	Isoform 2 of SWI/SNF complex subunit SMARCC2 OS=Homo sapiens GN=SMARCC2 - [SMRC2_HUMAN]	-0,027	0,91	0,025	0,002
P12270	Nucleoprotein TPR OS=Homo sapiens GN=TPR PE=1 SV=3 - [TPR_HUMAN]	-0,027	0,90	0,017	0,010
Q15833-2	Isoform 2 of Syntaxin-binding protein 2 OS=Homo sapiens GN=STXBP2 - [STXB2_HUMAN]	-0,027	0,21	0,018	0,010
P06737-2	Isoform 2 of Glycogen phosphorylase, liver form OS=Homo sapiens GN=PYGL - [PYGL_HUMAN]	-0,027	0,78	0,022	0,005
P00367	Glutamate dehydrogenase 1, mitochondrial OS=Homo sapiens GN=GLUD1 PE=1 SV=2 - [DHE3_HUMAN]	-0,027	0,82	0,018	0,009
Q99714-2	Isoform 2 of 3-hydroxyacyl-CoA dehydrogenase type-2 OS=Homo sapiens GN=HSD17B10 - [HCD2_HUMAN]	-0,027	0,88	0,022	0,005
Q06033-2	Isoform 2 of Inter-alpha-trypsin inhibitor heavy chain H3 OS=Homo sapiens GN=ITIH3 - [ITIH3_HUMAN]	-0,027	0,40	0,025	0,002
P31040-3	Isoform 3 of Succinate dehydrogenase [ubiquinone] flavoprotein subunit, mitochondrial OS=Homo sapiens GN=SDHA - [SDHA_HUMAN]	-0,027	0,75	0,025	0,002
Q8NEW0	Zinc transporter 7 OS=Homo sapiens GN=SLC30A7 PE=2 SV=1 - [ZNT7_HUMAN]	-0,027	0,48	0,019	0,008
O15269	Serine palmitoyltransferase 1 OS=Homo sapiens GN=SPTLC1 PE=1 SV=1 - [SPTC1_HUMAN]	-0,027	0,79	0,009	0,018
P08238	Heat shock protein HSP 90-beta OS=Homo sapiens GN=HSP90AB1 PE=1 SV=4 - [HS90B_HUMAN]	-0,027	0,87	0,019	0,008
P43490	Nicotinamide phosphoribosyltransferase OS=Homo sapiens GN=NAMPT PE=1 SV=1 - [NAMPT_HUMAN]	-0,027	0,47	0,016	0,011
Q10567-2	Isoform B of AP-1 complex subunit beta-1 OS=Homo sapiens GN=AP1B1 - [AP1B1_HUMAN]	-0,027	0,67	0,017	0,010
P23368	NAD-dependent malic enzyme, mitochondrial OS=Homo sapiens GN=ME2 PE=1 SV=1 - [MAOM_HUMAN]	-0,027	0,51	0,022	0,005
Q9BXP5-5	Isoform 5 of Serrate RNA effector molecule homolog OS=Homo sapiens GN=SRRT - [SRRT_HUMAN]	-0,027	0,84	0,024	0,003
Q16799	Reticulon-1 OS=Homo sapiens GN=RTN1 PE=1 SV=1 - [RTN1_HUMAN]	-0,027	0,71	0,016	0,010
O76070	Gamma-synuclein OS=Homo sapiens GN=SNCG PE=1 SV=2 - [SYUG_HUMAN]	-0,027	0,72	0,016	0,010
P15586-2	Isoform 2 of N-acetylglucosamine-6-sulfatase OS=Homo sapiens GN=GNS - [GNS_HUMAN]	-0,027	0,74	0,011	0,016
Q9Y624-2	Isoform 2 of Junctional adhesion molecule A OS=Homo sapiens GN=F11R - [JAM1_HUMAN]	-0,027	0,50	0,018	0,009
O00116	Alkyldihydroxyacetonephosphate synthase, peroxisomal OS=Homo sapiens GN=AGPS PE=1 SV=1 - [ADAS_HUMAN]	-0,027	0,56	0,016	0,011
P36897	TGF-beta receptor type-1 OS=Homo sapiens GN=TGFBR1 PE=1 SV=1 - [TGFR1_HUMAN]	-0,027	0,84	0,023	0,003
P51606	N-acylglucosamine 2-epimerase OS=Homo sapiens GN=RENBP PE=1 SV=2 - [RENBP_HUMAN]	-0,027	0,48	0,018	0,009

P38117	Electron transfer flavoprotein subunit beta OS=Homo sapiens GN=ETFB PE=1 SV=3 - [ETFB_HUMAN]	-0,027	0,91	0,017	0,010
P07339	Cathepsin D OS=Homo sapiens GN=CTSD PE=1 SV=1 - [CATD_HUMAN]	-0,027	0,62	0,019	0,008
Q96G03	Phosphoglucomutase-2 OS=Homo sapiens GN=PGM2 PE=1 SV=4 - [PGM2_HUMAN]	-0,027	0,85	0,011	0,016
P24557-4	Isoform 4 of Thromboxane-A synthase OS=Homo sapiens GN=TBXAS1 - [THAS_HUMAN]	-0,027	0,31	0,017	0,009
P14314-2	Isoform 2 of Glucosidase 2 subunit beta OS=Homo sapiens GN=PRKCSH - [GLU2B_HUMAN]	-0,026	0,81	0,017	0,009
Q13185	Chromobox protein homolog 3 OS=Homo sapiens GN=CBX3 PE=1 SV=4 - [CBX3_HUMAN]	-0,026	0,79	0,025	0,001
O95571	Persulfide dioxygenase ETHE1, mitochondrial OS=Homo sapiens GN=ETHE1 PE=1 SV=2 - [ETHE1_HUMAN]	-0,026	0,66	0,012	0,015
P07203	Glutathione peroxidase 1 OS=Homo sapiens GN=GPX1 PE=1 SV=4 - [GPX1_HUMAN]	-0,026	0,64	0,012	0,014
Q9Y3Z3-4	Isoform 4 of Deoxynucleoside triphosphate triphosphohydrolase SAMHD1 OS=Homo sapiens GN=SAMHD1 - [SAMH1_HUMAN]	-0,026	0,46	0,018	0,009
P38919	Eukaryotic initiation factor 4A-III OS=Homo sapiens GN=EIF4A3 PE=1 SV=4 - [IF4A3_HUMAN]	-0,026	0,86	0,017	0,009
P15311	Ezrin OS=Homo sapiens GN=EZR PE=1 SV=4 - [EZRI_HUMAN]	-0,026	0,86	0,021	0,005
Q9H3U5-2	Isoform 2 of Major facilitator superfamily domain-containing protein 1 OS=Homo sapiens GN=MFSD1 - [MFSD1_HUMAN]	-0,026	0,64	0,012	0,014
P50225-2	Isoform 2 of Sulfotransferase 1A1 OS=Homo sapiens GN=SULT1A1 - [ST1A1_HUMAN]	-0,026	0,47	0,021	0,006
Q7Z2Y5-2	Isoform 2 of Nik-related protein kinase OS=Homo sapiens GN=NRK - [NRK_HUMAN]	-0,026	0,59	0,011	0,015
P35237	Serpin B6 OS=Homo sapiens GN=SERPINB6 PE=1 SV=3 - [SPB6_HUMAN]	-0,026	0,85	0,021	0,005
P23786	Carnitine O-palmitoyltransferase 2, mitochondrial OS=Homo sapiens GN=CPT2 PE=1 SV=2 - [CPT2_HUMAN]	-0,026	0,64	0,022	0,004
Q5JTZ9	Alanine--tRNA ligase, mitochondrial OS=Homo sapiens GN=AARS2 PE=1 SV=1 - [SYAM_HUMAN]	-0,026	0,68	0,021	0,006
P10253	Lysosomal alpha-glucosidase OS=Homo sapiens GN=GAA PE=1 SV=4 - [LYAG_HUMAN]	-0,026	0,75	0,015	0,011
P09493-5	Isoform 5 of Tropomyosin alpha-1 chain OS=Homo sapiens GN=TPM1 - [TPM1_HUMAN]	-0,026	0,70	0,017	0,009
Q12907	Vesicular integral-membrane protein VIP36 OS=Homo sapiens GN=LMAN2 PE=1 SV=1 - [LMAN2_HUMAN]	-0,026	0,62	0,018	0,009
Q6I9Y2	THO complex subunit 7 homolog OS=Homo sapiens GN=THOC7 PE=1 SV=3 - [THOC7_HUMAN]	-0,026	0,80	0,017	0,010
P27487	Dipeptidyl peptidase 4 OS=Homo sapiens GN=DPP4 PE=1 SV=2 - [DPP4_HUMAN]	-0,026	0,29	0,018	0,008
Q96GD0	Pyridoxal phosphate phosphatase OS=Homo sapiens GN=PDXP PE=1 SV=2 - [PLPP_HUMAN]	-0,026	0,90	0,023	0,004
P22897	Macrophage mannose receptor 1 OS=Homo sapiens GN=MRC1 PE=1 SV=1 - [MRC1_HUMAN]	-0,026	0,64	0,015	0,011
O75937	DnaJ homolog subfamily C member 8 OS=Homo sapiens GN=DNAJC8 PE=1 SV=2 - [DNJC8_HUMAN]	-0,026	0,75	0,025	0,001
Q5T1M5-2	Isoform 2 of FK506-binding protein 15 OS=Homo sapiens GN=FKBP15 - [FKB15_HUMAN]	-0,026	0,71	0,021	0,005
O95466	Formin-like protein 1 OS=Homo sapiens GN=FMNL1 PE=1 SV=3 - [FMNL1_HUMAN]	-0,026	0,40	0,013	0,013
Q16222-2	Isoform AGX1 of UDP-N-acetylhexosamine pyrophosphorylase OS=Homo sapiens GN=UAP1 - [UAP1_HUMAN]	-0,026	0,79	0,018	0,009
Q9UBR2	Cathepsin Z OS=Homo sapiens GN=CTSZ PE=1 SV=1 - [CATZ_HUMAN]	-0,026	0,65	0,023	0,003
P46060	Ran GTPase-activating protein 1 OS=Homo sapiens GN=RANGAP1 PE=1 SV=1 - [RAGP1_HUMAN]	-0,026	0,81	0,016	0,010
P14317	Hematopoietic lineage cell-specific protein OS=Homo sapiens GN=HCLS1 PE=1 SV=3 - [HCLS1_HUMAN]	-0,026	0,51	0,016	0,010
P55884	Eukaryotic translation initiation factor 3 subunit B OS=Homo sapiens GN=EIF3B PE=1 SV=3 - [EIF3B_HUMAN]	-0,026	0,83	0,022	0,004

P09651-3	Isoform 2 of Heterogeneous nuclear ribonucleoprotein A1 OS=Homo sapiens GN=HNRNPA1 - [ROA1_HUMAN]	-0,026	0,81	0,017	0,009
P17844-2	Isoform 2 of Probable ATP-dependent RNA helicase DDX5 OS=Homo sapiens GN=DDX5 - [DDX5_HUMAN]	-0,026	0,78	0,016	0,010
Q9UHX1-4	Isoform 4 of Poly(U)-binding-splicing factor PUF60 OS=Homo sapiens GN=PUF60 - [PUF60_HUMAN]	-0,026	0,73	0,021	0,005
Q9HDC9-2	Isoform 2 of Adipocyte plasma membrane-associated protein OS=Homo sapiens GN=APMAP - [APMAP_HUMAN]	-0,026	0,77	0,020	0,006
Q96T51	RUN and FYVE domain-containing protein 1 OS=Homo sapiens GN=RUFY1 PE=1 SV=2 - [RUFY1_HUMAN]	-0,026	0,78	0,016	0,010
P16284-3	Isoform Delta13 of Platelet endothelial cell adhesion molecule OS=Homo sapiens GN=PECAM1 - [PECA1_HUMAN]	-0,026	0,56	0,022	0,004
O95202	LETM1 and EF-hand domain-containing protein 1, mitochondrial OS=Homo sapiens GN=LETM1 PE=1 SV=1 - [LETM1_HUMAN]	-0,026	0,79	0,019	0,007
P28838-2	Isoform 2 of Cytosol aminopeptidase OS=Homo sapiens GN=LAP3 - [AMPL_HUMAN]	-0,026	0,64	0,013	0,013
P25774-2	Isoform 2 of Cathepsin S OS=Homo sapiens GN=CTSS - [CATS_HUMAN]	-0,026	0,17	0,020	0,006
P49792	E3 SUMO-protein ligase RanBP2 OS=Homo sapiens GN=RANBP2 PE=1 SV=2 - [RBP2_HUMAN]	-0,026	0,80	0,024	0,002
Q8IZ83-3	Isoform 3 of Aldehyde dehydrogenase family 16 member A1 OS=Homo sapiens GN=ALDH16A1 - [A16A1_HUMAN]	-0,026	0,76	0,025	0,001
P34897-3	Isoform 3 of Serine hydroxymethyltransferase, mitochondrial OS=Homo sapiens GN=SHMT2 - [GLYM_HUMAN]	-0,026	0,61	0,014	0,012
P62995-3	Isoform 3 of Transformer-2 protein homolog beta OS=Homo sapiens GN=TRA2B - [TRA2B_HUMAN]	-0,026	0,81	0,013	0,013
Q32P28-4	Isoform 4 of Prolyl 3-hydroxylase 1 OS=Homo sapiens GN=P3H1 - [P3H1_HUMAN]	-0,026	0,79	0,017	0,009
Q96TA1-2	Isoform 2 of Niban-like protein 1 OS=Homo sapiens GN=FAM129B - [NIBL1_HUMAN]	-0,026	0,75	0,014	0,011
Q86UX7-2	Isoform 2 of Fermitin family homolog 3 OS=Homo sapiens GN=FERMT3 - [URP2_HUMAN]	-0,026	0,32	0,014	0,011
O60664-4	Isoform 4 of Perilipin-3 OS=Homo sapiens GN=PLIN3 - [PLIN3_HUMAN]	-0,026	0,84	0,018	0,008
Q12965	Unconventional myosin-Ie OS=Homo sapiens GN=MYO1E PE=1 SV=2 - [MYO1E_HUMAN]	-0,026	0,83	0,017	0,008
P50897-2	Isoform 2 of Palmitoyl-protein thioesterase 1 OS=Homo sapiens GN=PPT1 - [PPT1_HUMAN]	-0,025	0,79	0,018	0,008
P07686	Beta-hexosaminidase subunit beta OS=Homo sapiens GN=HEXB PE=1 SV=3 - [HEXB_HUMAN]	-0,025	0,65	0,018	0,008
P20839-2	Isoform 2 of Inosine-5'-monophosphate dehydrogenase 1 OS=Homo sapiens GN=IMPDH1 - [IMDH1_HUMAN]	-0,025	0,65	0,013	0,012
P13667	Protein disulfide-isomerase A4 OS=Homo sapiens GN=PDIA4 PE=1 SV=2 - [PDIA4_HUMAN]	-0,025	0,80	0,009	0,016
Q96I99	Succinyl-CoA ligase [GDP-forming] subunit beta, mitochondrial OS=Homo sapiens GN=SUCLG2 PE=1 SV=2 - [SUCB2_HUMAN]	-0,025	0,76	0,022	0,003
P05496	ATP synthase F(0) complex subunit C1, mitochondrial OS=Homo sapiens GN=ATP5G1 PE=1 SV=2 - [AT5G1_HUMAN]	-0,025	0,86	0,020	0,006
O14672	Disintegrin and metalloproteinase domain-containing protein 10 OS=Homo sapiens GN=ADAM10 PE=1 SV=1 - [ADA10_HUMAN]	-0,025	0,47	0,014	0,011
O75348	V-type proton ATPase subunit G 1 OS=Homo sapiens GN=ATP6V1G1 PE=1 SV=3 - [VATG1_HUMAN]	-0,025	0,77	0,014	0,011
P49748-2	Isoform 2 of Very long-chain specific acyl-CoA dehydrogenase, mitochondrial OS=Homo sapiens GN=ACADVL - [ACADV_HUMAN]	-0,025	0,82	0,016	0,009
O43143	Pre-mRNA-splicing factor ATP-dependent RNA helicase DHX15 OS=Homo sapiens GN=DHX15 PE=1 SV=2 - [DHX15_HUMAN]	-0,025	0,95	0,022	0,003
P29350	Tyrosine-protein phosphatase non-receptor type 6 OS=Homo sapiens GN=PTPN6 PE=1 SV=1 -	-0,025	0,28	0,016	0,009

	[PTN6_HUMAN]				
Q13107-2	Isoform 2 of Ubiquitin carboxyl-terminal hydrolase 4 OS=Homo sapiens GN=USP4 - [UBP4_HUMAN]	-0,025	0,83	0,015	0,010
Q8NHP6	Motile sperm domain-containing protein 2 OS=Homo sapiens GN=MOSPD2 PE=1 SV=1 - [MSPD2_HUMAN]	-0,025	0,86	0,024	0,001
O94906-2	Isoform 2 of Pre-mRNA-processing factor 6 OS=Homo sapiens GN=PRPF6 - [PRP6_HUMAN]	-0,025	0,86	0,025	0,001
P23508	Colorectal mutant cancer protein OS=Homo sapiens GN=MCC PE=1 SV=2 - [CRCM_HUMAN]	-0,025	0,83	0,020	0,005
Q8NBQ5	Estradiol 17-beta-dehydrogenase 11 OS=Homo sapiens GN=HSD17B11 PE=1 SV=3 - [DHB11_HUMAN]	-0,025	0,48	0,022	0,003
Q96CW1-2	Isoform 2 of AP-2 complex subunit mu OS=Homo sapiens GN=AP2M1 - [AP2M1_HUMAN]	-0,025	0,86	0,024	0,001
Q96RQ3	Methylcrotonoyl-CoA carboxylase subunit alpha, mitochondrial OS=Homo sapiens GN=MCCC1 PE=1 SV=3 - [MCCA_HUMAN]	-0,025	0,84	0,019	0,006
Q8TD55-2	Isoform 2 of Pleckstrin homology domain-containing family O member 2 OS=Homo sapiens GN=PLEKHO2 - [PKHO2_HUMAN]	-0,025	0,67	0,013	0,012
P54819-6	Isoform 6 of Adenylate kinase 2, mitochondrial OS=Homo sapiens GN=AK2 - [KAD2_HUMAN]	-0,025	0,69	0,018	0,007
P60842	Eukaryotic initiation factor 4A-I OS=Homo sapiens GN=EIF4A1 PE=1 SV=1 - [IF4A1_HUMAN]	-0,025	0,77	0,016	0,009
P02786	Transferrin receptor protein 1 OS=Homo sapiens GN=TFRC PE=1 SV=2 - [TFR1_HUMAN]	-0,025	0,57	0,020	0,005
P11021	78 kDa glucose-regulated protein OS=Homo sapiens GN=HSPA5 PE=1 SV=2 - [GRP78_HUMAN]	-0,025	0,87	0,015	0,010
Q9BS26	Endoplasmic reticulum resident protein 44 OS=Homo sapiens GN=ERP44 PE=1 SV=1 - [ERP44_HUMAN]	-0,025	0,70	0,012	0,013
Q92615	La-related protein 4B OS=Homo sapiens GN=LARP4B PE=1 SV=3 - [LAR4B_HUMAN]	-0,025	0,71	0,023	0,002
Q9Y4E1-3	Isoform 3 of WASH complex subunit FAM21C OS=Homo sapiens GN=FAM21C - [FA21C_HUMAN]	-0,025	0,83	0,023	0,001
P26641	Elongation factor 1-gamma OS=Homo sapiens GN=EEF1G PE=1 SV=3 - [EF1G_HUMAN]	-0,025	0,77	0,024	0,001
P14598-2	Isoform 2 of Neutrophil cytosol factor 1 OS=Homo sapiens GN=NCF1 - [NCF1_HUMAN]	-0,025	0,45	0,012	0,012
Q63HN8	E3 ubiquitin-protein ligase RNF213 OS=Homo sapiens GN=RNF213 PE=1 SV=3 - [RN213_HUMAN]	-0,025	0,73	0,015	0,010
P01903	HLA class II histocompatibility antigen, DR alpha chain OS=Homo sapiens GN=HLA-DRA PE=1 SV=1 - [DRA_HUMAN]	-0,025	0,39	0,011	0,013
P22681	E3 ubiquitin-protein ligase CBL OS=Homo sapiens GN=CBL PE=1 SV=2 - [CBL_HUMAN]	-0,025	0,81	0,018	0,006
O43837	Isocitrate dehydrogenase [NAD] subunit beta, mitochondrial OS=Homo sapiens GN=IDH3B PE=1 SV=2 - [IDH3B_HUMAN]	-0,025	0,83	0,016	0,009
Q9UHD8-7	Isoform 7 of Septin-9 OS=Homo sapiens GN=SEPT9 - [SEPT9_HUMAN]	-0,025	0,84	0,016	0,009
Q6NYC8	Phostensin OS=Homo sapiens GN=PPP1R18 PE=1 SV=1 - [PPR18_HUMAN]	-0,025	0,80	0,014	0,011
Q53GL7	Poly [ADP-ribose] polymerase 10 OS=Homo sapiens GN=PARP10 PE=1 SV=2 - [PAR10_HUMAN]	-0,024	0,79	0,015	0,009
P48735-2	Isoform 2 of Isocitrate dehydrogenase [NADP], mitochondrial OS=Homo sapiens GN=IDH2 - [IDHP_HUMAN]	-0,024	0,66	0,017	0,007
P49354	Protein farnesyltransferase/geranylgeranyltransferase type-1 subunit alpha OS=Homo sapiens GN=FNTA PE=1 SV=1 - [FNTA_HUMAN]	-0,024	0,86	0,020	0,004
Q9BZF1-3	Isoform 3 of Oxysterol-binding protein-related protein 8 OS=Homo sapiens GN=OSBPL8 - [OSBL8_HUMAN]	-0,024	0,70	0,018	0,007
Q8NBJ5	Procollagen galactosyltransferase 1 OS=Homo sapiens GN=COLGALT1 PE=1 SV=1 - [GT251_HUMAN]	-0,024	0,64	0,013	0,011
Q71RC2-7	Isoform 7 of La-related protein 4 OS=Homo sapiens GN=LARP4 - [LARP4_HUMAN]	-0,024	0,78	0,019	0,004
Q27J81-2	Isoform 2 of Inverted formin-2 OS=Homo sapiens GN=INF2 - [INF2_HUMAN]	-0,024	0,86	0,022	0,002
Q96NY7-2	Isoform A of Chloride intracellular channel protein 6 OS=Homo sapiens GN=CLIC6 - [CLIC6_HUMAN]	-0,024	0,56	0,014	0,009

Q9Y2Q3-4	Isoform 4 of Glutathione S-transferase kappa 1 OS=Homo sapiens GN=GSTK1 - [GSTK1_HUMAN]	-0,024	0,52	0,021	0,003
P42331-2	Isoform 2 of Rho GTPase-activating protein 25 OS=Homo sapiens GN=ARHGAP25 - [RHG25_HUMAN]	-0,024	0,40	0,020	0,004
P22307-6	Isoform 6 of Non-specific lipid-transfer protein OS=Homo sapiens GN=SCP2 - [NLTP_HUMAN]	-0,024	0,66	0,022	0,002
Q9NR45	Sialic acid synthase OS=Homo sapiens GN=NANS PE=1 SV=2 - [SIAS_HUMAN]	-0,024	0,69	0,021	0,003
P36551	Oxygen-dependent coproporphyrinogen-III oxidase, mitochondrial OS=Homo sapiens GN=CPOX PE=1 SV=3 - [HEM6_HUMAN]	-0,024	0,70	0,018	0,005
Q96JC1-2	Isoform 2 of Vam6/Vps39-like protein OS=Homo sapiens GN=VPS39 - [VPS39_HUMAN]	-0,024	0,83	0,022	0,001
P61158	Actin-related protein 3 OS=Homo sapiens GN=ACTR3 PE=1 SV=3 - [ARP3_HUMAN]	-0,024	0,93	0,017	0,007
Q9BWM7	Sideroflexin-3 OS=Homo sapiens GN=SFXN3 PE=1 SV=2 - [SFXN3_HUMAN]	-0,024	0,88	0,015	0,009
P53634	Dipeptidyl peptidase 1 OS=Homo sapiens GN=CTSC PE=1 SV=2 - [CATC_HUMAN]	-0,024	0,48	0,016	0,007
P52735-3	Isoform 3 of Guanine nucleotide exchange factor VAV2 OS=Homo sapiens GN=VAV2 - [VAV2_HUMAN]	-0,024	0,80	0,016	0,008
P14625	Endoplasmin OS=Homo sapiens GN=HSP90B1 PE=1 SV=1 - [ENPL_HUMAN]	-0,023	0,89	0,016	0,007
P14780	Matrix metalloproteinase-9 OS=Homo sapiens GN=MMP9 PE=1 SV=3 - [MMP9_HUMAN]	-0,023	0,39	0,017	0,006
P98175-2	Isoform 2 of RNA-binding protein 10 OS=Homo sapiens GN=RBM10 - [RBM10_HUMAN]	-0,023	0,88	0,018	0,005
P61916-2	Isoform 2 of Epididymal secretory protein E1 OS=Homo sapiens GN=NPC2 - [NPC2_HUMAN]	-0,023	0,46	0,018	0,005
P36894	Bone morphogenetic protein receptor type-1A OS=Homo sapiens GN=BMPR1A PE=1 SV=2 - [BMR1A_HUMAN]	-0,023	0,78	0,023	0,000
Q7Z2Z2-2	Isoform 2 of Elongation factor Tu GTP-binding domain-containing protein 1 OS=Homo sapiens GN=EFTUD1 - [ETUD1_HUMAN]	-0,023	0,58	0,023	0,000
Q9NYF8-3	Isoform 3 of Bcl-2-associated transcription factor 1 OS=Homo sapiens GN=BCLAF1 - [BCLF1_HUMAN]	-0,023	0,89	0,022	0,001
P20592	Interferon-induced GTP-binding protein Mx2 OS=Homo sapiens GN=MX2 PE=1 SV=1 - [MX2_HUMAN]	-0,023	0,44	0,023	0,001
P07996	Thrombospondin-1 OS=Homo sapiens GN=THBS1 PE=1 SV=2 - [TSP1_HUMAN]	-0,023	0,42	0,015	0,008
Q9UNK0	Syntaxin-8 OS=Homo sapiens GN=STX8 PE=1 SV=2 - [STX8_HUMAN]	-0,023	0,87	0,016	0,007
Q8N392-2	Isoform 2 of Rho GTPase-activating protein 18 OS=Homo sapiens GN=ARHGAP18 - [RHG18_HUMAN]	-0,023	0,73	0,021	0,002
Q96FS4	Signal-induced proliferation-associated protein 1 OS=Homo sapiens GN=SIPA1 PE=1 SV=1 - [SIPA1_HUMAN]	-0,023	0,64	0,016	0,007
P32969	60S ribosomal protein L9 OS=Homo sapiens GN=RPL9 PE=1 SV=1 - [RL9_HUMAN]	-0,023	0,88	0,020	0,004
O15321-2	Isoform 2 of Transmembrane 9 superfamily member 1 OS=Homo sapiens GN=TM9SF1 - [TM9S1_HUMAN]	-0,023	0,77	0,017	0,006
O60568	Procollagen-lysine,2-oxoglutarate 5-dioxygenase 3 OS=Homo sapiens GN=PLOD3 PE=1 SV=1 - [PLOD3_HUMAN]	-0,023	0,72	0,017	0,005
P22105	Tenascin-X OS=Homo sapiens GN=TNXB PE=1 SV=4 - [TENX_HUMAN]	-0,023	0,71	0,021	0,002
Q96HE7	ERO1-like protein alpha OS=Homo sapiens GN=ERO1A PE=1 SV=2 - [ERO1A_HUMAN]	-0,023	0,73	0,017	0,006
Q96C86	m7GpppX diphosphatase OS=Homo sapiens GN=DCPS PE=1 SV=2 - [DCPS_HUMAN]	-0,023	0,86	0,020	0,002
Q9UNL2	Translocon-associated protein subunit gamma OS=Homo sapiens GN=SSR3 PE=1 SV=1 - [SSRG_HUMAN]	-0,023	0,74	0,016	0,007
O43252	Bifunctional 3'-phosphoadenosine 5'-phosphosulfate synthase 1 OS=Homo sapiens GN=PAPSS1 PE=1 SV=2 - [PAPS1_HUMAN]	-0,023	0,75	0,019	0,004
P40939	Trifunctional enzyme subunit alpha, mitochondrial OS=Homo sapiens GN=HADHA PE=1 SV=2 - [ECHA_HUMAN]	-0,023	0,89	0,020	0,003

Q13765	Nascent polypeptide-associated complex subunit alpha OS=Homo sapiens GN=NACA PE=1 SV=1 - [NACA_HUMAN]	-0,023	0,88	0,017	0,006
O43491	Band 4.1-like protein 2 OS=Homo sapiens GN=EPB41L2 PE=1 SV=1 - [E41L2_HUMAN]	-0,023	0,91	0,022	0,000
O15127	Secretory carrier-associated membrane protein 2 OS=Homo sapiens GN=SCAMP2 PE=1 SV=2 - [SCAM2_HUMAN]	-0,022	0,74	0,014	0,008
Q15084-3	Isoform 3 of Protein disulfide-isomerase A6 OS=Homo sapiens GN=PDIA6 - [PDIA6_HUMAN]	-0,022	0,71	0,018	0,005
O00469-3	Isoform 3 of Procollagen-lysine,2-oxoglutarate 5-dioxygenase 2 OS=Homo sapiens GN=PLOD2 - [PLOD2_HUMAN]	-0,022	0,60	0,014	0,008
P15144	Aminopeptidase N OS=Homo sapiens GN=ANPEP PE=1 SV=4 - [AMPN_HUMAN]	-0,022	0,40	0,019	0,004
Q9NZ08	Endoplasmic reticulum aminopeptidase 1 OS=Homo sapiens GN=ERAP1 PE=1 SV=3 - [ERAP1_HUMAN]	-0,022	0,77	0,019	0,003
Q04837	Single-stranded DNA-binding protein, mitochondrial OS=Homo sapiens GN=SSBP1 PE=1 SV=1 - [SSBP_HUMAN]	-0,022	0,84	0,020	0,003
Q15643	Thyroid receptor-interacting protein 11 OS=Homo sapiens GN=TRIP11 PE=1 SV=3 - [TRIPB_HUMAN]	-0,022	0,88	0,016	0,006
Q92508	Piezo-type mechanosensitive ion channel component 1 OS=Homo sapiens GN=PIEZO1 PE=1 SV=4 - [PIEZ1_HUMAN]	-0,022	0,84	0,009	0,013
Q8N131-2	Isoform 2 of Porimin OS=Homo sapiens GN=TMEM123 - [PORIM_HUMAN]	-0,022	0,75	0,020	0,002
Q96C36	Pyrroline-5-carboxylate reductase 2 OS=Homo sapiens GN=PYCR2 PE=1 SV=1 - [P5CR2_HUMAN]	-0,022	0,76	0,016	0,006
P50851-2	Isoform 2 of Lipopolysaccharide-responsive and beige-like anchor protein OS=Homo sapiens GN=LRBA - [LRBA_HUMAN]	-0,022	0,90	0,016	0,005
Q15149	Plectin OS=Homo sapiens GN=PLEC PE=1 SV=3 - [PLEC_HUMAN]	-0,022	0,84	0,021	0,000
P50453	Serpin B9 OS=Homo sapiens GN=SERPINB9 PE=1 SV=1 - [SPB9_HUMAN]	-0,022	0,73	0,018	0,004
O95340	Bifunctional 3'-phosphoadenosine 5'-phosphosulfate synthase 2 OS=Homo sapiens GN=PAPSS2 PE=1 SV=2 - [PAPS2_HUMAN]	-0,022	0,77	0,016	0,005
P00491	Purine nucleoside phosphorylase OS=Homo sapiens GN=PNP PE=1 SV=2 - [PNPH_HUMAN]	-0,022	0,68	0,017	0,004
Q16363-2	Isoform 2 of Laminin subunit alpha-4 OS=Homo sapiens GN=LAMA4 - [LAMA4_HUMAN]	-0,021	0,59	0,016	0,005
P01877	Ig alpha-2 chain C region OS=Homo sapiens GN=IGHA2 PE=1 SV=3 - [IGHA2_HUMAN]	-0,021	0,69	0,019	0,002
Q9NP77-2	Isoform 2 of RNA polymerase II subunit A C-terminal domain phosphatase SSU72 OS=Homo sapiens GN=SSU72 - [SSU72_HUMAN]	-0,021	0,89	0,019	0,002
Q9HC35-2	Isoform 2 of Echinoderm microtubule-associated protein-like 4 OS=Homo sapiens GN=EML4 - [EMAL4_HUMAN]	-0,021	0,79	0,019	0,002
Q9Y2X3	Nucleolar protein 58 OS=Homo sapiens GN=NOP58 PE=1 SV=1 - [NOP58_HUMAN]	-0,021	0,82	0,020	0,000
P52888	Thimet oligopeptidase OS=Homo sapiens GN=THOP1 PE=1 SV=2 - [THOP1_HUMAN]	-0,021	0,94	0,014	0,006
P14618	Pyruvate kinase PKM OS=Homo sapiens GN=PKM PE=1 SV=4 - [KPYM_HUMAN]	-0,021	0,87	0,016	0,005
Q9NTX5-3	Isoform 3 of Ethylmalonyl-CoA decarboxylase OS=Homo sapiens GN=ECHDC1 - [ECHD1_HUMAN]	-0,021	0,86	0,013	0,007
O60462-4	Isoform B0 of Neuropilin-2 OS=Homo sapiens GN=NRP2 - [NRP2_HUMAN]	-0,020	0,63	0,019	0,001
P36222	Chitinase-3-like protein 1 OS=Homo sapiens GN=CHI3L1 PE=1 SV=2 - [CH3L1_HUMAN]	-0,020	0,45	0,018	0,002
P50452	Serpin B8 OS=Homo sapiens GN=SERPINB8 PE=1 SV=2 - [SPB8_HUMAN]	-0,020	0,78	0,017	0,003
P24821	Tenascin OS=Homo sapiens GN=TNC PE=1 SV=3 - [TENA_HUMAN]	-0,020	0,37	0,013	0,007
Q6PJW8-2	Isoform 2 of Consortin OS=Homo sapiens GN=CNST - [CNST_HUMAN]	-0,020	0,89	0,019	0,001
Q6IA69-2	Isoform 2 of Glutamine-dependent NAD(+) synthetase OS=Homo sapiens GN=NADSYN1 - [NADE_HUMAN]	-0,020	0,77	0,016	0,004

Q32MZ4-3	Isoform 3 of Leucine-rich repeat flightless-interacting protein 1 OS=Homo sapiens GN=LRRFIP1 - [LRRF1_HUMAN]	-0,020	0,83	0,019	0,001
Q9HCH5-15	Isoform 12 of Synaptotagmin-like protein 2 OS=Homo sapiens GN=SYTL2 - [SYTL2_HUMAN]	-0,020	0,75	0,016	0,004
P38606-2	Isoform 2 of V-type proton ATPase catalytic subunit A OS=Homo sapiens GN=ATP6V1A - [VATA_HUMAN]	-0,020	0,84	0,019	0,001
Q5VW36	Focadhesin OS=Homo sapiens GN=FOCAD PE=1 SV=1 - [FOCAD_HUMAN]	-0,020	0,88	0,017	0,003
P55209-3	Isoform 3 of Nucleosome assembly protein 1-like 1 OS=Homo sapiens GN=NAP1L1 - [NP1L1_HUMAN]	-0,020	0,85	0,017	0,003
O14786	Neuropilin-1 OS=Homo sapiens GN=NRP1 PE=1 SV=3 - [NRP1_HUMAN]	-0,020	0,76	0,017	0,003
P35442	Thrombospondin-2 OS=Homo sapiens GN=THBS2 PE=1 SV=2 - [TSP2_HUMAN]	-0,020	0,62	0,018	0,002
Q14571	Inositol 1,4,5-trisphosphate receptor type 2 OS=Homo sapiens GN=ITPR2 PE=1 SV=2 - [ITPR2_HUMAN]	-0,019	0,76	0,019	0,000
P11310	Medium-chain specific acyl-CoA dehydrogenase, mitochondrial OS=Homo sapiens GN=ACADM PE=1 SV=1 - [ACADM_HUMAN]	-0,019	0,77	0,019	0,000
P23381	Tryptophan--tRNA ligase, cytoplasmic OS=Homo sapiens GN=WARS PE=1 SV=2 - [SYWC_HUMAN]	-0,019	0,85	0,017	0,002
P41226	Ubiquitin-like modifier-activating enzyme 7 OS=Homo sapiens GN=UBA7 PE=1 SV=2 - [UBA7_HUMAN]	-0,019	0,85	0,017	0,002
Q9NR99	Matrix-remodeling-associated protein 5 OS=Homo sapiens GN=MXRA5 PE=2 SV=3 - [MXRA5_HUMAN]	-0,019	0,56	0,017	0,002
Q9NZT2-2	Isoform 2 of Opioid growth factor receptor OS=Homo sapiens GN=OGFR - [OGFR_HUMAN]	-0,019	0,81	0,018	0,001
P27797	Calreticulin OS=Homo sapiens GN=CALR PE=1 SV=1 - [CALR_HUMAN]	-0,019	0,86	0,012	0,007
P05164	Myeloperoxidase OS=Homo sapiens GN=MPO PE=1 SV=1 - [PERM_HUMAN]	-0,019	0,52	0,018	0,001
P17301	Integrin alpha-2 OS=Homo sapiens GN=ITGA2 PE=1 SV=1 - [ITA2_HUMAN]	-0,019	0,80	0,017	0,001
P61604	10 kDa heat shock protein, mitochondrial OS=Homo sapiens GN=HSPE1 PE=1 SV=2 - [CH10_HUMAN]	-0,018	0,80	0,016	0,003
P21796	Voltage-dependent anion-selective channel protein 1 OS=Homo sapiens GN=VDAC1 PE=1 SV=2 - [VDAC1_HUMAN]	-0,018	0,78	0,018	0,000
P10619-2	Isoform 2 of Lysosomal protective protein OS=Homo sapiens GN=CTSA - [PPGB_HUMAN]	-0,018	0,70	0,015	0,003
Q70UQ0	Inhibitor of nuclear factor kappa-B kinase-interacting protein OS=Homo sapiens GN=IKBIP PE=1 SV=1 - [IKIP_HUMAN]	-0,018	0,70	0,017	0,001
P07237	Protein disulfide-isomerase OS=Homo sapiens GN=P4HB PE=1 SV=3 - [PDIA1_HUMAN]	-0,017	0,90	0,011	0,006
P40926	Malate dehydrogenase, mitochondrial OS=Homo sapiens GN=MDH2 PE=1 SV=3 - [MDHM_HUMAN]	-0,017	0,84	0,017	0,000
Q13162	Peroxiredoxin-4 OS=Homo sapiens GN=PRDX4 PE=1 SV=1 - [PRDX4_HUMAN]	-0,016	0,82	0,016	0,000
P30040	Endoplasmic reticulum resident protein 29 OS=Homo sapiens GN=ERP29 PE=1 SV=4 - [ERP29_HUMAN]	-0,016	0,87	0,012	0,004

Supplementary Table 5a. KEGG pathway analysis of proteins differentially expressed between BAV-ND and TAV-ND aorta (n=276).

KEGG Gene Set Name	Description	FDR q	Ref
ENDOCYTOSIS	Endocytosis	2.27E-12	14
COMPLEMENT AND COAGULATION CASCADES	Complement and coagulation cascades	6.92E-10	15
VALINE LEUCINE AND ISOLEUCINE DEGRADATION	Valine, leucine and isoleucine degradation	1.16E-5	16
FOCAL ADHESION	Focal adhesion	1.25E-5	17
CITRATE CYCLE TCA CYCLE	Citrate cycle (TCA cycle)	3.76E-5	18
AMINO SUGAR AND NUCLEOTIDE SUGAR METABOLISM	Amino sugar and nucleotide sugar metabolism	1.6E-4	19
ECM RECEPTOR INTERACTION	ECM-receptor interaction	2.36E-4	20
PATHWAYS IN CANCER	Pathways in cancer	4.88E-4	
GLYCOLYSIS GLUCONEOGENESIS	Glycolysis / Gluconeogenesis	5.84E-4	21
ALANINE ASPARTATE ANDGLUTAMATE METABOLISM	Alanine, aspartate and glutamate metabolism	6.00E-04	21,22

Supplementary Table 5b. Hallmark analysis of proteins differentially expressed between BAV-ND and TAV-ND aorta (n=276).

Hallmark Gene Set Name	Description	FDR q	Ref
OXIDATIVE PHOSPHORYLATION	Genes encoding proteins involved in oxidative phosphorylation.	8.16E-9	23
COAGULATION	Genes encoding components of blood coagulation system; also up-regulated in platelets.	1.22E-8	24
EPITHELIAL MESENCHYMAL TRANSITION	Genes defining EMT, as in wound healing, fibrosis and metastasis.	4.27E-6	
COMPLEMENT	Genes encoding components of the complement system, which is part of the innate immune system.	2.45E-5	15
HYPOXIA	Genes up-regulated in response to low oxygen levels (hypoxia).	2.45E-5	25
FATTY ACID METABOLISM	Genes encoding proteins involved in metabolism of fatty acids.	3.08E-5	21
APOPTOSIS	Genes mediating programmed cell death (apoptosis) by activation of caspases.	3.08E-5	26
ADIPOGENESIS	Genes up-regulated during adipocyte differentiation (adipogenesis).	1.05E-4	27
ALLOGRAFT REJECTION	Genes up-regulated during transplant rejection.	1.05E-4	
GLYCOLYSIS	Genes encoding proteins involved in glycolysis and gluconeogenesis.	1.05E-4	21

Supplementary Table 5c. KEGG pathway analysis of proteins differentially expressed between BAV-D and TAV-D aorta (n=805).

KEGG Gene Set Name	Description	FDR q	Ref
REGULATION OF ACTIN CYTOSKELETON	Regulation of actin cytoskeleton	1.14E-22	28
FOCAL ADHESION	Focal adhesion	1.16E-18	17
LEUKOCYTE TRANSENDOTHELIAL MIGRATION	Leukocyte transendothelial migration	6.31E-14	29
ECM RECEPTOR INTERACTION	ECM-receptor interaction	2.74E-12	20
ENDOCYTOSIS	Endocytosis	3.94E-12	14
SPLICEOSOME	Spliceosome	2.27E-10	30
PATHOGENIC ESCHERICHIA COLI INFECTION	Pathogenic Escherichia coli infection	7.43E-9	
PATHWAYS IN CANCER	Pathways in cancer	1.37E-8	
FC GAMMA R MEDIATED PHAGOCYTOSIS	Fc gamma R-mediated phagocytosis	2.28E-8	31
TIGHT JUNCTION	Tight junction	2.36E-8	32

Supplementary Table 5d. Hallmark analysis of proteins differentially expressed between BAV-D and TAV-D aorta (n=805).

Hallmark Gene Set Name	Description	FDR q	Ref
MTORC1 SIGNALING	Genes up-regulated through activation of mTORC1 complex.	3.21E-22	27
EPITHELIAL MESENCHYMAL TRANSITION	Genes defining EMT, as in wound healing, fibrosis and metastasis.	1.98E-21	
MITOTIC SPINDLE	Genes important for mitotic spindle assembly.	1.48E-18	33
MYOGENESIS	Genes involved in development of skeletal muscle (myogenesis).	1.48E-18	34
APICAL JUNCTION	Genes encoding components of apical junction complex.	9.57E-15	32
MYC TARGETS V1	A subgroup of genes regulated by MYC - version 1.	9.57E-15	35
FATTY ACID METABOLISM	Genes encoding proteins involved in metabolism of fatty acids.	2.93E-12	21
PROTEIN SECRETION	Genes involved in protein secretion pathway.	3.71E-12	36
ADIPOGENESIS	Genes up-regulated during adipocyte differentiation (adipogenesis).	3.94E-12	27
OXIDATIVE PHOSPHORYLATION	Genes encoding proteins involved in oxidative phosphorylation.	2.74E-11	23

Supplementary Table 6. Top 40 biological processes from the ingenuity pathway analysis of BAV-ND vs. TAV-ND (n=276) and BAV-D vs. TAV-D (n=805) aortic samples. Ingenuity pathway analysis was performed on all significant predictive loadings from OPLS-DA model according to jack-knife confidence intervals and cross-validation.

Ingenuity Canonical Pathways	-log(p-value)	Ingenuity Canonical Pathways	-log(p-value)
Acute Phase Response Signaling	9.62E00	Actin Cytoskeleton Signaling	1.02E01
Complement System	5,00E00	Integrin Signaling	9.48E00
Acetyl-CoA Biosynthesis I	4.27E00	RhoA Signaling	9.03E00
LXR/RXR Activation	4.27E00	phagosome maturation	8.43E00
Clathrin-mediated Endocytosis Signaling	4.22E00	Glycogen Degradation III	8.39E00
Caveolar-mediated Endocytosis Signaling	4.21E00	Leukocyte Extravasation Signaling	7.97E00
Mechanism of Viral Exit from Host Cells	3.58E00	Signaling by Rho Family GTPases	7.84E00
phagosome maturation	3.58E00	Glycogen Degradation II	7.61E00
Remodel. of Epithelial Adherens Junctions	3.49E00	RhoGDI Signaling	6.6E00
Ceramide Signaling	3.04E00	Epithelial Adherens Junction Signaling	6.23E00
2-ketoglutarate Dehydrogenase Complex	2.94E00	Paxillin Signaling	6.19E00
Extrinsic Prothrombin Activation Pathway	2.87E00	ILK Signaling	6.19E00
Coagulation System	2.85E00	Caveolar-mediated Endocytosis Signaling	5.9E00
Apoptosis Signaling	2.82E00	Aldosterone Signaling in Epithelial Cells	5.26E00
D-myo-inositol (1,4,5,6)-Tetrakisphosphate Biosynth.	2.77E00	Tight Junction Signaling	5.17E00
D-myo-inositol (3,4,5,6)-tetrakisphosphate Biosynth.	2.77E00	Receptor-med. Phagocytosis in Mϕ, Monoc.	5.13E00
PI3K Signaling in B Lymphocytes	2.75E00	Clathrin-mediated Endocytosis Signaling	4.98E00
2-oxobutanoate Degradation I	2.72E00	Ephrin Receptor Signaling	4.93E00
FXR/RXR Activation	2.71E00	Germ Cell-Sertoli Cell Junction Signaling	4.5E00
Tight Junction Signaling	2.61E00	Ephrin B Signaling	4.31E00
Systemic Lupus Erythematosus Signaling	2.58E00	Axonal Guidance Signaling	4.2E00
Arginine Biosynthesis IV	2.55E00	Protein Ubiquitination Pathway	4.12E00
Protein Ubiquitination Pathway	2.49E00	Thrombin Signaling	3.9E00
D-myo-inositol-5-phosphate Metabolism	2.47E00	PI3K/AKT Signaling	3.77E00
3-phosphoinositide Degradation	2.45E00	Protein Kinase A Signaling	3.65E00
Role of Tissue Factor in Cancer	2.39E00	Hepatic Fibrosis/Hepatic Stellate Cell Activ.	3.63E00
Hepatic Fibrosis/Hepatic Stellate Cell Activ.	2.38E00	IL-8 Signaling	3.57E00
Cyclins and Cell Cycle Regulation	2.34E00	Regulation of Actin-based Motility by Rho	3.56E00
Molecular Mechanisms of Cancer	2.3E00	Virus Entry via Endocytic Pathways	3.46E00
3-phosphoinositide Biosynthesis	2.29E00	GŒ±12/13 Signaling	3.39E00
Antiproliferative Role of TOB in T Cell Signaling	2.25E00	Remodeling of Epithelial Adherens Junctions	3.39E00
Leucine Degradation I	2.18E00	Actin Nucleation by ARP-WASP Complex	3.29E00
Intrinsic Prothrombin Activation Pathway	2.16E00	Aryl Hydrocarbon Receptor Signaling	3.25E00
TR/RXR Activation	2.16E00	CXCR4 Signaling	3.19E00
HIPPO signaling	2.16E00	Gap Junction Signaling	3.19E00
PI3K/AKT Signaling	2.14E00	D-myo-inositol (1,4,5)-trisphosphate Degrad	3.15E00
CTLA4 Signaling in Cytotoxic T Lymphocytes	2.12E00	Cellular Effects of Sildenafil (Viagra)	3.14E00
ErbB4 Signaling	2.05E00	Cdc42 Signaling	2.97E00
Chronic Myeloid Leukemia Signaling	2.02E00	PAK Signaling	2.94E00
IL-12 Signaling and Production in Macrophages	1.98E00	14-3-3-mediated Signaling	2.92E00

Supplementary Table 7: PubMed search for 30 top upregulated proteins in BAV-ND

Gene Symbol	Protein Symbol	Function
**CIC**	**CIC (capicua)**	An HMG-box transcriptional repressor that was first identified in Drosophila regulating receptor tyrosine kinase (RTK) signaling. In mammals, CIC mediates RTK-dependent responses has been associated with cell proliferation, neurodegenerative diseases and cancer.[37]
**XIAP**	**XIAP**	E3 ubiquitin-protein ligase, negative regulator of apoptosis and a potential oncogene, autophagy inhibitor,[38] overexpressed in a variety of human cancers[39]
**CDKN1B**	**CDN1B**	Synonym with P27. Regulator of cell cycle the enhanced expression of which is associated with EMT.[40] Differential cellular localization of p27 is associated with cancer.[41]
**DNMT3A**	**DNM3A**	Functions as *de novo* DNA methyltransferase. Activated in cancer.[42] Upregulated in EC exposed to disturbed flow causing increased DNA methylation of CpG islands within the promoter of the flow responsive gene KLF4 *in vivo* and *in vitro*.[43]
**RCN1**	**RCN1**	Functions in secretory pathway.[44] Upegulated in EMT associated with renal fibrosis.[45] Highly upregulated in several type of cancer.[46]
**PSMA6**	**PSA6**	A major component of intracellular protein degradation apparatus. PSMA6 polymorphisms associated with type2 diabetes, myocardial infarction, and coronary artery disease.[47]
**HGS**	**HGS**	Involved in a WNT/β-Catenin dependent cross talk in development of some types of cancer.[48] A component of ESCRT-0 complex, consisting of the hepatocyte growth factor-regulated tyrosine kinase substrate (Hrs) and the signal-transducing adaptor molecule (STAM) proteins, that recognizes ubiquitinylated cargo during the initial step of endosomal sorting.[49]
**PLXDC2**	**PXDC2**	Type I transmembrane protein with some homology to nidogen and plexins, characterized as a mitogen for neural progenitors.[50] The expression of this protein is altered in certain types of cancer.[51]
**POTEI**	**POTEI**	Not much information available in PubMed
**OTUB 1**	**OTUB1**	A deubiquitinating enzyme overexpression of which is associated with several types of cancer via activation of RHOA and EMT.[52,53]
**SNRPB2**	**RU2B**	One of the 11 genes robustly and universally linked to Membrane-Type 1 Matrix Metalloproteinase (MT1-MMP). Key invasion promoting protease in multiple cancerous tumors.[54]
**IARS2**	**SYIM**	Interacting partner of chaperon protein HSP90.[55] Regulation of Dynein function[56] which in turn affects endocytosis[57] and autophagy.[58]
**ABAT**	**GABT**	An enzyme responsible for catabolism of inhibitory neurotransmitter GABA, a new function of this protein in mitochondrial metabolism has been newly discovered.[59]
**TCEAL3**	**TCAL3**	Transcription elongation factor A like 3, not much information available in PubMed
**CNOT11**	**CNO11**	A module of CCR4-NOT complex. This complex is a key regulator of mRNA degradation via destabilization and inhibition of translation.[60]
**ARSD**	**ARSD**	A metallochaperon involved in the response to arsenic environmental pollution.[61]
**MTHFD2**	**MTDC**	Regulator of Vimentin expression during EMT and breast cancer cells migration and invasion.[62] Has a key role in the development of cancers.[63]
**ZYX**	**ZYX**	A component of focal adhesions (FAs) and stress fiber (SF).[64] Mediating the cytoskeletal and extra cellular matrix (ECM) mechanotransduction.[65] Involved in migration of mesenchymal and cancerous cells.[66] Regulates adherens junction integrity and cell motility during the EMT of lung cancer.[67] Involved in migration of the endocardial cells during EndMT of cardiac valve formation.[67]

**IL16**	**IL16**	A regulator of T cell growth and has been associated with several types of cancer.[68]
**C2CD5**	**C2CD5**	Characterized as a component of the MIB1 ubiquitin ligase proteomic interactome.[69]
**SCARB2**	**SCARB2**	A lysosomal membrane glucoprotein involved in autophagy-lysosome pathway.[70]
**C9orf64**	**CI064**	A somatic cell memory gene regulating the difference between induced pluripotent and embryonic stem cells differences and proposed to stabilize the cells in an intermediate stage with characteristics of both somatic and reprogrammed state.[71] Aberrant promoter methylation of this gene is associated with ovarian cancer.[72] Deleted in some cases of acute myeloid leukemia.[73]
**SBSPON**	**BSPO**	A gene differentially expressed in pre-eclamptic placenta, a disease characterized by angiogenic imbalance and disturbed vascular function.[74]
**ME1**	**MAOX**	Regulates the expression of P53.[75] Overexpressed in cancer to cover metabolic needs of cancer cells. Diminished migration and invasion were observed in ME1-repressed cancer cells.[76]
**YAP1**	**YAP1**	Transcriptional regulator of β-catenin/WNT-driven cancers.[77] Required for EndMT in AVC.[78] Upregulates Jag-1 to activate Notch signaling in hepatocarcinoma.[79] Potent promoter of metastasis in breast cancer and melanoma cells.[80]
**SET**	**SET**	A chromatin modifying factor and histone chaperon regulating histone assembly/disassembly.[81] Involved in regulation of histone acetylation and DNA methylation thereby integrating epigenetic states of histones with DNA methylation.[82]
**TBC1D13**	**TBC13**	Interacts with Rab35 which is a key regulator of membrane trafficking and endocytic recycling of numerous protein localized in plasma membrane, clathrin-coated pits and vesicles. Rab35 controls the recycling pathways for regulation of cell adhesion and migration.[83]
**ABHD10**	**ABHDA**	Involved in protein modification. ABHD10 is responsible for deglucuronidation of acyl glucuronides[84] which in turn covalently modifies proteins.[85]
**VPS25**	**VPS25**	Subunit of Endosomal Sorting Complex required for Transport II (ESCR-II) complex involved in the regulation of endosomal biogenesis and functions. Considered to be a tumor suppress through endocytic regulation of NOTCH signaling[86] and by sorting NOTCH in Multi Vesicular Bodies (MVBs).[87] Plays a role in autophagy.[88]
**DLAT**	**ODP2**	Interacts with 20S proteasome component PSMA as a part of ubiquitin-independent protein degradation pathway.[89] Mutations in the gene causes neurodegenerative disease in human.[90]

Supplementary Table 8: PubMed search for 30 top downregulated proteins in BAV-ND

Gene Symbol	Protein Symbol	Function
---	**KV111**	Ig kappa chain V-I region
**PSMC3**	**PRS6A**	An AAA-ATPase component of the 19S regulative subunit of the proteasome and a negative regulator of Akt kinase.[91] It stabilizes and protects the tumor suppressor p14ARF from degradation[92] and inhibits the oncogenic phenotype of transformed cells expressing rbB oncogene. TBP1 overexpression diminished cell proliferation.[93]
---	**KV120**	Ig kappa chain V-I region
**ADAL**	**ADAL**	Functions in enzymatic activation of antineoplastic and antiretroviral agents.[94] Down regulation of this gene was associated with higher incidence of colorectal cancer in African Americans.[95]
**ASAP2**	**ASAP2**	GTPase-activating protein (GAP) involved in regulation of the subcellular localization of paxillin. Overexpression blocked cell migratory activities.[96] Involved in macrophage phagocytosis.[97] The expression of this gene is reduced in renal cell carcinomas compared to non-cancer tissues[98] and proposed to be a biomarker for pituitary tumor.[99]
**ARMCX3**	**ARMX3**	Integral membrane protein of the mitochondrial outer membrane regulating mitochondrial dynamics and trafficking. Interacts with the component of neural crest Sox10.[100] Its degradation is regulated by non-canonical WNT/PKC pathway.[101]
**PPP2R5E**	**2A5E**	A tumor suppressor.[102] Its down regulation suppresses apoptosis and facilitate the growth of gastric cancer cells.[103] and has been reported in several types of cancer.[104-106]
**EPS8L2**	**ES8L2**	A part of a multimolecular complex required for RAC activation leading to actin cytoskeletal remodeling,[107] up regulated in endometrial cancer.[108]
**RPAP3**	**RPAP3**	A pro-apoptosis protein interacting with RNAPII transcription machinery.[109] Loss of this protein improved HeLa cell survival after UV-induced DNA damage.[110] A proposed target for anti-cancer therapy.[111]
**BIRC6**	**BIRC6**	A peripheral membrane protein of the trans-Golgi network functioning as a chimeric E2/E3 ubiquitin ligase as well as being a member of the inhibitor of apoptosis (IAP) family.[112] Interacts with p53 and facilitates its degradation. Its elevated expression has been associated with poor prognosis in several types of cancers.[113-116]
**HP**	**HPT**	A protein that binds free hemoglobin thus preventing heme-iron mediated oxidative stress caused by hemolysis.[117] Administration of this protein has been proposed to be beneficial in the treatment of diabetes[118] and atherosclerosis.[119] Reduced haptoglobin increases the risk of endothelium being exposed to oxidative damage caused by extracellular hemoglobin.[119,120]
---	**HV305**	Ig heavy chain V-III region
**TGFB1**	**TGFB1**	TGFB1, associated with many biological processes involved in aneurysm. In mice, epigenetic silencing of this protein gave rise to a BAV and increased endocardial EndMT
**RAP2A**	**RAP2A**	A member of the small GTPase superfamily. Regulates the stability of LRP6 receptor for Wnt/β-catenin signaling.[121] It is the direct transcription target of P53 and enhances the migration and invasive ability of some cancer cells[122] while the overexpressed of this protein significantly inhibited the migration and invasion of glioma cells via down regulation of AKT.[123] Involved in integrin signaling.[124]
**CFB**	**CFAB**	Complement factor B
**ERAP2**	**ERAP2**	This protein has a key role in the generation of antigenic epitopes and hence may contribute to cancer predisposition through regulation of generation or destruction of tumor antigens.[125] Is associated with Epithelial cell adhesion molecule (EpCAM) which is also a marker for cancer cells.[126]

**KLHDC4**	**KLDC4**	Identified as RNA binding protein in mRNA interactome of proliferating human HeLa cells.[127] Identified as TRF1 interacting protein in a large-scale screening for regulators of telomeres associated proteins in human cells using protein-protein interaction.[128]
**HLA-A**	**1A01**	The MHC class I cell surface receptors involved in the immune response and consists of heterodimeric molecules binding short peptides. Reduced expression of HLA-A has been implicated in tumor progression and metastasis with poorer survival outcome.[129]
**RNPEP**	**AMPB**	A transcription factor involved in the renin-angiotensin-aldosterone system in atheroma.[130]
**IGKC**	**IGKC**	Immunoglobulin κ chain, a robust immune marker for prediction of survival and response to chemotherapy in several types of cancer.[131]
**UBE3C**	**UBE3C**	A ubiquitin protein ligase involved in K29 and k K33 polyubiquitination.[132] Promotes growth and metastasis of renal cell carcinoma via activating Wnt/β-Catenin pathway. Mutation in this gene is associated with hepatocellular carcinoma by regulation of epithelial-mesenchymal transition.[133] Protects cells against harmful accumulation of degraded proteins.[134]
**ABCB9**	**ABCB9**	An ABC transporter transporting cytosolic peptides into the lumen of lysosomes for degradation via ATP hydrolysis.[135]
**BAX**	**BAX**	A cytosolic pro-apoptotic protein causing mitochondrial outer membrane permeabilization by localization to the mitochondrial outer membrane. Down regulated in cancer.[136] Plays a role in cross talk between autophagy and apoptosis.[137]
**---**	**KV104**	Ig kappa chain V-I region
**TBCE**	**TBCE**	A tubulin specific chaperon,[138] folding α-tubulin to promote α /β dimerization, protecting it from misfolded protein stress.[139]
**---**	**KV102**	Ig kappa chain V-I region
**UBR1**	**UBR1**	Ubiquitin ligase associated with protein degradation.[140,141]
**---**	**KV113**	Ig kappa chain V-I region
**RAB12**	**RAB12**	A small GTPase activated in autophagy involved in protein degradation and trafficking.[142,143]
**ZNF207**	**ZNF207**	Microtubule binding zinc finger protein. Associates with spindle microtubules and regulates chromosome alignment.[144,145]

Supplementary Table 9. Gene expression profile comparison between BAV-ND (n=31) vs. TAV-ND (n=22) and BAV-D (n=46) vs. TAV-D (n=24). Gene expression is corrected for age, adapted from Folkersen et al, 2011.[11] ( ) indicates tendency for up- or downregulation with *P* values ranging from >0.05 to <0.1. Loading values for proteins analyzed by HiRiEF and LC-MS/MS are given. A negative loading indicates a lower protein expression in BAV, a positive loading indicates a higher expression in BAV. NS, detected by proteomic analysis but not differentially expressed; -, not detected by proteomic analysis.

	BAV-ND vs. TAV-ND			BAV-D vs. TAV-D		
	mRNA		Protein	mRNA		Protein
	P-value	Down in	Loading	P-value	Down in	Loading
**VEGF Family**						
**VEGFA**	0.08	(BAV)	-	<0.001	BAV	-
**VEGFB**	NS		-	NS		-
**VEGFC**	<0.01	BAV	NS	<0.001	BAV	NS
**VEGFR1 (FLT1)**	<0.05	BAV	-	<0.001	BAV	-
**VEGFR2 (KDR)**	0.09	(BAV)	-	0.065	(BAV)	-
**VEGFR3 (FLT4)**	NS		-	NS		-
**NRP1**	NS		NS	<0.01	BAV	-0.020
**NRP2**	NS		NS	<0.01	BAV	-0.020
**Regulation of Cell Junction**						
**CLDN5**	NS		-	<0.05	BAV	-
**CDH1 (E-Cadherin)**	NS		-	NS		-
**CDH2 (N-Cadherin)**	NS		NS	<0.05	TAV	0.024
**CDH5 (VE-Cadherin)**	NS		-	<0.01	BAV	-
**PECAM1**	NS		NS	<0.001	BAV	-0.026
**PTPRB**	NS		-	<0.05	BAV	-
**ZEB1**	NS		-	0.001	TAV	-
**RHO pathway kinases**						
**MYLK**	NS		-	<0.001	TAV	-
**ROCK2**	NS		NS	<0.05	TAV	0.027
**Regulation of NOTCH**						
**NOTCH1**	0.09	(BAV)	-	<0.01	BAV	-
**NOTCH2**	NS		-	NS		-
**NOTCH3**	NS		NS	0.055	(BAV)	0.025
**NOTCH4**	NS		-	NS		-
**JAG1**	NS		-	NS		-
**JAG2**	NS		-	NS		-
**DLL4**	NS		-	<0.05	BAV	-
**MFNG**	NS		-	<0.001	BAV	-
**MIB1**	<0.05	TAV	NS	<0.05	TAV	0.026

Figure S1

PCA (a and c) and OPLS-DA (b and d) of protein expression data. The analyses were performed on 10 non-dilated (BAV-ND=5, TAV-ND=5) (a-b) and 11 dilated (BAV-D=6, TAV-D=5) (c-d) patients, respectively, and 2894 proteins. Two-dimensional score plot of PCA showing the PC1-PC2 plane, and OPLS-DA showing the first predictive component (tp1) and orthogonal component (to1) plane of BAV (green) and TAV (blue) thoracic aortic tissue samples.

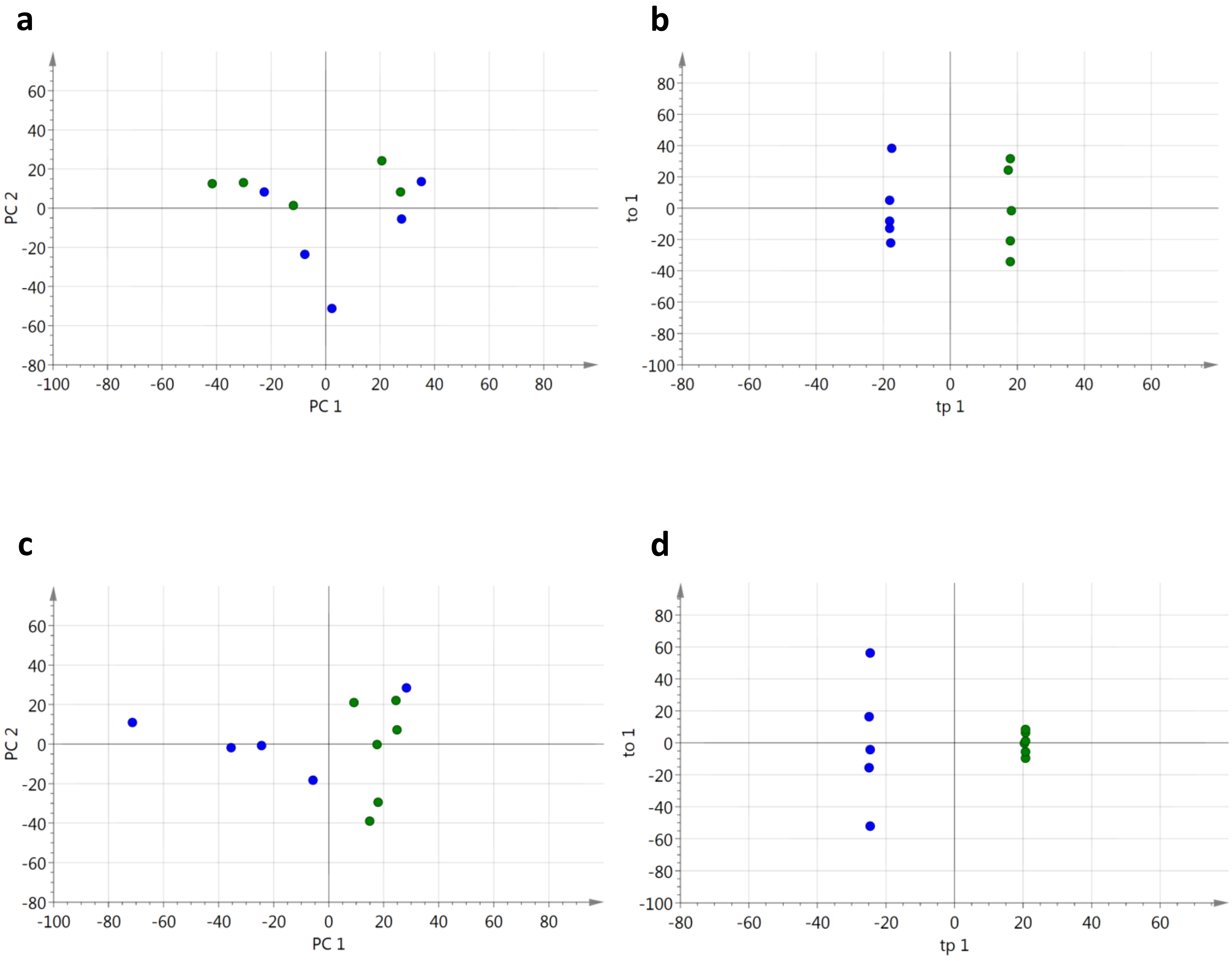

Figure S2

Protein–protein interactions (PPIs) network of proteins involved in enriched Hallmark gene sets shown in Table S5b and S5d. Interactions are retrieved from Human Protein Reference Database (HPRD). Proteins are colored according to the direction of expression change in non-dilated samples and dilated samples respectively. Red indicates up-regulated in BAV compared to TAV; Green indicates down-regulated in BAV and grey indicates unchanged.

a. Non-dilated aorta

b. Dilated aorta

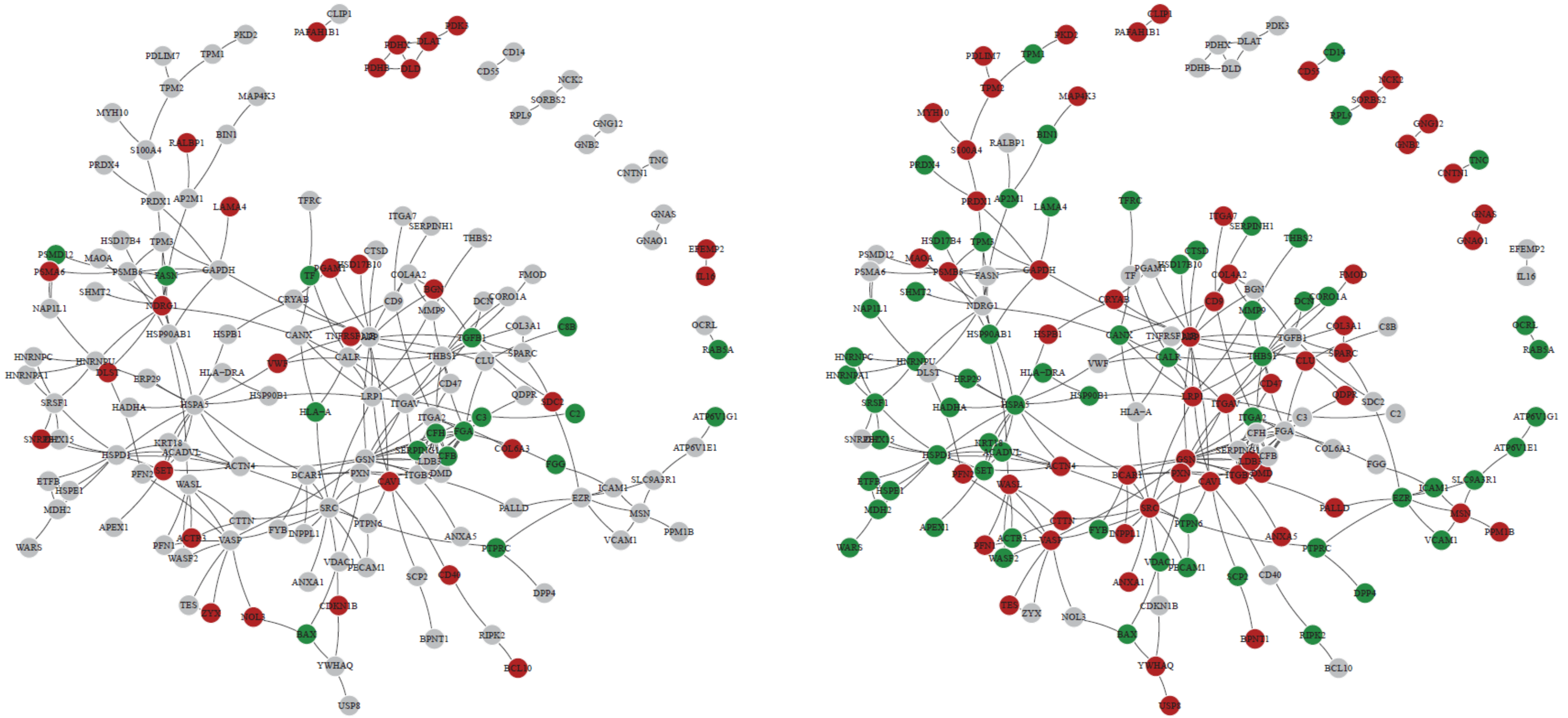

Figure S3 (a-l)

***Higher magnification of major pathways from KEGG and hallmark analyses.* The left and right panels are showing non-dilated and dilated aortas, respectively.** Proteins are colored according to the direction of expression. Red: up-regulated in BAV; Green down-regulated in BAV; Grey unchanged.

Figure S3a. ENDOCYTOSIS

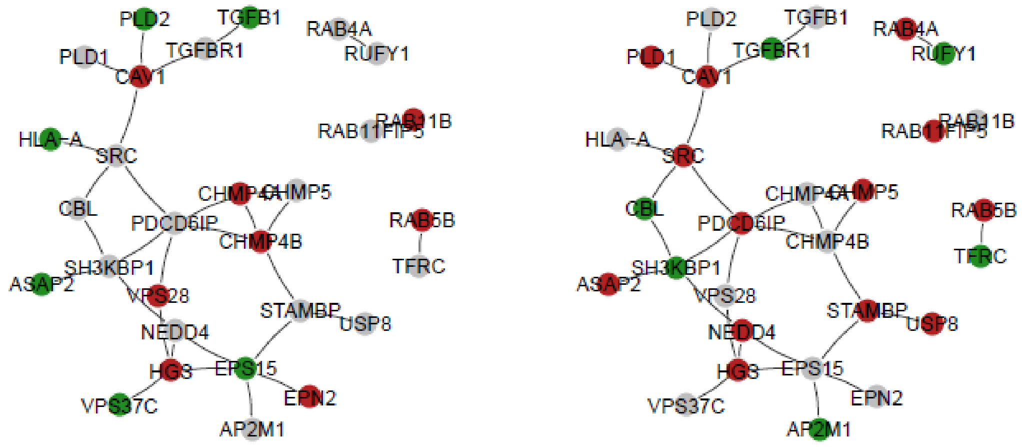

Figure S3b. FOCAL ADHESION

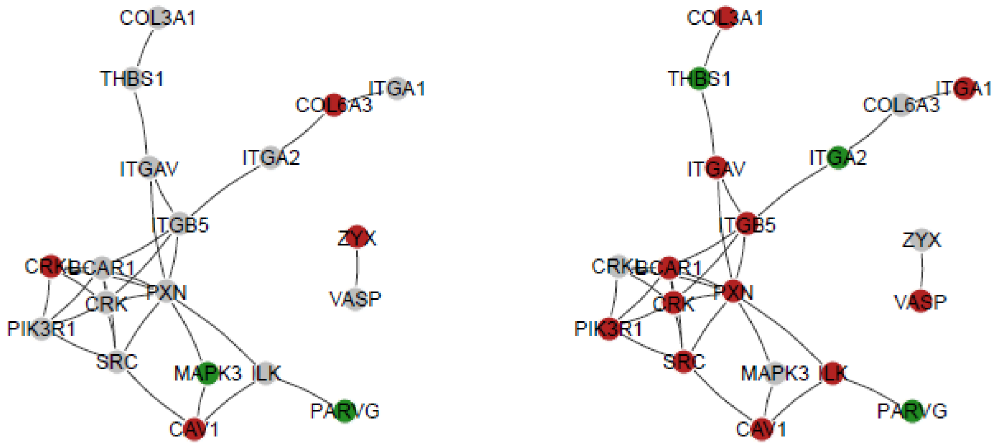

Figure S3c. ECM-RECEPTOR INTERACTION

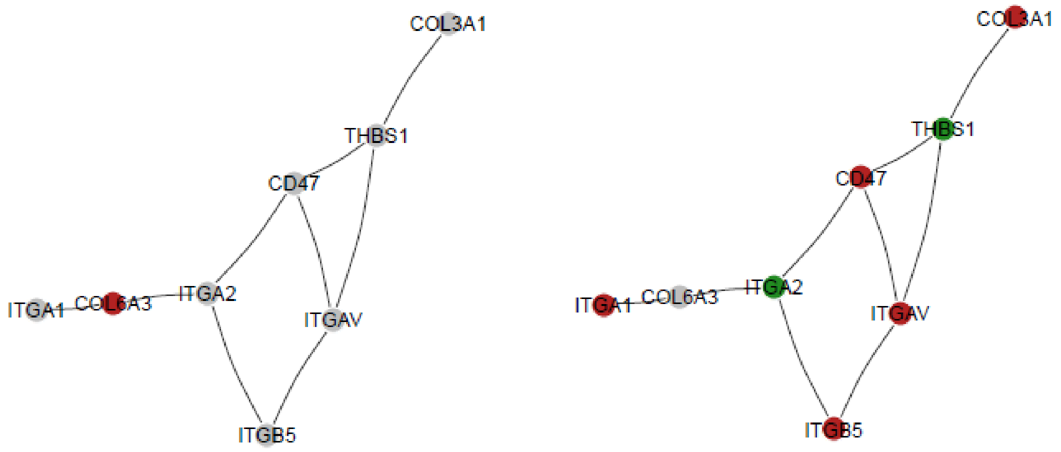

Figure S3d. PATHWAY IN CANCER

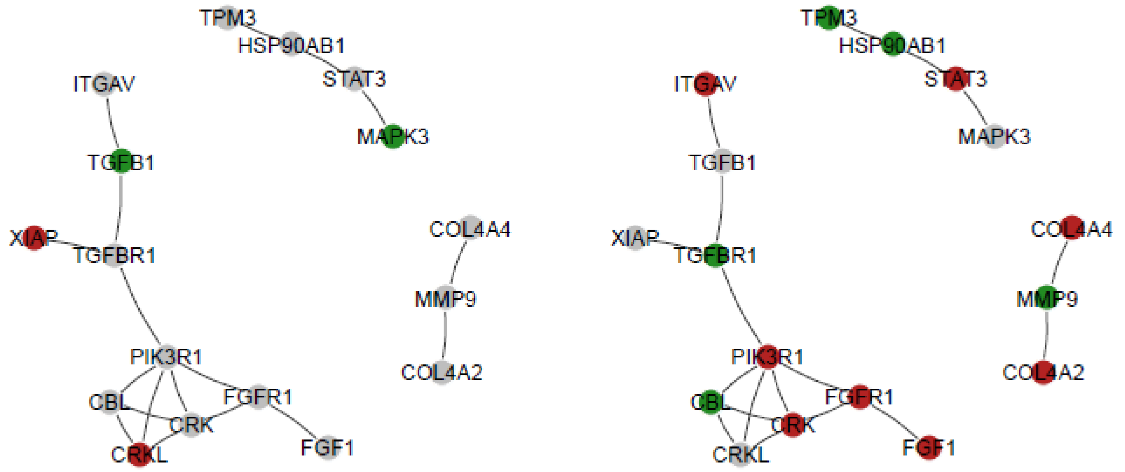

Figure S3e. REGULATION OF ACTIN CYTOSKELETON

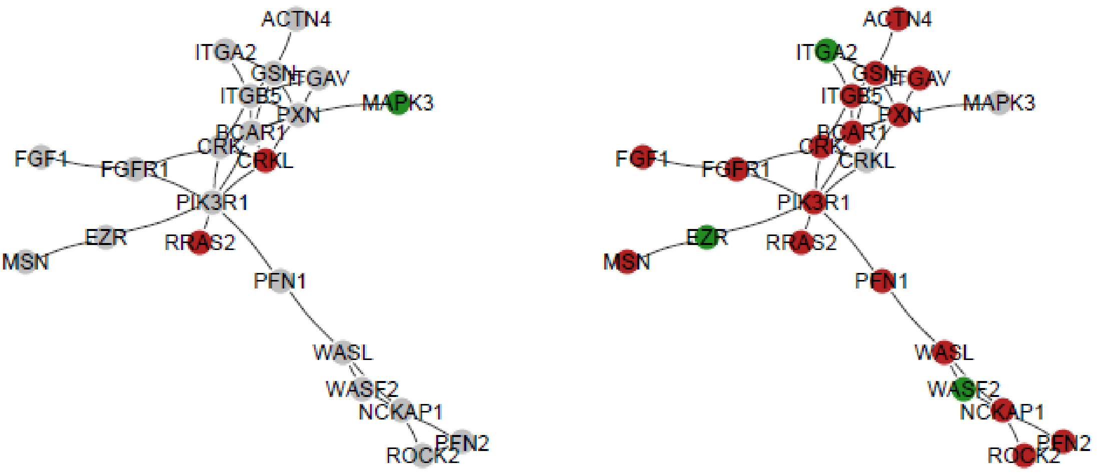

Figure S3f. LEUKOCYTE TRANSENDOTHELIAL MIGRATION

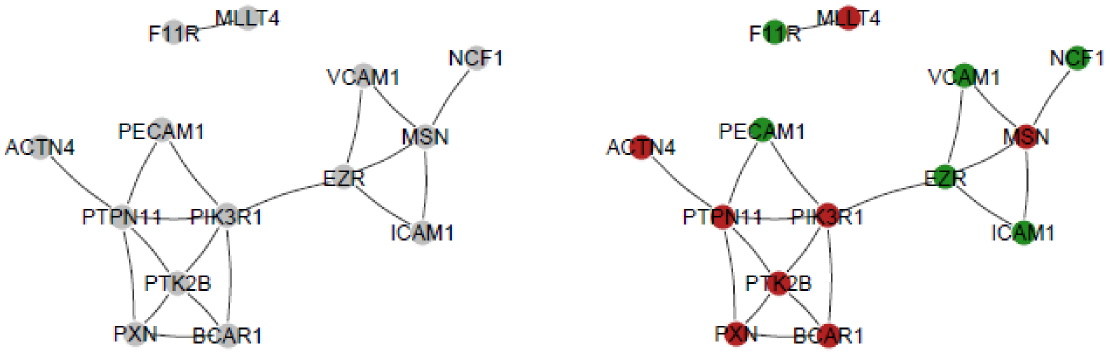

Figure S3g. SPLICEOSOME

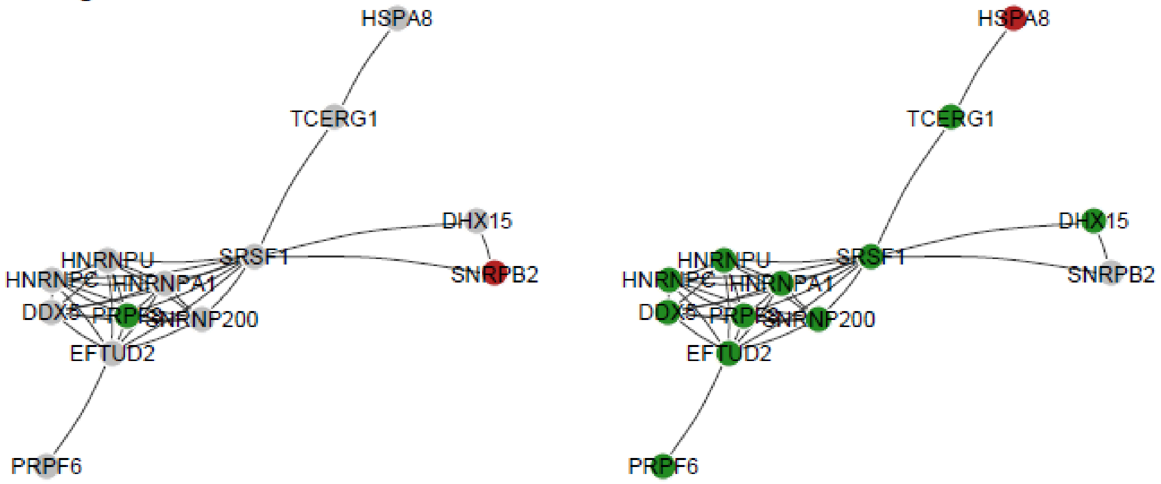

Figure S3h. COAGULATION

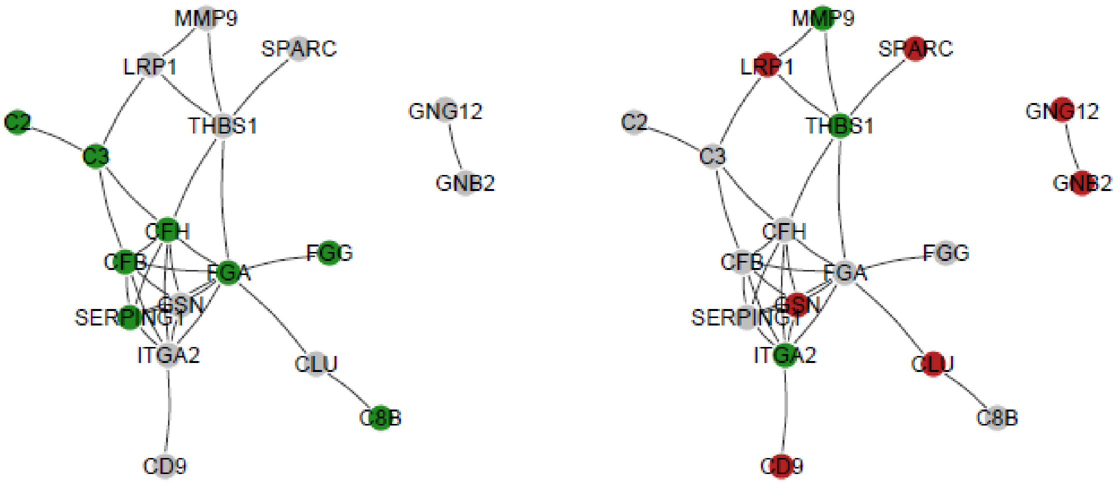

Figure S3i. EPITHELIAL MESENCHYMAL TRANSITION

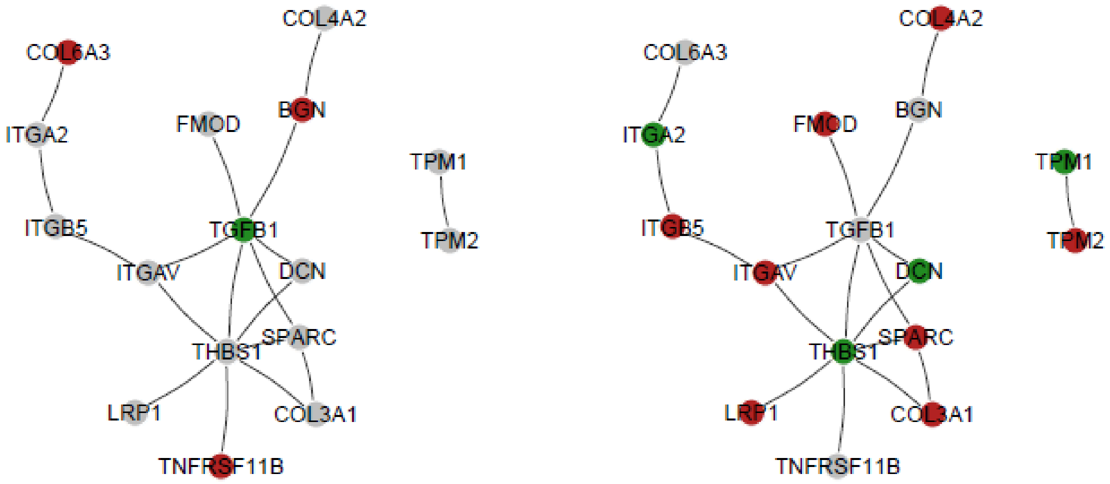

Figure S3j. MYOGENESIS

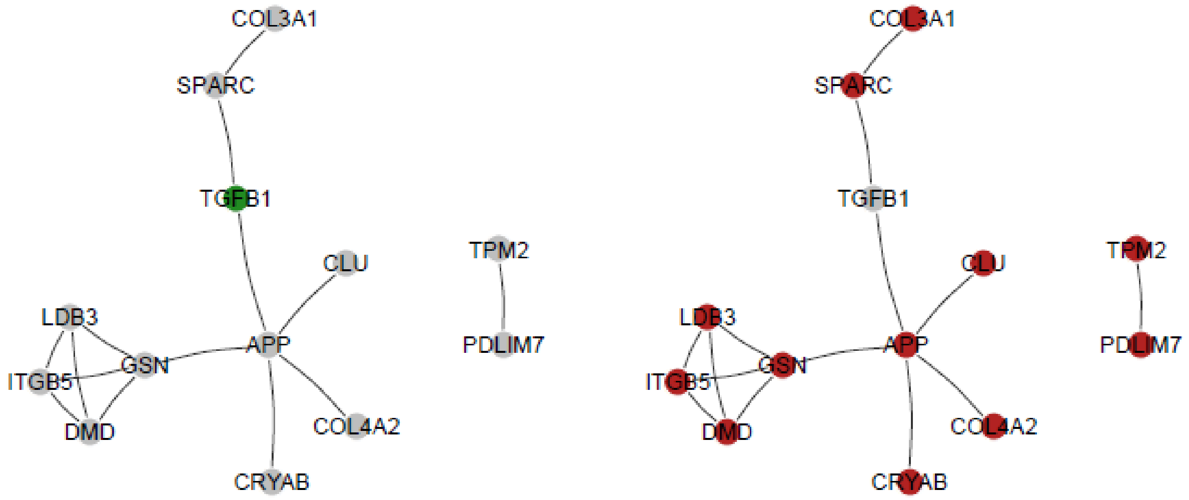

Figure S3k. APICAL JUNCTION

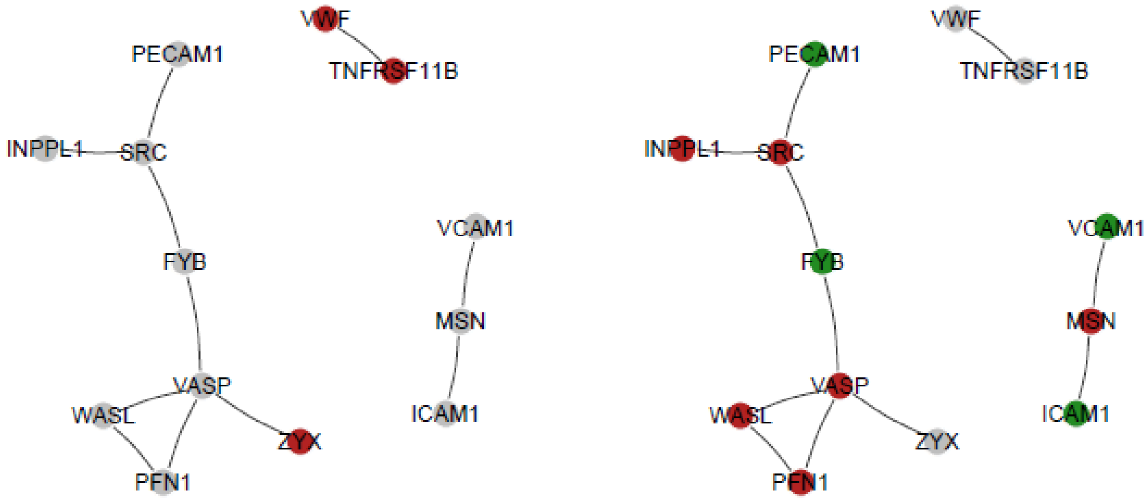

Figure S3l. MYC TARGETS V1

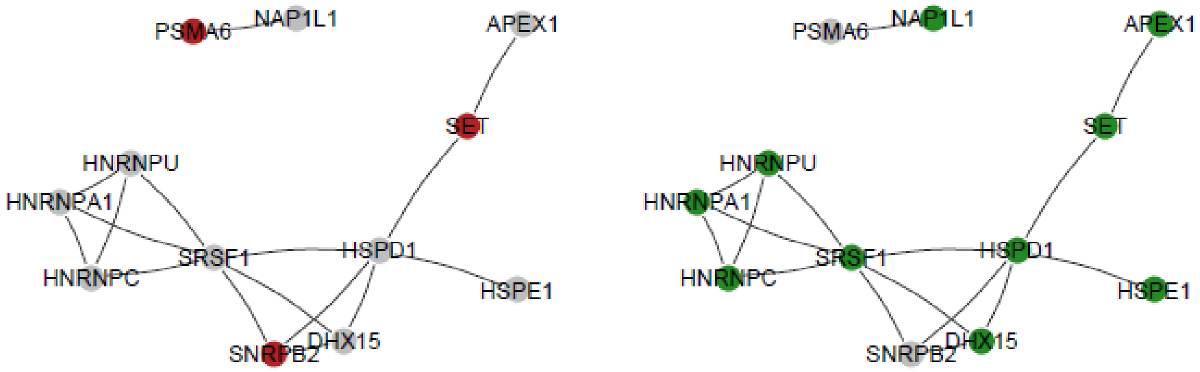

Figure S4

EM view of EC junctions in BAV-D (a-b) and TAV-D (c). Extensive degradation at EC junction (a) and stress fiber formation adjacent to late lysosomes (b) in BAV compared to TAV patients with dilated aortas. SF, stress fibers; LL, late lysosomes. Patients: N=2 in each group.

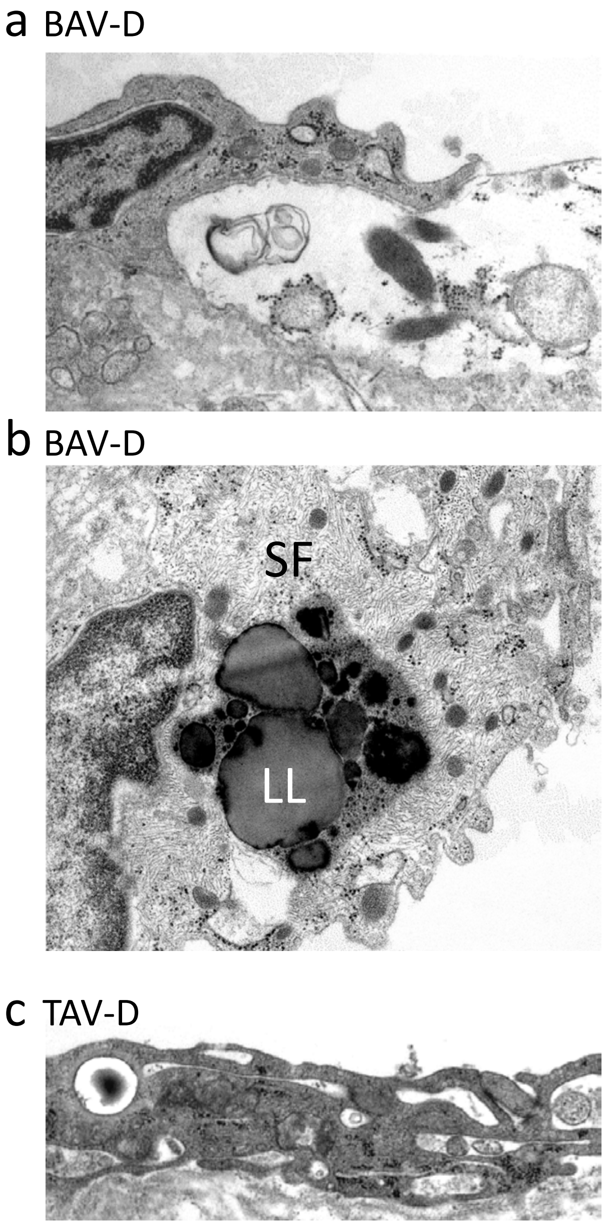

Figure S5

Staining of neovascularization areas, as internal control. Pictures are taken from neovascularization areas of the same sections stained for localization and quantification of AJ protein, CDH5, CDH5-P, in intima, Scale bar= 50µm, Patients: N=7-9 in each group, PTPRB N=3 in each group.

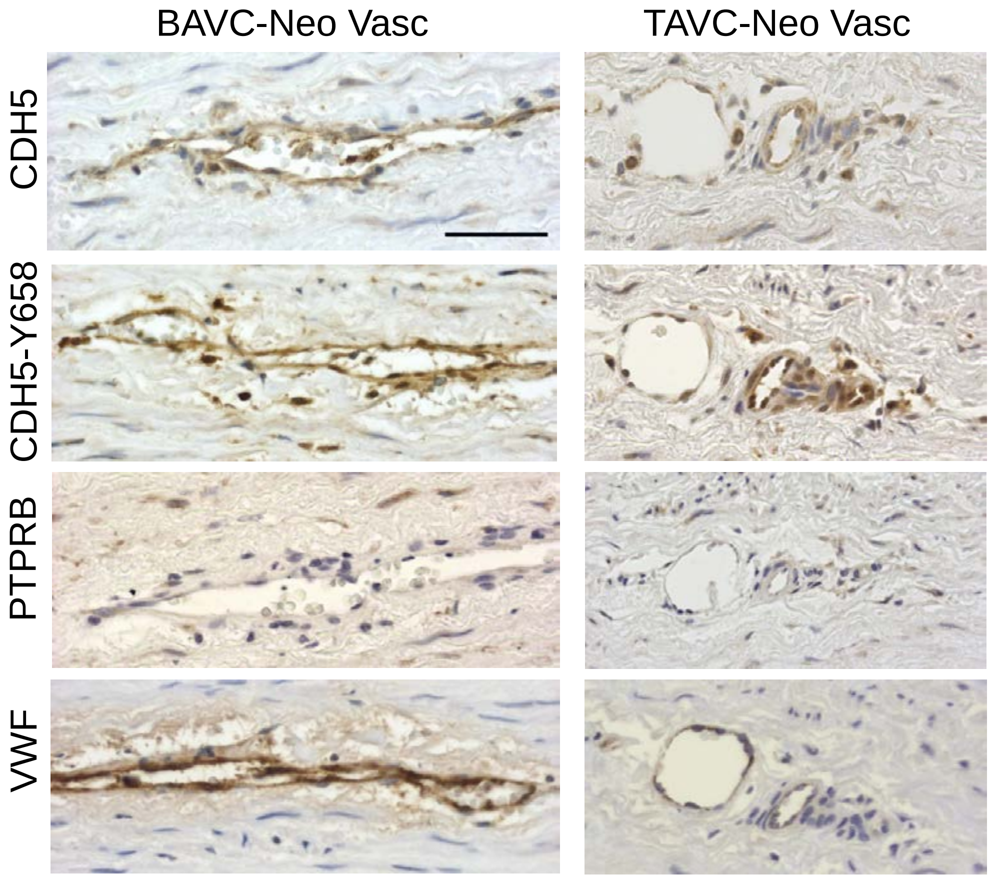

Figure S6

Comparison between BAV-ND (a) and TAV-ND (b) patients for relative distribution of PTPRB and CDH5-Y658, using serial sections. Scale bar= 50µm, Patients: N=3 in each group.

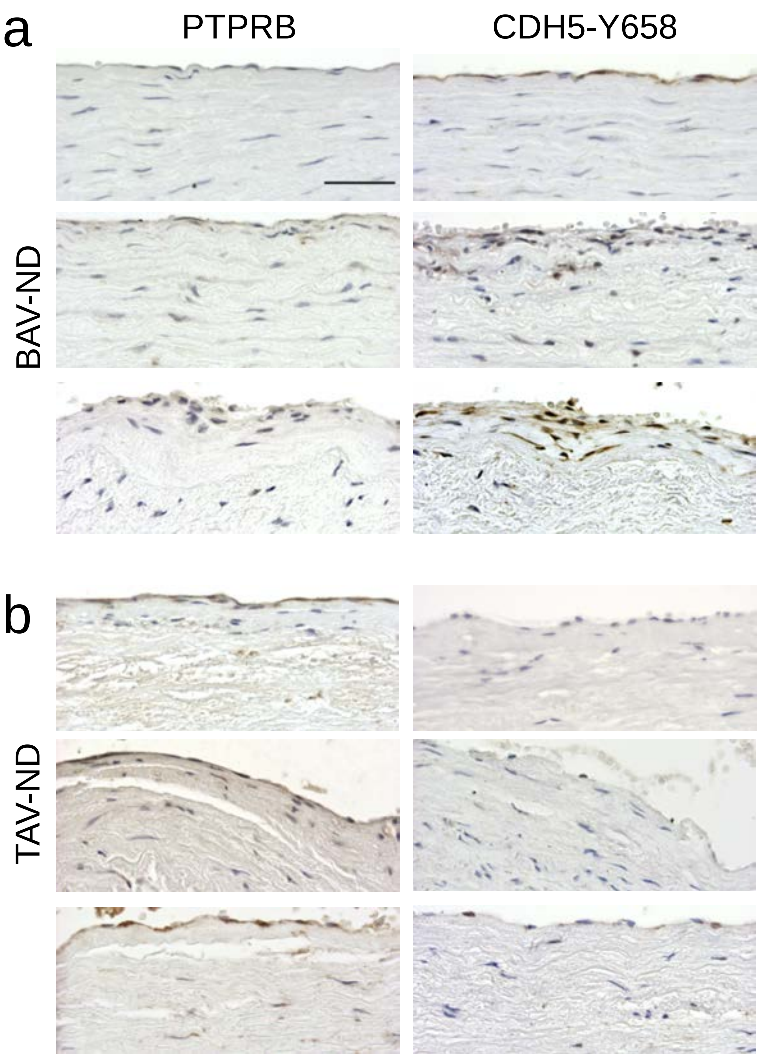

Figure S7

Examination of the connection between proliferative state and cell-cell junctions. We stained consecutive sections of a BAV-ND patient with high intimal proliferative activity, for CDH5, Phos-CDH5-Y658, PTPRB phosphatase, ZEB1 and KI67. The results further strengthened that the increased cell division in intimal layer, corresponded to the strong staining of anti-Phos-CDH5-Y658 and decreased PTPRB phosphatase activity, increased ZEB1 staining, implying that the higher cell proliferation, activation of EMT transcription factor and instability of the cell junctions are related and coordinated processes in BAV-ND, Scale bar= 50μm.

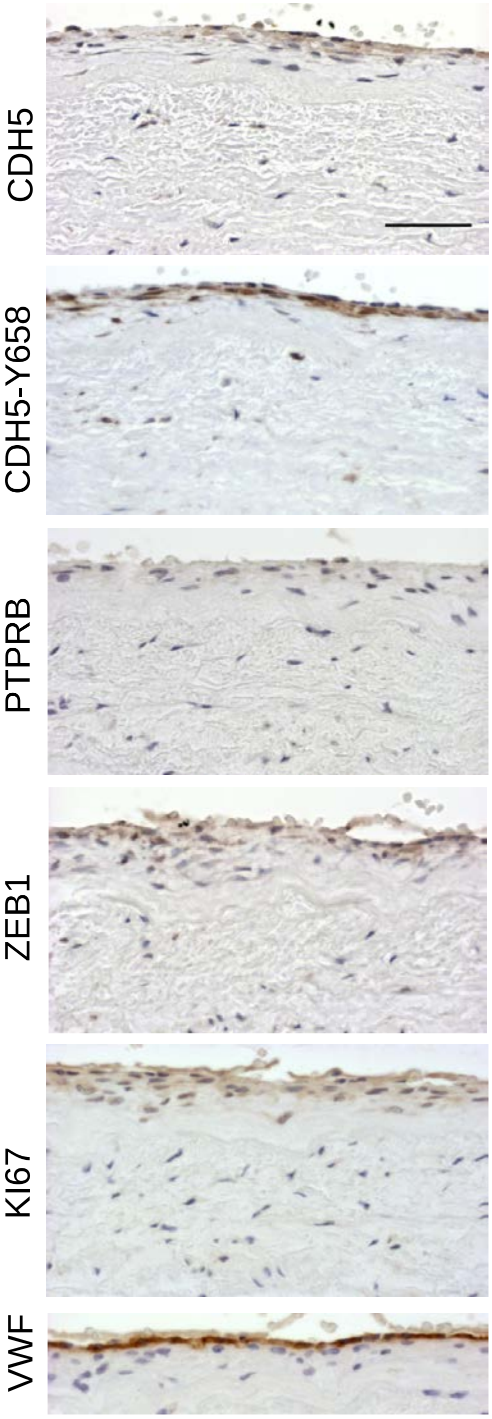

Figure S8:

Decreased and increased expressions of MFNG and MYLK, respectively, in BAV-ND. Western blot analysis of 20 µg protein extract from TAV (T) and BAV (B) non-dilated aorta incubated with (a) anti-MFNG antibody and (b) quantification of MFNG/Actinβ ratio (student's t-test); (c) anti-MYLK, (d) quantification MYLK/Actinβ ratio (student's t-test).

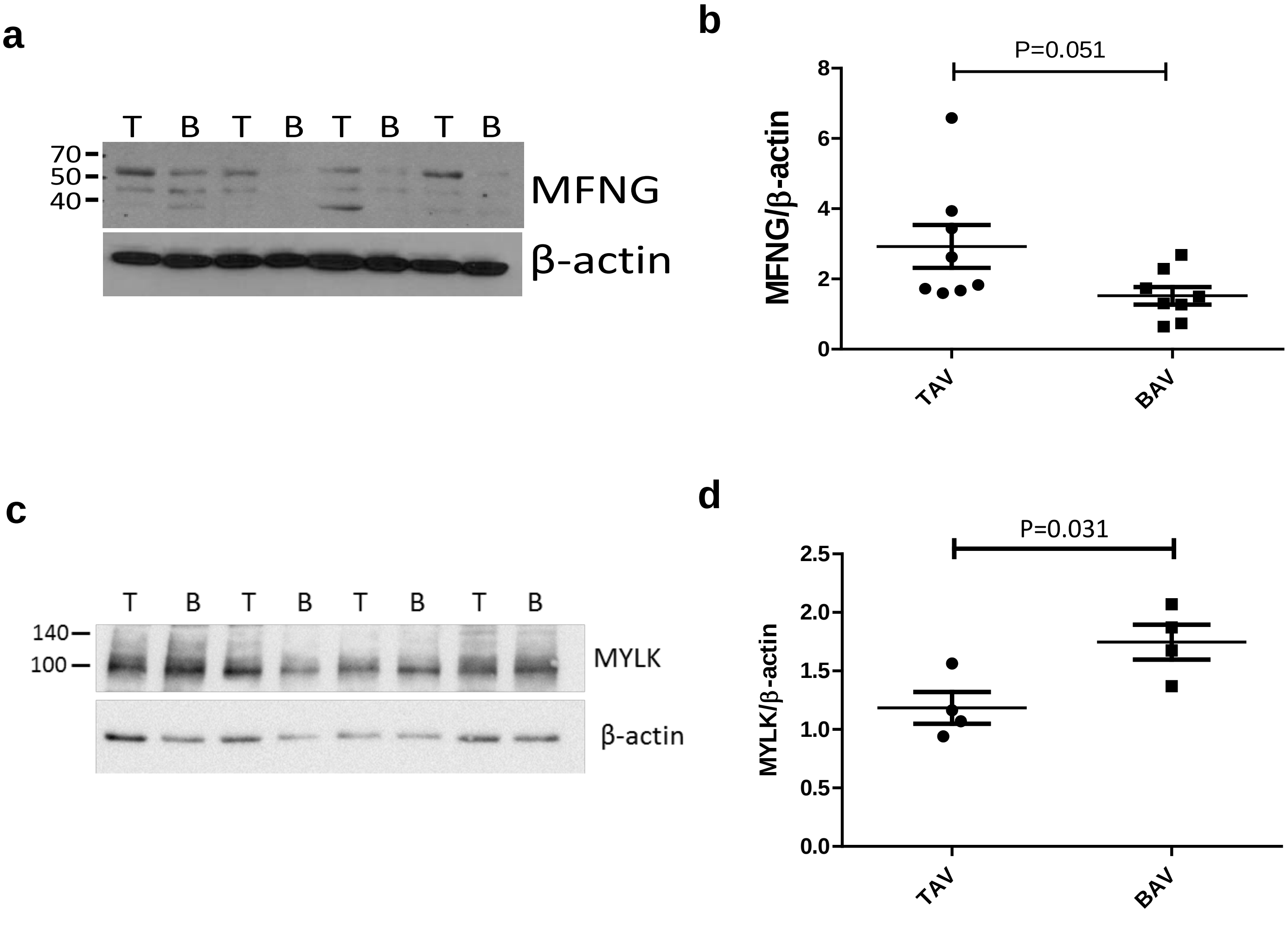

Figure S9

Internal thoracic arteries stained with antibodies used for aorta. Cell junction proteins, Scale bar= 50µm, Patients: N=7 in each group.

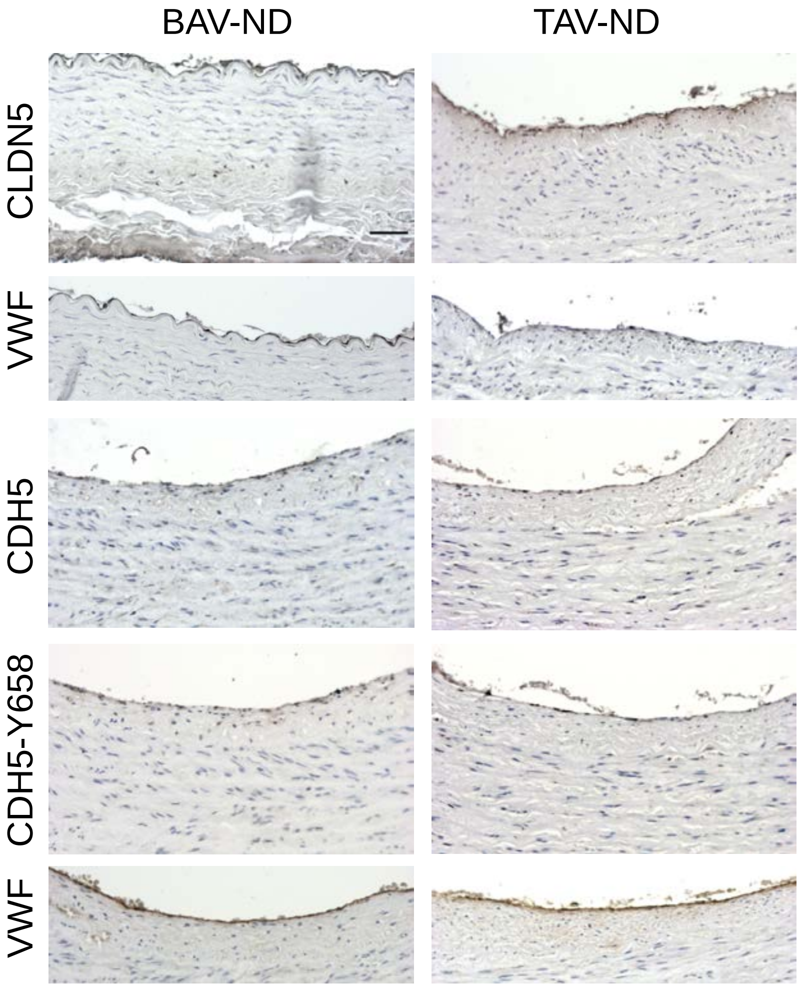

Figure S10

Internal thoracic arteries stained with antibodies used for aorta. RHOA pathway kinases, Scale bar= 50µm, Patients: N=7 in each group.

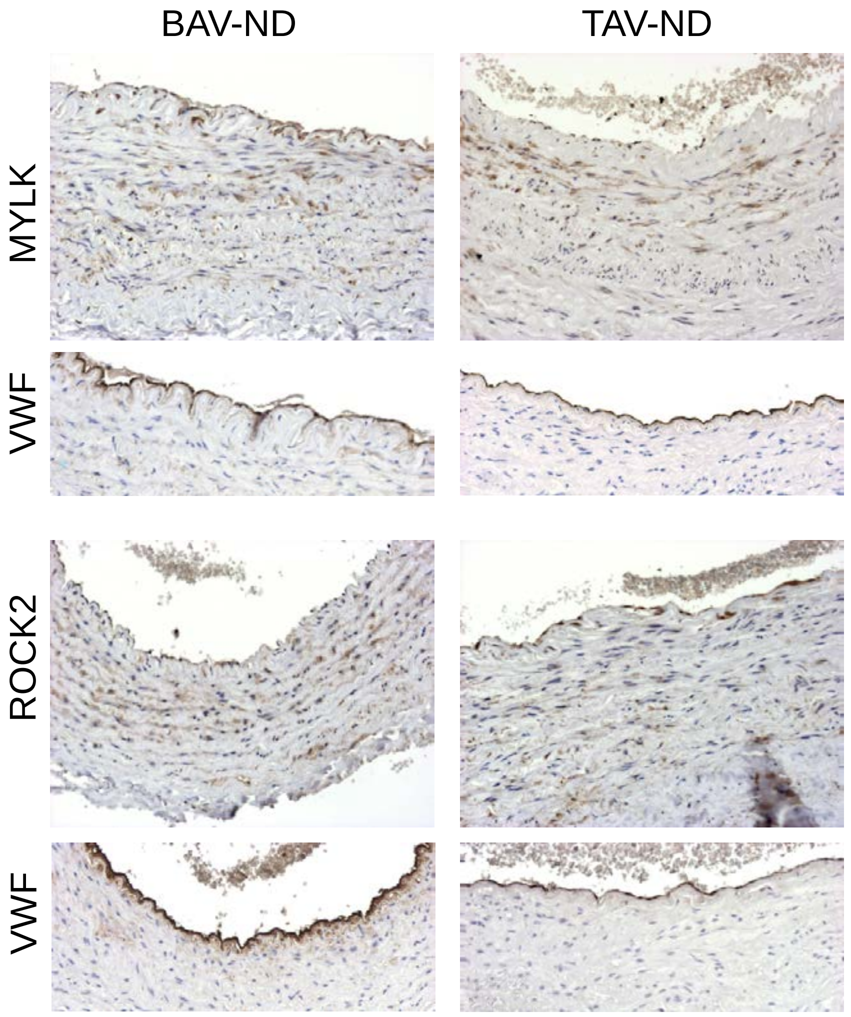

Figure S11

Internal thoracic arteries stained with antibodies used for aorta. RHOA pathway, Protein expression of Myosin II subunits MYL9, MYL9-P and MYL6, Scale bar= 50µm, Patients: N=7 in each group.

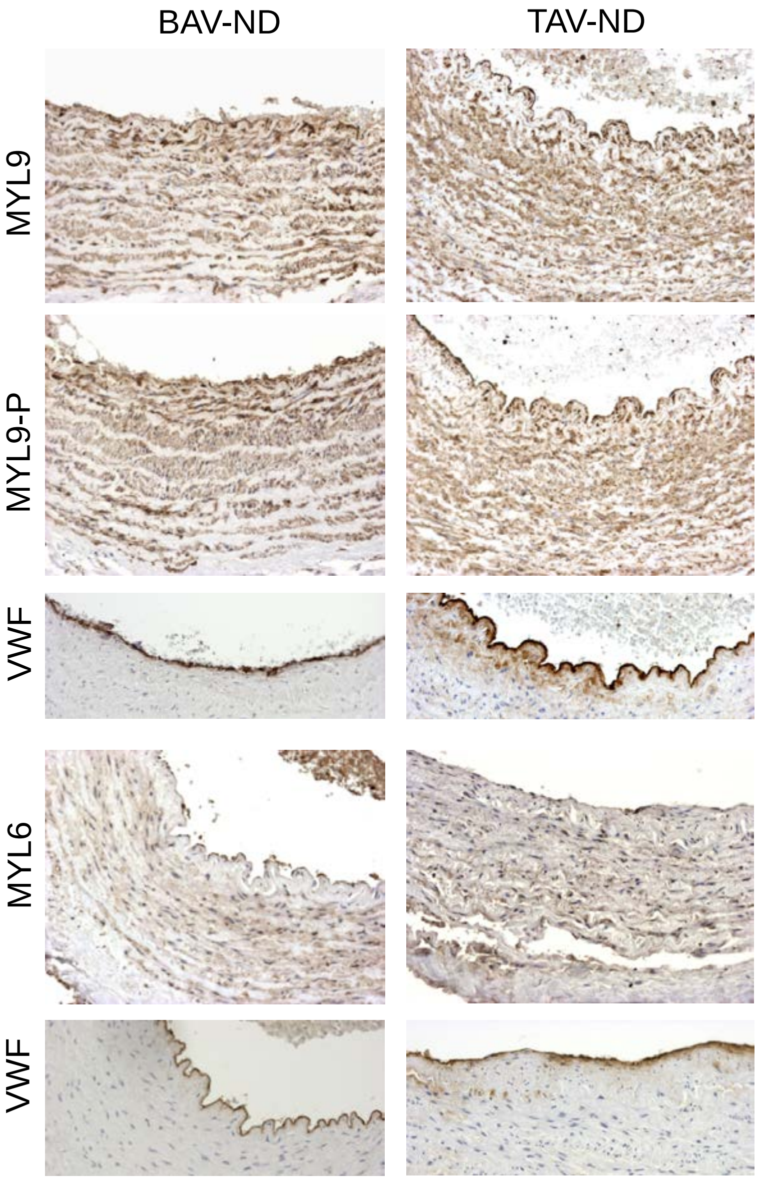

Figure S12

Internal thoracic arteries stained with antibodies used for aorta. NOCH pathway, Scale bar= 50µm, Patients: N=7 in each group.

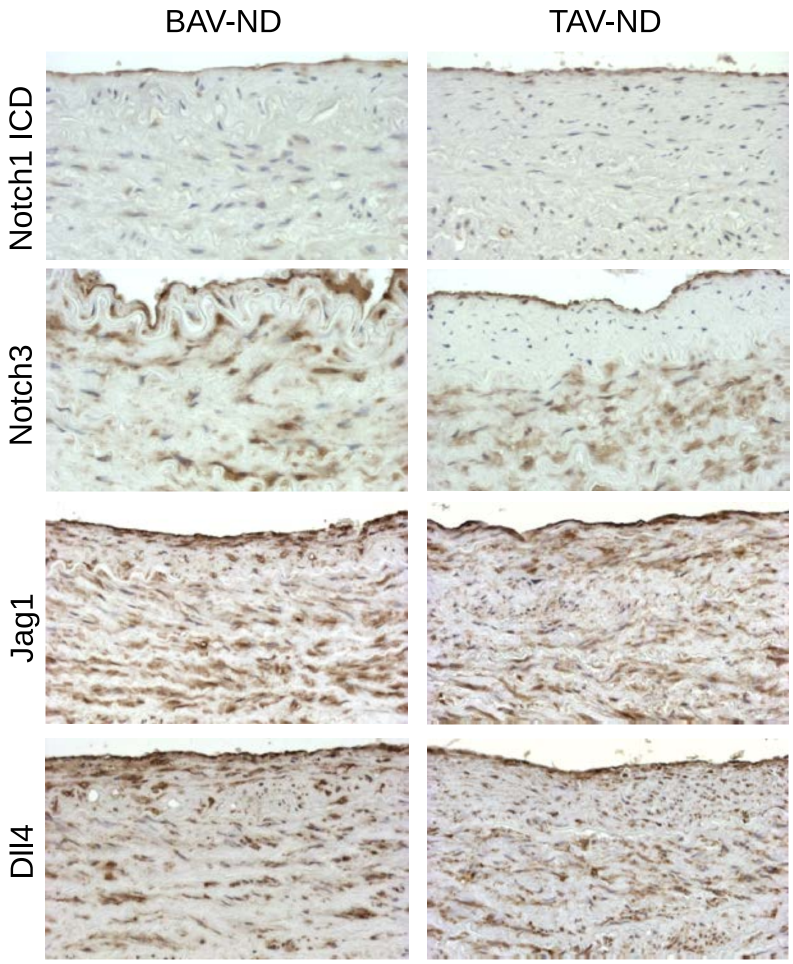

References

1 Jackson, V. *et al.* Bicuspid aortic valve leaflet morphology in relation to aortic root morphology: a study of 300 patients undergoing open-heart surgery. *European journal of cardio-thoracic surgery : official journal of the European Association for Cardio-thoracic Surgery* **40**, e118-124, doi:10.1016/j.ejcts.2011.04.014 (2011).

2 Kjellqvist, S. *et al.* A combined proteomic and transcriptomic approach shows diverging molecular mechanisms in thoracic aortic aneurysm development in patients with tricuspid- and bicuspid aortic valve. *Molecular & cellular proteomics : MCP* **12**, 407-425, doi:10.1074/mcp.M112.021873 (2013).

3 Branca, R. M. *et al.* HiRIEF LC-MS enables deep proteome coverage and unbiased proteogenomics. *Nat Methods* **11**, 59-62, doi:10.1038/nmeth.2732 (2014).

4 Maleki, S. *et al.* Identification of a novel flow-mediated gene expression signature in patients with bicuspid aortic valve. *J Mol Med (Berl)* **91**, 129-139, doi:10.1007/s00109-012-0942-8 (2013).

5 Wagsater, D. *et al.* Impaired collagen biosynthesis and cross-linking in aorta of patients with bicuspid aortic valve. *Journal of the American Heart Association* **2**, e000034, doi:10.1161/JAHA.112.000034 (2013).

6 Braniste, V. *et al.* The gut microbiota influences blood-brain barrier permeability in mice. *Sci Transl Med* **6**, 263ra158, doi:10.1126/scitranslmed.3009759 (2014).

7 Jackson, J. E. *A User's Guide to Principal Components*. (Wiley, New York 1991).

8 Trygg, J. & Wold, S. Orthogonal projections to latent structures (O-PLS). *J Chemometr* **16**, 119-128, doi:DOI 10.1002/cem.695 (2002).

9 Martens, H., Hoy, M., Westad, F., Folkenberg, D. & Martens, M. Analysis of designed experiments by stabilised PLS Regression and jack-knifing. *Chemometr Intell Lab* **58**, 151-170, doi:Doi 10.1016/S0169-7439(01)00157-5 (2001).

10 Wold, S. Cross-Validatory Estimation of Number of Components in Factor and Principal Components Models. *Technometrics* **20**, 397-405, doi:Doi 10.2307/1267639 (1978).

11 Folkersen, L. *et al.* Unraveling divergent gene expression profiles in bicuspid and tricuspid aortic valve patients with thoracic aortic dilatation: the ASAP study. *Mol Med* **17**, 1365-1373, doi:10.2119/molmed.2011.00286 (2011).

12 Subramanian, A. *et al.* Gene set enrichment analysis: a knowledge-based approach for interpreting genome-wide expression profiles. *Proc Natl Acad Sci U S A* **102**, 15545-15550, doi:10.1073/pnas.0506580102 (2005).

13 Peri, S. *et al.* Human protein reference database as a discovery resource for proteomics. *Nucleic Acids Res* **32**, D497-501, doi:10.1093/nar/gkh070 (2004).

14 Corallino, S., Malabarba, M. G., Zobel, M., Di Fiore, P. P. & Scita, G. Epithelial-to-Mesenchymal Plasticity Harnesses Endocytic Circuitries. *Front Oncol* **5**, 45, doi:10.3389/fonc.2015.00045 (2015).

15 Mamidi, S., Hone, S. & Kirschfink, M. The complement system in cancer: Ambivalence between tumour destruction and promotion. *Immunobiology*, doi:10.1016/j.imbio.2015.11.008 (2015).

16 Holecek, M. Relation between glutamine, branched-chain amino acids, and protein metabolism. *Nutrition* **18**, 130-133 (2002).

17 Lee, B. Y., Timpson, P., Horvath, L. G. & Daly, R. J. FAK signaling in human cancer as a target for therapeutics. *Pharmacol Ther* **146**, 132-149, doi:10.1016/j.pharmthera.2014.10.001 (2015).

18 Twum-Ampofo, J., Fu, D. X., Passaniti, A., Hussain, A. & Siddiqui, M. M. Metabolic targets for potential prostate cancer therapeutics. *Curr Opin Oncol*, doi:10.1097/CCO.0000000000000276 (2016).

19 Lane, A. N. & Fan, T. W. Regulation of mammalian nucleotide metabolism and biosynthesis. *Nucleic Acids Res* **43**, 2466-2485, doi:10.1093/nar/gkv047 (2015).

20 Alanko, J. & Ivaska, J. Endosomes: Emerging Platforms for Integrin-Mediated FAK Signalling. *Trends Cell Biol*, doi:10.1016/j.tcb.2016.02.001 (2016).

21 Long, J. P., Li, X. N. & Zhang, F. Targeting metabolism in breast cancer: How far we can go? *World J Clin Oncol* **7**, 122-130, doi:10.5306/wjco.v7.i1.122 (2016).

22 Ramadan, S. *et al.* Use of in vivo two-dimensional MR spectroscopy to compare the biochemistry of the human brain to that of glioblastoma. *Radiology* **259**, 540-549, doi:10.1148/radiol.11101123 (2011).

23 Yang, Y. *et al.* Mitochondria and Mitochondrial ROS in Cancer: Novel Targets for Anticancer Therapy. *J Cell Physiol*, doi:10.1002/jcp.25349 (2016).

24 Aberg, M., Eriksson, O. & Siegbahn, A. Tissue Factor Noncoagulant Signaling: Mechanisms and Implications for Cell Migration and Apoptosis. *Semin Thromb Hemost* **41**, 691-699, doi:10.1055/s-0035-1564046 (2015).

25 Kao, S. H., Wu, K. J. & Lee, W. H. Hypoxia, Epithelial-Mesenchymal Transition, and TET-Mediated Epigenetic Changes. *J Clin Med* **5**, doi:10.3390/jcm5020024 (2016).

26 Baig, S. *et al.* Potential of apoptotic pathway-targeted cancer therapeutic research: Where do we stand? *Cell death & disease* **7**, e2058, doi:10.1038/cddis.2015.275 (2016).

27 Haissaguerre, M., Saucisse, N. & Cota, D. Influence of mTOR in energy and metabolic homeostasis. *Mol Cell Endocrinol* **397**, 67-77, doi:10.1016/j.mce.2014.07.015 (2014).

28 Morris, H. T. & Machesky, L. M. Actin cytoskeletal control during epithelial to mesenchymal transition: focus on the pancreas and intestinal tract. *Br J Cancer* **112**, 613-620, doi:10.1038/bjc.2014.658 (2015).

29 Reymond, N., d'Agua, B. B. & Ridley, A. J. Crossing the endothelial barrier during metastasis. *Nat Rev Cancer* **13**, 858-870, doi:10.1038/nrc3628 (2013).

30 Wojtuszkiewicz, A. *et al.* Pre-mRNA splicing in cancer: the relevance in oncogenesis, treatment and drug resistance. *Expert Opin Drug Metab Toxicol* **11**, 673-689, doi:10.1517/17425255.2015.993316 (2015).

31 Joshi, T., Butchar, J. P. & Tridandapani, S. Fcgamma receptor signaling in phagocytes. *Int J Hematol* **84**, 210-216, doi:10.1532/IJH97.06140 (2006).

32 Zihni, C. & Terry, S. J. RhoGTPase signalling at epithelial tight junctions: Bridging the GAP between polarity and cancer. *Int J Biochem Cell Biol* **64**, 120-125, doi:10.1016/j.biocel.2015.02.020 (2015).

33 Cosenza, M. R. & Kramer, A. Centrosome amplification, chromosomal instability and cancer: mechanistic, clinical and therapeutic issues. *Chromosome Res* **24**, 105-126, doi:10.1007/s10577-015-9505-5 (2016).

34 Tai, S., Hu, X. Q., Peng, D. Q., Zhou, S. H. & Zheng, X. L. The roles of autophagy in vascular smooth muscle cells. *Int J Cardiol* **211**, 1-6, doi:10.1016/j.ijcard.2016.02.128 (2016).

35 Hsieh, A. L., Walton, Z. E., Altman, B. J., Stine, Z. E. & Dang, C. V. MYC and metabolism on the path to cancer. *Semin Cell Dev Biol* **43**, 11-21, doi:10.1016/j.semcdb.2015.08.003 (2015).

36 Jiang, J. *et al.* Multifunctional proteins bridge mitosis with motility and cancer with inflammation and arthritis. *ScientificWorldJournal* **10**, 1244-1257, doi:10.1100/tsw.2010.141 (2010).

37 Jimenez, G., Shvartsman, S. Y. & Paroush, Z. The Capicua repressor--a general sensor of RTK signaling in development and disease. *J Cell Sci* **125**, 1383-1391, doi:10.1242/jcs.092965 (2012).

38 Merlo, P. & Cecconi, F. XIAP: inhibitor of two worlds. *EMBO J* **32**, 2187-2188, doi:10.1038/emboj.2013.152 (2013).

39 Obexer, P. & Ausserlechner, M. J. X-linked inhibitor of apoptosis protein - a critical death resistance regulator and therapeutic target for personalized cancer therapy. *Front Oncol* **4**, 197, doi:10.3389/fonc.2014.00197 (2014).

40 Chamulitrat, W., Sattayakhom, A., Herold-Mende, C. & Stremmel, W. Human papillomavirus 16 E6/E7-immortalized human gingival keratinocytes with epithelial mesenchymal transition acquire increased expression of cIAP-1, Bclx and p27(Kip1). *Exp Dermatol* **18**, 1067-1069, doi:10.1111/j.1600-0625.2009.00888.x (2009).

41 Lee, J. & Kim, S. S. The function of p27 KIP1 during tumor development. *Exp Mol Med* **41**, 765-771, doi:10.3858/emm.2009.41.11.102 (2009).

42 Subramaniam, D., Thombre, R., Dhar, A. & Anant, S. DNA methyltransferases: a novel target for prevention and therapy. *Front Oncol* **4**, 80, doi:10.3389/fonc.2014.00080 (2014).

43 Jiang, Y. Z. *et al.* Hemodynamic disturbed flow induces differential DNA methylation of endothelial Kruppel-Like Factor 4 promoter in vitro and in vivo. *Circ Res* **115**, 32-43, doi:10.1161/CIRCRESAHA.115.303883 (2014).

44 Suzuki, N. *et al.* Calcium-dependent structural changes in human reticulocalbin-1. *J Biochem* **155**, 281-293, doi:10.1093/jb/mvu003 (2014).

45 Feighery, R., Maguire, P., Ryan, M. P. & McMorrow, T. A proteomic approach to immune-mediated epithelial-mesenchymal transition. *Proteomics Clin Appl* **2**, 1110-1117, doi:10.1002/prca.200780148 (2008).

46 Amatschek, S. *et al.* Tissue-wide expression profiling using cDNA subtraction and microarrays to identify tumor-specific genes. *Cancer Res* **64**, 844-856 (2004).

47 Gomes, A. V. Genetics of proteasome diseases. *Scientifica (Cairo)* **2013**, 637629, doi:10.1155/2013/637629 (2013).

48 Canal, F. *et al.* A kinome siRNA screen identifies HGS as a potential target for liver cancers with oncogenic mutations in CTNNB1. *BMC Cancer* **15**, 1020, doi:10.1186/s12885-015-2037-8 (2015).

49 Kojima, K. *et al.* ESCRT-0 protein hepatocyte growth factor-regulated tyrosine kinase substrate (Hrs) is targeted to endosomes independently of signal-transducing adaptor molecule (STAM) and the complex formation with STAM promotes its endosomal dissociation. *J Biol Chem* **289**, 33296-33310, doi:10.1074/jbc.M114.578245 (2014).

50 Miller-Delaney, S. F., Lieberam, I., Murphy, P. & Mitchell, K. J. Plxdc2 is a mitogen for neural progenitors. *PloS one* **6**, e14565, doi:10.1371/journal.pone.0014565 (2011).

51 Carson-Walter, E. B. *et al.* Cell surface tumor endothelial markers are conserved in mice and humans. *Cancer Res* **61**, 6649-6655 (2001).

52 Iglesias-Gato, D. *et al.* OTUB1 de-ubiquitinating enzyme promotes prostate cancer cell invasion in vitro and tumorigenesis in vivo. *Mol Cancer* **14**, 8, doi:10.1186/s12943-014-0280-2 (2015).

53 Zhou, Y. *et al.* OTUB1 promotes metastasis and serves as a marker of poor prognosis in colorectal cancer. *Mol Cancer* **13**, 258, doi:10.1186/1476-4598-13-258 (2014).

54 Rozanov, D. V. *et al.* Molecular signature of MT1-MMP: transactivation of the downstream universal gene network in cancer. *Cancer Res* **68**, 4086-4096, doi:10.1158/0008-5472.CAN-07-6458 (2008).

55 Falsone, S. F., Gesslbauer, B., Tirk, F., Piccinini, A. M. & Kungl, A. J. A proteomic snapshot of the human heat shock protein 90 interactome. *FEBS Lett* **579**, 6350-6354, doi:10.1016/j.febslet.2005.10.020 (2005).

56 O'Rourke, S. M., Dorfman, M. D., Carter, J. C. & Bowerman, B. Dynein modifiers in C. elegans: light chains suppress conditional heavy chain mutants. *PLoS Genet* **3**, e128, doi:10.1371/journal.pgen.0030128 (2007).

57 Kimura, N., Okabayashi, S. & Ono, F. Dynein dysfunction disrupts beta-amyloid clearance in astrocytes through endocytic disturbances. *Neuroreport* **25**, 514-520, doi:10.1097/WNR.0000000000000124 (2014).

58 Xu, M. *et al.* Regulation of autophagic flux by dynein-mediated autophagosomes trafficking in mouse coronary arterial myocytes. *Biochim Biophys Acta* **1833**, 3228-3236, doi:10.1016/j.bbamcr.2013.09.015 (2013).

59 Besse, A. *et al.* The GABA transaminase, ABAT, is essential for mitochondrial nucleoside metabolism. *Cell metabolism* **21**, 417-427, doi:10.1016/j.cmet.2015.02.008 (2015).

60 Mauxion, F., Preve, B. & Seraphin, B. C2ORF29/CNOT11 and CNOT10 form a new module of the CCR4-NOT complex. *RNA Biol* **10**, 267-276, doi:10.4161/rna.23065 (2013).

61 Lin, Y. F., Yang, J. & Rosen, B. P. ArsD: an As(III) metallochaperone for the ArsAB As(III)-translocating ATPase. *J Bioenerg Biomembr* **39**, 453-458, doi:10.1007/s10863-007-9113-y (2007).

62 Lehtinen, L. *et al.* High-throughput RNAi screening for novel modulators of vimentin expression identifies MTHFD2 as a regulator of breast cancer cell migration and invasion. *Oncotarget* **4**, 48-63 (2013).

63 Nilsson, R. *et al.* Metabolic enzyme expression highlights a key role for MTHFD2 and the mitochondrial folate pathway in cancer. *Nat Commun* **5**, 3128, doi:10.1038/ncomms4128 (2014).

64 Smith, M. A. *et al.* A zyxin-mediated mechanism for actin stress fiber maintenance and repair. *Dev Cell* **19**, 365-376, doi:10.1016/j.devcel.2010.08.008 (2010).

65 Smith, M. A., Hoffman, L. M. & Beckerle, M. C. LIM proteins in actin cytoskeleton mechanoresponse. *Trends Cell Biol* **24**, 575-583, doi:10.1016/j.tcb.2014.04.009 (2014).

66 Kim, D. H. & Wirtz, D. Predicting how cells spread and migrate: focal adhesion size does matter. *Cell Adh Migr* **7**, 293-296, doi:10.4161/cam.24804 (2013).

67 Mise, N. *et al.* Zyxin is a transforming growth factor-beta (TGF-beta)/Smad3 target gene that regulates lung cancer cell motility via integrin alpha5beta1. *J Biol Chem* **287**, 31393-31405, doi:10.1074/jbc.M112.357624 (2012).

68 Richmond, J., Tuzova, M., Cruikshank, W. & Center, D. Regulation of cellular processes by interleukin-16 in homeostasis and cancer. *J Cell Physiol* **229**, 139-147, doi:10.1002/jcp.24441 (2014).

69 Mertz, J. *et al.* Sequential Elution Interactome Analysis of the Mind Bomb 1 Ubiquitin Ligase Reveals a Novel Role in Dendritic Spine Outgrowth. *Molecular & cellular proteomics : MCP* **14**, 1898-1910, doi:10.1074/mcp.M114.045898 (2015).

70 Gan-Or, Z., Dion, P. A. & Rouleau, G. A. Genetic perspective on the role of the autophagy-lysosome pathway in Parkinson disease. *Autophagy* **11**, 1443-1457, doi:10.1080/15548627.2015.1067364 (2015).

71 Ohi, Y. *et al.* Incomplete DNA methylation underlies a transcriptional memory of somatic cells in human iPS cells. *Nat Cell Biol* **13**, 541-549, doi:10.1038/ncb2239 (2011).

72 Cai, L. Y. *et al.* Identification of PRTFDC1 silencing and aberrant promoter methylation of GPR150, ITGA8 and HOXD11 in ovarian cancers. *Life Sci* **80**, 1458-1465, doi:10.1016/j.lfs.2007.01.015 (2007).

73 Sweetser, D. A. *et al.* Delineation of the minimal commonly deleted segment and identification of candidate tumor-suppressor genes in del(9q) acute myeloid leukemia. *Genes Chromosomes Cancer* **44**, 279-291, doi:10.1002/gcc.20236 (2005).

74 Kaartokallio, T., Cervera, A., Kyllonen, A., Laivuori, K. & Group, F. C. I. Gene expression profiling of pre-eclamptic placentae by RNA sequencing. *Sci Rep* **5**, 14107, doi:10.1038/srep14107 (2015).

75 Jiang, P., Du, W., Mancuso, A., Wellen, K. E. & Yang, X. Reciprocal regulation of p53 and malic enzymes modulates metabolism and senescence. *Nature* **493**, 689-693, doi:10.1038/nature11776 (2013).

76 Zheng, F. J. *et al.* Repressing malic enzyme 1 redirects glucose metabolism, unbalances the redox state, and attenuates migratory and invasive abilities in nasopharyngeal carcinoma cell lines. *Chin J Cancer* **31**, 519-531, doi:10.5732/cjc.012.10088 (2012).

77 Rosenbluh, J. *et al.* beta-Catenin-driven cancers require a YAP1 transcriptional complex for survival and tumorigenesis. *Cell* **151**, 1457-1473, doi:10.1016/j.cell.2012.11.026 (2012).

78 Zhang, H. *et al.* Yap1 is required for endothelial to mesenchymal transition of the atrioventricular cushion. *J Biol Chem* **289**, 18681-18692, doi:10.1074/jbc.M114.554584 (2014).

79 Tschaharganeh, D. F. *et al.* Yes-associated protein up-regulates Jagged-1 and activates the Notch pathway in human hepatocellular carcinoma. *Gastroenterology* **144**, 1530-1542 e1512, doi:10.1053/j.gastro.2013.02.009 (2013).

80 Lamar, J. M. *et al.* The Hippo pathway target, YAP, promotes metastasis through its TEAD-interaction domain. *Proc Natl Acad Sci U S A* **109**, E2441-2450, doi:10.1073/pnas.1212021109 (2012).

81 Kato, K., Okuwaki, M. & Nagata, K. Role of Template Activating Factor-I as a chaperone in linker histone dynamics. *J Cell Sci* **124**, 3254-3265, doi:10.1242/jcs.083139 (2011).

82 Cervoni, N., Detich, N., Seo, S. B., Chakravarti, D. & Szyf, M. The oncoprotein Set/TAF-1beta, an inhibitor of histone acetyltransferase, inhibits active demethylation of DNA, integrating DNA methylation and transcriptional silencing. *J Biol Chem* **277**, 25026-25031, doi:10.1074/jbc.M202256200 (2002).

83 Chaineau, M., Ioannou, M. S. & McPherson, P. S. Rab35: GEFs, GAPs and effectors. *Traffic* **14**, 1109-1117, doi:10.1111/tra.12096 (2013).

84 Iwamura, A., Fukami, T., Higuchi, R., Nakajima, M. & Yokoi, T. Human alpha/beta hydrolase domain containing 10 (ABHD10) is responsible enzyme for deglucuronidation of mycophenolic acid acyl-glucuronide in liver. *J Biol Chem* **287**, 9240-9249, doi:10.1074/jbc.M111.271288 (2012).

85 Bailey, M. J. & Dickinson, R. G. Acyl glucuronide reactivity in perspective: biological consequences. *Chem Biol Interact* **145**, 117-137 (2003).

86 Vaccari, T. & Bilder, D. The Drosophila tumor suppressor vps25 prevents nonautonomous overproliferation by regulating notch trafficking. *Dev Cell* **9**, 687-698, doi:10.1016/j.devcel.2005.09.019 (2005).

87 Le Borgne, R. Regulation of Notch signalling by endocytosis and endosomal sorting. *Curr Opin Cell Biol* **18**, 213-222, doi:10.1016/j.ceb.2006.02.011 (2006).

88 Rusten, T. E. *et al.* ESCRTs and Fab1 regulate distinct steps of autophagy. *Curr Biol* **17**, 1817-1825, doi:10.1016/j.cub.2007.09.032 (2007).

89 Fedorova, O. A. *et al.* Proteomic analysis of the 20S proteasome (PSMA3)-interacting proteins reveals a functional link between the proteasome and mRNA metabolism. *Biochem Biophys Res Commun* **416**, 258-265, doi:10.1016/j.bbrc.2011.10.126 (2011).

90 Head, R. A. *et al.* Clinical and genetic spectrum of pyruvate dehydrogenase deficiency: dihydrolipoamide acetyltransferase (E2) deficiency. *Ann Neurol* **58**, 234-241, doi:10.1002/ana.20550 (2005).

91 Sepe, M. *et al.* A regulatory mechanism involving TBP-1/Tat-Binding Protein 1 and Akt/PKB in the control of cell proliferation. *PloS one* **6**, e22800, doi:10.1371/journal.pone.0022800 (2011).

92 Pollice, A. *et al.* TBP-1 protects the human oncosuppressor p14ARF from proteasomal degradation. *Oncogene* **26**, 5154-5162, doi:10.1038/sj.onc.1210313 (2007).

93 Park, B. W. *et al.* Induction of the Tat-binding protein 1 gene accompanies the disabling of oncogenic erbB receptor tyrosine kinases. *Proc Natl Acad Sci U S A* **96**, 6434-6438 (1999).

94 Frey, C. R. *et al.* Mutations in adenosine deaminase-like (ADAL) protein confer resistance to the antiproliferative agents N6-cyclopropyl-PMEDAP and GS-9219. *Anticancer research* **33**, 1899-1912 (2013).

95 Jovov, B. *et al.* Differential gene expression between African American and European American colorectal cancer patients. *PloS one* **7**, e30168, doi:10.1371/journal.pone.0030168 (2012).

96 Kondo, A. *et al.* A new paxillin-binding protein, PAG3/Papalpha/KIAA0400, bearing an ADP-ribosylation factor GTPase-activating protein activity, is involved in paxillin recruitment to focal adhesions and cell migration. *Mol Biol Cell* **11**, 1315-1327 (2000).

97 Uchida, H., Kondo, A., Yoshimura, Y., Mazaki, Y. & Sabe, H. PAG3/Papalpha/KIAA0400, a GTPase-activating protein for ADP-ribosylation factor (ARF), regulates ARF6 in Fcgamma receptor-mediated phagocytosis of macrophages. *J Exp Med* **193**, 955-966 (2001).

98 Gotoh, M. *et al.* Comprehensive exploration of novel chimeric transcripts in clear cell renal cell carcinomas using whole transcriptome analysis. *Genes Chromosomes Cancer* **53**, 1018-1032, doi:10.1002/gcc.22211 (2014).

99 Cai, T. *et al.* Identification of differentially coexpressed genes in gonadotrope tumors and normal pituitary using bioinformatics methods. *Pathol Oncol Res* **20**, 375-380, doi:10.1007/s12253-013-9706-1 (2014).

100 Mou, Z., Tapper, A. R. & Gardner, P. D. The armadillo repeat-containing protein, ARMCX3, physically and functionally interacts with the developmental regulatory factor Sox10. *J Biol Chem* **284**, 13629-13640, doi:10.1074/jbc.M901177200 (2009).

101 Serrat, R. *et al.* The non-canonical Wnt/PKC pathway regulates mitochondrial dynamics through degradation of the arm-like domain-containing protein Alex3. *PloS one* **8**, e67773, doi:10.1371/journal.pone.0067773 (2013).

102 Grochola, L. F. *et al.* Recent natural selection identifies a genetic variant in a regulatory subunit of protein phosphatase 2A that associates with altered cancer risk and survival. *Clin Cancer Res* **15**, 6301-6308, doi:10.1158/1078-0432.CCR-09-0797 (2009).

103 Liu, X. *et al.* Downregulation of PPP2R5E expression by miR-23a suppresses apoptosis to facilitate the growth of gastric cancer cells. *FEBS Lett* **588**, 3160-3169, doi:10.1016/j.febslet.2014.05.068 (2014).

104 Cristobal, I. *et al.* Downregulation of PPP2R5E is a common event in acute myeloid leukemia that affects the oncogenic potential of leukemic cells. *Haematologica* **98**, e103-104, doi:10.3324/haematol.2013.084731 (2013).

105 Yang, R. *et al.* Functional genetic polymorphisms in PP2A subunit genes confer increased risks of lung cancer in southern and eastern Chinese. *PloS one* **8**, e77285, doi:10.1371/journal.pone.0077285 (2013).

106 Cristobal, I. *et al.* PP2A inhibition is a common event in colorectal cancer and its restoration using FTY720 shows promising therapeutic potential. *Mol Cancer Ther* **13**, 938-947, doi:10.1158/1535-7163.MCT-13-0150 (2014).

107 Offenhauser, N. *et al.* The eps8 family of proteins links growth factor stimulation to actin reorganization generating functional redundancy in the Ras/Rac pathway. *Mol Biol Cell* **15**, 91-98, doi:10.1091/mbc.E03-06-0427 (2004).

108 Colas, E. *et al.* Molecular markers of endometrial carcinoma detected in uterine aspirates. *Int J Cancer* **129**, 2435-2444, doi:10.1002/ijc.25901 (2011).

109 Yoshida, M. *et al.* RPAP3 splicing variant isoform 1 interacts with PIH1D1 to compose R2TP complex for cell survival. *Biochem Biophys Res Commun* **430**, 320-324, doi:10.1016/j.bbrc.2012.11.017 (2013).

110 Ni, L. *et al.* RPAP3 interacts with Reptin to regulate UV-induced phosphorylation of H2AX and DNA damage. *J Cell Biochem* **106**, 920-928, doi:10.1002/jcb.22073 (2009).

111 von Morgen, P., Horejsi, Z. & Macurek, L. Substrate recognition and function of the R2TP complex in response to cellular stress. *Front Genet* **6**, 69, doi:10.3389/fgene.2015.00069 (2015).

112 Bartke, T., Pohl, C., Pyrowolakis, G. & Jentsch, S. Dual role of BRUCE as an antiapoptotic IAP and a chimeric E2/E3 ubiquitin ligase. *Mol Cell* **14**, 801-811, doi:10.1016/j.molcel.2004.05.018 (2004).

113 Tang, W. *et al.* BIRC6 promotes hepatocellular carcinogenesis: interaction of BIRC6 with p53 facilitating p53 degradation. *Int J Cancer* **136**, E475-487, doi:10.1002/ijc.29194 (2015).

114 Luk, S. U. *et al.* The BIRC6 gene as a novel target for therapy of prostate cancer: dual targeting of inhibitors of apoptosis. *Oncotarget* **5**, 6896-6908 (2014).

115 Wang, L. *et al.* Expression and clinical significance of BIRC6 in human epithelial ovarian cancer. *Tumour Biol* **35**, 4891-4896, doi:10.1007/s13277-014-1641-6 (2014).

116 Dong, X. *et al.* Elevated expression of BIRC6 protein in non-small-cell lung cancers is associated with cancer recurrence and chemoresistance. *J Thorac Oncol* **8**, 161-170, doi:10.1097/JTO.0b013e31827d5237 (2013).

117 Goldenstein, H., Levy, N. S. & Levy, A. P. Haptoglobin genotype and its role in determining heme-iron mediated vascular disease. *Pharmacol Res* **66**, 1-6, doi:10.1016/j.phrs.2012.02.011 (2012).

118 Costacou, T. & Levy, A. P. Haptoglobin genotype and its role in diabetic cardiovascular disease. *J Cardiovasc Transl Res* **5**, 423-435, doi:10.1007/s12265-012-9361-z (2012).

119 Buttari, B. *et al.* Oxidized haemoglobin-driven endothelial dysfunction and immune cell activation: novel therapeutic targets for atherosclerosis. *Curr Med Chem* **20**, 4806-4814 (2013).

120 Schaer, C. A. *et al.* Mechanisms of haptoglobin protection against hemoglobin peroxidation triggered endothelial damage. *Cell Death Differ* **20**, 1569-1579, doi:10.1038/cdd.2013.113 (2013).

121 Park, D. S., Seo, J. H., Hong, M. & Choi, S. C. Role of the Rap2/TNIK kinase pathway in regulation of LRP6 stability for Wnt signaling. *Biochem Biophys Res Commun* **436**, 338-343, doi:10.1016/j.bbrc.2013.05.104 (2013).

122 Wu, J. X., Zhang, D. G., Zheng, J. N. & Pei, D. S. Rap2a is a novel target gene of p53 and regulates cancer cell migration and invasion. *Cell Signal* **27**, 1198-1207, doi:10.1016/j.cellsig.2015.02.026 (2015).

123 Wang, L. *et al.* Over-expression of Rap2a inhibits glioma migration and invasion by down-regulating p-AKT. *Cell Biol Int* **38**, 326-334, doi:10.1002/cbin.10213 (2014).

124 Dib, K., Axelsson, L. & Andersson, T. Beta2 integrins target Rap GTPases to the plasma membrane by means of degranulation. *Biochem Biophys Res Commun* **376**, 642-646, doi:10.1016/j.bbrc.2008.08.160 (2008).

125 Stratikos, E., Stamogiannos, A., Zervoudi, E. & Fruci, D. A role for naturally occurring alleles of endoplasmic reticulum aminopeptidases in tumor immunity and cancer pre-disposition. *Front Oncol* **4**, 363, doi:10.3389/fonc.2014.00363 (2014).

126 Gadalla, S. E. *et al.* EpCAM associates with endoplasmic reticulum aminopeptidase 2 (ERAP2) in breast cancer cells. *Biochem Biophys Res Commun* **439**, 203-208, doi:10.1016/j.bbrc.2013.08.059 (2013).

127 Castello, A. *et al.* Insights into RNA biology from an atlas of mammalian mRNA-binding proteins. *Cell* **149**, 1393-1406, doi:10.1016/j.cell.2012.04.031 (2012).

128 Lee, O. H. *et al.* Genome-wide YFP fluorescence complementation screen identifies new regulators for telomere signaling in human cells. *Molecular & cellular proteomics : MCP* **10**, M110 001628, doi:10.1074/mcp.M110.001628 (2011).

129 Powell, A. G., Horgan, P. G. & Edwards, J. The bodies fight against cancer: is human leucocyte antigen (HLA) class 1 the key? *J Cancer Res Clin Oncol* **138**, 723-728, doi:10.1007/s00432-012-1192-4 (2012).

130 Nehme, A., Zibara, K., Cerutti, C. & Bricca, G. 8d.07: Gene Expression Analysis and Bioinformatics Revealed Potential Transcription Factors Associated with Renin-Angiotensin-Aldosterone System in Atheroma. *J Hypertens* **33 Suppl 1**, e115, doi:10.1097/01.hjh.0000467661.76779.b2 (2015).

131 Schmidt, M., Micke, P., Gehrmann, M. & Hengstler, J. G. Immunoglobulin kappa chain as an immunologic biomarker of prognosis and chemotherapy response in solid tumors. *Oncoimmunology* **1**, 1156-1158, doi:10.4161/onci.21653 (2012).

132 Michel, M. A. *et al.* Assembly and specific recognition of k29- and k33-linked polyubiquitin. *Mol Cell* **58**, 95-109, doi:10.1016/j.molcel.2015.01.042 (2015).

133 Jiang, J. H. *et al.* Clinical significance of the ubiquitin ligase UBE3C in hepatocellular carcinoma revealed by exome sequencing. *Hepatology* **59**, 2216-2227, doi:10.1002/hep.27012 (2014).

134 Chu, B. W. *et al.* The E3 ubiquitin ligase UBE3C enhances proteasome processivity by ubiquitinating partially proteolyzed substrates. *J Biol Chem* **288**, 34575-34587, doi:10.1074/jbc.M113.499350 (2013).

135 Zollmann, T., Bock, C., Graab, P. & Abele, R. Team work at its best - TAPL and its two domains. *Biol Chem* **396**, 967-974, doi:10.1515/hsz-2014-0319 (2015).

136 Hassan, M., Watari, H., AbuAlmaaty, A., Ohba, Y. & Sakuragi, N. Apoptosis and molecular targeting therapy in cancer. *Biomed Res Int* **2014**, 150845, doi:10.1155/2014/150845 (2014).

137 Mukhopadhyay, S., Panda, P. K., Sinha, N., Das, D. N. & Bhutia, S. K. Autophagy and apoptosis: where do they meet? *Apoptosis* **19**, 555-566, doi:10.1007/s10495-014-0967-2 (2014).

138 Tian, G. & Cowan, N. J. Tubulin-specific chaperones: components of a molecular machine that assembles the alpha/beta heterodimer. *Methods Cell Biol* **115**, 155-171, doi:10.1016/B978-0-12-407757-7.00011-6 (2013).

139 Voloshin, O. *et al.* Tubulin chaperone E binds microtubules and proteasomes and protects against misfolded protein stress. *Cell Mol Life Sci* **67**, 2025-2038, doi:10.1007/s00018-010-0308-8 (2010).

140 Stolz, A., Besser, S., Hottmann, H. & Wolf, D. H. Previously unknown role for the ubiquitin ligase Ubr1 in endoplasmic reticulum-associated protein degradation. *Proc Natl Acad Sci U S A* **110**, 15271-15276, doi:10.1073/pnas.1304928110 (2013).

141 Summers, D. W., Wolfe, K. J., Ren, H. Y. & Cyr, D. M. The Type II Hsp40 Sis1 cooperates with Hsp70 and the E3 ligase Ubr1 to promote degradation of terminally misfolded cytosolic protein. *PloS one* **8**, e52099, doi:10.1371/journal.pone.0052099 (2013).

142 Matsui, T. & Fukuda, M. Rab12 regulates mTORC1 activity and autophagy through controlling the degradation of amino-acid transporter PAT4. *EMBO Rep* **14**, 450-457, doi:10.1038/embor.2013.32 (2013).

143 Matsui, T., Itoh, T. & Fukuda, M. Small GTPase Rab12 regulates constitutive degradation of transferrin receptor. *Traffic* **12**, 1432-1443, doi:10.1111/j.1600-0854.2011.01240.x (2011).

144 Jiang, H. *et al.* A microtubule-associated zinc finger protein, BuGZ, regulates mitotic chromosome alignment by ensuring Bub3 stability and kinetochore targeting. *Dev Cell* **28**, 268-281, doi:10.1016/j.devcel.2013.12.013 (2014).

145 Toledo, C. M. *et al.* BuGZ is required for Bub3 stability, Bub1 kinetochore function, and chromosome alignment. *Dev Cell* **28**, 282-294, doi:10.1016/j.devcel.2013.12.014 (2014).
